# Colonial bacterial memetic algorithm and its application on a darts playing robot

**DOI:** 10.1038/s41598-025-94245-1

**Published:** 2025-03-28

**Authors:** Szilárd Kovács, Csaba Budai, János Botzheim

**Affiliations:** 1 Department of Artificial Intelligence, Faculty of Informatics, Eötvös Loránd University, Pázmány P. sétány 1/A, Budapest, Pest 1117 Hungary; 2 Department of Mechatronics, Optics and Mechanical Engineering Informatics, Faculty of Mechanical Engineering, Budapest University of Technology and Economics, 4-6 Bertalan Lajos Street, Budapest, Pest 1111 Hungary

**Keywords:** Memetic algorithm, Robotics, Continuous optimization, Constrained optimization, Multi-objective optimization, Self-adaptive optimization, Electrical and electronic engineering, Computational science

## Abstract

In this paper, we present the Colonial Bacterial Memetic Algorithm (CBMA), an advanced evolutionary optimization approach for robotic applications. CBMA extends the Bacterial Memetic Algorithm by integrating Cultural Algorithms and co-evolutionary dynamics inspired by bacterial group behavior. This combination of natural and artificial evolutionary elements results in a robust algorithm capable of handling complex challenges in robotics, such as constraints, multiple objectives, large search spaces, and complex models, while delivering fast and accurate solutions. CBMA incorporates features like multi-level clustering, dynamic gene selection, hierarchical population clustering, and adaptive co-evolutionary mechanisms, enabling efficient management of task-specific parameters and optimizing solution quality while minimizing resource consumption. The algorithm’s effectiveness is demonstrated through a real-world robotic application, achieving a 100% success rate in a robot arm’s ball-throwing task usually with significantly fewer iterations and evaluations compared to other methods. CBMA was also evaluated using the CEC-2017 benchmark suite, where it consistently outperformed state-of-the-art optimization algorithms, achieving superior outcomes in 71% of high-dimensional cases and demonstrating up to an 80% reduction in required evaluations. These results highlight CBMA’s efficiency, adaptability, and suitability for specialized tasks. Overall, CBMA exhibits exceptional performance in both real-world and benchmark evaluations, effectively balancing exploration and exploitation, and representing a significant advancement in adaptive evolutionary optimization for robotics.

## Introduction

Optimization methods play a crucial role in solving complex problems across various scientific and engineering domains. Particularly in robotics, achieving high efficiency and adaptability in optimization algorithms is essential for addressing real-world challenges, including dynamic environments, constraints, and multi-objective requirements. Among these methods, Evolutionary Algorithm (EA)s have gained prominence due to their ability to mimic natural selection processes to find optimal or near-optimal solutions in complex search spaces.

**Fig. 1:**
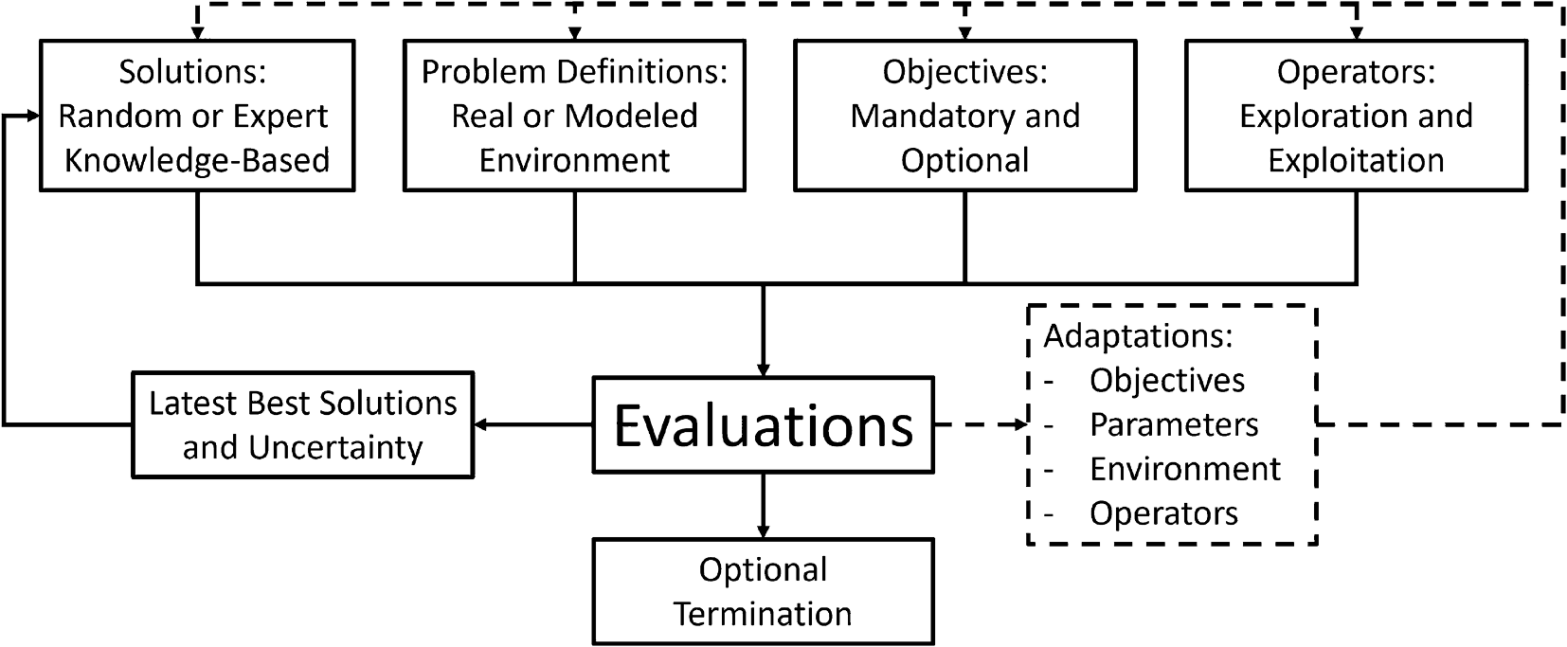
General evolutionary optimization process. The diagram illustrates the iterative nature of evolutionary algorithms, including solution generation, problem definition, evaluation, and adaptation mechanisms.

EAs are a class of population-based optimization methods inspired primarily by biological evolution. These algorithms employ mechanisms such as selection, mutation, recombination, and reproduction to evolve a population of candidate solutions over successive generations. By balancing exploration of the global search space with exploitation of promising regions, EAs are particularly effective for high-dimensional and multimodal optimization problems. Common types of evolutionary algorithms include Genetic Algorithm (GA)s, Particle Swarm Optimization (PSO), Differential Evolution (DE), and Bacterial Evolutionary Algorithm (BEA), each offering unique mechanisms for addressing specific problem types. While EAs excel in parallel execution and adaptation capabilities, which are key strengths of the original concept, these aspects have received comparatively less emphasis in recent research trends. Currently, in the field of optimization, the focus is on conditional multi-objective hierarchical problems. Connecting to real-world uncertainty is also a challenging factor. Two additional aspects can be highlighted that are important for the development of good optimization: keeping complexity under control and maintaining the possibility of development, which is very rare in the case of modern optimization algorithms. Current adaptive algorithms can satisfy either fast (exploitation) or evolving (exploration) solutions. Figure 1 provides a unique overview of EAs focusing on parallel execution, adaptation, continuous process, and uncertainty. The listed difficulties require a more complex organization of individuals. In the article, a new hierarchical population organization is presented for a more efficient flow of information. Hierarchical population organization enables the local adaptivity of individuals. The more efficient flow of information makes it possible to increase the complexity of the individuals, so the individual’s next state can be determined using the condensed information from the population at the individual level. More complex individuals also provide an opportunity for statistical sampling so that more reliable information is transmitted to higher organizational levels. A further innovation of the algorithm is that individuals received individual goals, with the addition that they are able to evaluate each other’s strategy with the help of a common “language”.

**Fig. 2:**
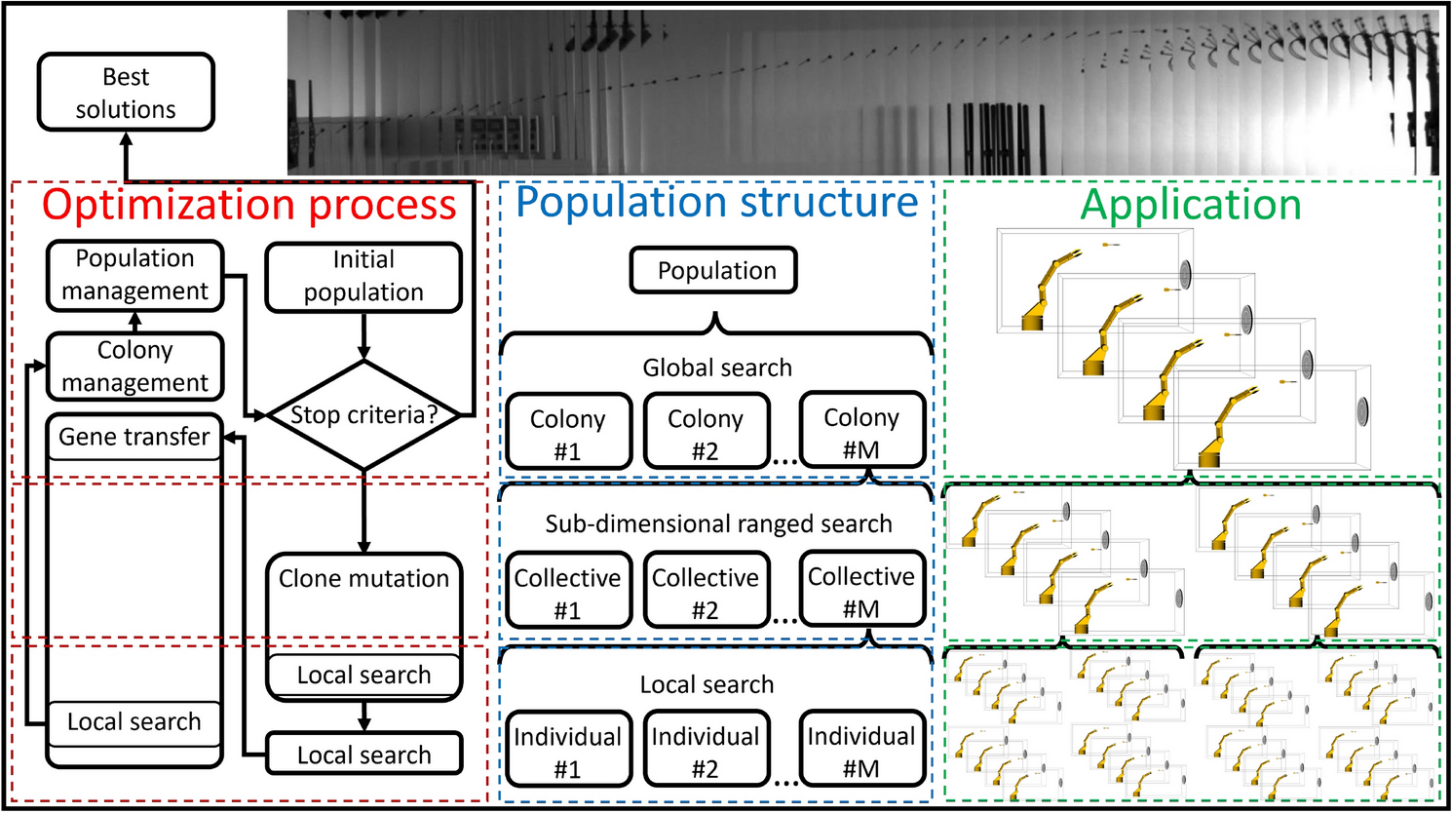
Overview of the CBMA framework. The diagram illustrates the optimization process, population structure, and its application to darts learning. A 3D illustration of the simulation^1^ and a montage of the results are shown at the top. The illustrative 3D figures were created in Microsoft PowerPoint (Version 2501) using simple shapes^2^. The mathematical models used in the framework are also available as indicated in the Code Availability statement.

This paper introduces the CBMA, an advanced evolutionary optimization approach designed to overcome these challenges, illustrated in Fig. 2. CBMA extends the Bacterial Memetic Algorithm (BMA) by incorporating principles of Cultural Algorithms and co-evolutionary dynamics inspired by bacterial group behavior. Through hierarchical population organization and adaptive mechanisms, CBMA effectively balances exploration and exploitation while handling constraints, multiple objectives, and large search spaces with efficiency. The CBMA algorithm is built upon several innovative features. By organizing individuals into colonies and collectives, CBMA enables efficient information flow and adaptability at multiple levels. The algorithm’s modular design allows for task-specific parameter management and optimization while minimizing resource consumption.

The rest of the paper is organized as follows. In “Literature review”, the related literature is presented from single objective to multi-objective optimization from different perspectives. In “Colonial bacterial memetic algorithm”, the developed algorithm is presented. In “Algorithm summary and algorithm comparison”, the developed algorithm is compared statistically with other algorithms. In “Real-life example: darts”, the algorithm’s applicability is demonstrated on a real problem.

## Literature review

This section describes current optimization methods. The optimization methods can be grouped based on several aspects: adaptivity, evolvability, decomposability, memetic algorithms, condition handling methods, single- and multi-objective, etc. The listed aspects are summarized in this chapter.

### Adaptivity

Adaptivity is a defining characteristic of EAs. Static adaptation can be achieved by defining different population segments, as in Dung Beetle Optimizer (DBO)^3^. Instead of relying on static settings, adaptive EAs modify parameters or operators to respond to performance metrics, environmental changes, or internal feedback signals. A widely used technique is to tie parameter schedules to the iteration count or ratio, gradually shifting from exploration to exploitation^3–8^. Usual parameters noise factor maintains diversity in the early stages, and the dissipation rate regulates convergence speed, preventing premature stagnation. This mechanism optimally balances search intensity, improving both exploration efficiency and final solution precision. In addition to the monotonous scheduler for extended training and complex data representation^9^ sinusoidal scheduling parameters are also available. This approach is straightforward when the total number of generations is known or when strict computational budgets exist. However, it can become problematic in open-ended scenarios where the optimum horizon is unclear, since halting too soon might miss global optima, and continuing unnecessarily can waste valuable resources.

A second strategy embraces feedback-based adaptivity, where the algorithm monitors real-time performance indicators such as fitness improvements, population distribution^4^, success rates of variation operators, or error divergence^10–13^. When co-evolutionary architectures are used, multiple populations exchange information or refer to a shared population to guide adaptivity^14,15^. These mechanisms can also trigger restarts to counteract premature convergence and reinvigorate the search with fresh diversity^16^. A complementary approach leverages dynamic boundary adjustments, where search regions evolve per individual based on local and global best solutions, balancing exploration and exploitation. One solution for boundary adjustment use the integrating linear scaling and chaotic mapping to preserve diversity while refining promising regions, with poorly performing individuals repositioned to prevent stagnation as implemented in Multi-Strategy Dung Beetle Optimizer (BGADBO)^4^. Further enhancements, such as oscillating balance factors, fractional-order calculus, adaptive fractional orders, and dimension-by-dimension mutation with t-distributions, dynamically regulate search intensity and refine solution adjustments^17^. These mechanisms enable fine-grained control over exploration and exploitation, improving convergence stability and solution quality in large-scale, high-dimensional problems.

Among these many adaptive approaches, self-adaptive EAs stand out for encoding strategy parameters directly in each individual’s genetic makeup^18,19^. Instead of requiring external updates or deterministic schedules, self-adaptive schemes evolve control parameters alongside solution variables, letting successful parameter configurations propagate naturally in the population. Methods such as self-adaptive differential evolution^19^ demonstrate how mutation rates, crossover probabilities, or even learning rates can be tuned automatically. This becomes particularly advantageous when problem landscapes are highly non-linear or change over time, as the algorithm continually re-evaluates both parameter settings and candidate solutions in tandem.

### Evolvability

Continuability/improvability/evolvability of a solution is a critical consideration in the case of complex tasks or large-scale optimization. Three important questions can be asked regarding the running of the algorithm: How do we start? How do we continue? When will we finish? For the first question, the majority of the real-world problems are partially black-box problems^20^, so the available information is limited. Effective initialization often relies on input from human or machine experts to incorporate partial, potential, and known solutions, thereby accelerating the evolutionary process. This approach highlights the distinction between real-world problems and test functions, where random initialization is typically required in the literature for fair comparisons. For instance, a problem-specific optimization technique, such as uniform distribution-based initialization, is particularly useful for lower-dimensional problems^21^. Many specific initializations are available for particular purposes like neural networks or more general search problem^22^. For the stopping criterion, it is common to use the error/divergence/objective function, fixed iteration number, or fixed time. The most interesting is the second question, how to continue both after the initialization and if we are not satisfied with the stopped result or if new opportunities have opened up to continue. One possible implementation of continuation is the use of a deterministic algorithm. Deterministic algorithms are susceptible to initialization, so initialization is typically based on expert knowledge or sampling. Bayesian optimization^23^ and its variants are most typical for global search. The disadvantage of deterministic algorithms is that their complexity increases significantly as the number of dimensions increases. Another method of continuation is the use of memory. Memory can be either full or selective. Selective memory can be used by hibernation^24^, reincarnation^25^, restart, etc. The selection can be implemented using the selection methods common in EAs, clustering, based on diversity, etc.

### Memetics

The memetic algorithms combine local and global search techniques. In general, local search is more resource-intensive than global search. Local search is usually performed by deterministic algorithms and relies on the derivative, such as Gradient descent, Gauss-Newton, Quasi-Newton, Levenberg-Marquardt (LM) algorithm^10,11^, Newton-Raphson-Based Optimizer (NRBO)^26^ etc. There are also derivative-free, sampling-based variants available, such as discrete local searches, Pattern search^27^, Nelder-Mead method^28^, Simultaneous perturbation stochastic approximation (SPSA)^29^, GradientLess Descent^30^, etc. For global search, mainly combinatorial search methods are used such as GA, BEA^31^, Ant Colony Optimization^32^, Taboo search^33^, etc. Between the gradient-based and combinatorial approaches, many methods can take into account the direction of decline at the population level depending on their parameters, such as PSO^34^, Cuckoo Search^35^, Natural Evolution Strategies^36^, DE^37,38^, JAYA^39^, Fire Hawk Optimizer (FHO)^40^, Aquila Optimizer (AO)^5^, Golden Eagle Optimizer^6^, Arithmetic Optimization Algorithm (AOA)^41^, Marine Predators Algorithm (MPA)^42^, Artificial Bee Colony^43^, Black Widow Optimization Algorithm^44^, etc. With different parameterizations, the “population-level gradient-based” methods can also be combined to create a quasi-memetic algorithm^45,46^.

Many population-based algorithms utilize hierarchical structures to guide their search process. For example, Grey Wolf Optimizer (GWO), FHO, and Greater Cane Rat Algorithm^47^ use leader-follower hierarchies to balance exploration and exploitation. These methods dynamically balance exploration and exploitation through structured leadership, improving their ability to escape local minima while converging on global optima, and that is the most crucial question of memetic algorithms.

### Dimension reduction

Dimension reduction is common in the optimization of large models^48,49^, so optimizations are performed in sub-dimensions, where the number and structure of sub-dimensions are often questionable. Mapping in sub-dimensions can be achieved using various techniques, including neural networks^50^, principal component analysis^51^, GAs^52^, feature selection^53^, BEA^31^, or even random methods. More recently, nature-inspired algorithms such as Human Learning Optimization, Poor and Rich Optimization, and GWO have gained attention for their effectiveness in feature selection across various datasets, demonstrating significant improvements in feature reduction with maintained accuracy for classification^54^. A structured dataset gives more room for dimension reduction, a good example is the spacial relation^55,56^. Another essential aspect of dimension resolution is the NK-Landscape problem, which has important implications for optimization. The choice of sub-dimensions should be made with this in mind^57^. These algorithms offer a balance between feature count, classification accuracy, and computational cost, providing robust solutions for feature selection and dimensionality reduction in large-scale data. Most important questions: How many groups and what group composition are good for the problem? Is it possible to keep one composition, or should it be dynamically regrouped?

### Uncertainty

There can be various reasons for the uncertainty, such as temporality, imprecise modeling, neglected and unaccountable effects, and numerical representation. In all cases, sampling plays an important role. Combined with optimization, it can significantly increase the evaluation time of a model. The appropriate number and type of sampling depends on the task. In addition to evaluation, sampling is also necessary in controlled adaptive algorithms. Statistically appropriate sampling is not necessary in many cases, like in the initial stages of optimization. However, it can lead to errors when comparing individual solutions. A good solution to the problem can be increasing sampling in combination with the forgetting operator. In the case of adaptive control parameters, the method of inheritance and moments can be used instead of increasing sampling, limiting the complexity of the algorithm. The forget operator is also advantageous due to the sampling.

### Constraint management

Constraint handling is critical in ensuring the feasibility of solutions in optimization problems, and several methods exist to manage these constraints^58,59^. A best practice in constraint handling is to avoid constraints altogether by transforming the problem space. For example, manifold-based techniques use a different space representation to embed constraints within the geometry of the space, eliminating the need for added penalties or explicit constraint-handling mechanisms^60^. The most common approach is the direct addition of constraints to the objective function, often using a penalty-based system^14,61^. This method integrates constraint violations into the cost function, with penalties increasing in proportion to the degree of violation, gradually guiding the population toward feasible regions^62^. The penalties can formed as soft or strict boundaries.

In addition, partial or temporary relaxation of constraints is employed in some methods to improve the exploration of the search space, especially during the early phases of optimization. This relaxation can be applied either intermittently or solely to a subset of the population, allowing more flexibility in discovering potential solutions^63–65^. Such approaches enhance the algorithm’s ability to escape local optima by exploring infeasible regions temporarily before progressively enforcing stricter constraint adherence. Despite the improvement of exploration, weakening conditions or completely omitting could lead to instability.

The choice of constraint management method depends on the problem’s complexity and the nature of the constraints.

### Multi-objective optimization

The core of multi-objective optimization is provided by single-objective methods. In some cases, multi-objective tasks are also directly reduced to a single objective one. For multi-objective cases, special additions can be made for a more efficient Pareto front exploration. For example, feature extraction and individual importance degree help maintain diversity and strengthen convergence by applying structured selection pressure within Pareto layers, while the repulsion field method ensures an even distribution across the Pareto front by preventing clustering and diversity loss^66^. Several techniques^67,68^ have been developed for handling multi-objective functions, such as weighting, weighted-sum, trade-off, reference point, etc. The main difference between the methods is the hierarchy of the objectives and the distribution of the population. Most real-world tasks include both hierarchical and non-hierarchical goals. In most cases, uniform coverage of the Pareto front is wanted in multi-objective optimization. One of the most popular methods for optimizing multi-objective functions is the Weighted-Sum Approach (WSA). The WSA method is only applicable for convex functions and requires good initialization to explore the Pareto front^69,70^. Several direct clustering methods have been used to increase exploration, such as reference-point-based^71^ for known Pareto fronts or direction-based^72^, or different distance metrics^73^. The Pareto front is not necessarily continuous. Due to conditions, the Pareto front often breaks down in the objectives’ space. Clustering is also often used to separate the subdivisions of the Pareto fronts, and the population is partitioned accordingly. Typically, density-based algorithms are used. The most widely used is the Density-based spatial clustering of applications with noise^74^, an iterative, slower solution that provides human-like clustering. Its newer version the Hierarchical Density-based Cluster Selection^75,76^ is not yet widely used in optimization. Indirect clustering methods maintain diversity with different weightings and/or special mutation and crossover operators. These methods do not give perfect results, but they also have a lower computational cost.

In the next section, our proposed algorithm is described in detail and compared based on the areas and problems listed in this section.

## Colonial bacterial memetic algorithm

This section presents the CBMA and Constrained Multi-Hierarchical—Colonial Bacterial Memetic Algorithm (CMH-CBMA) to multi-objective hierarchical constrained tasks. The basis of the structure of the CBMA algorithm is the BMA^77^. The BMA was restructured and expanded. The section covers the similarities with nature, the structure of individuals and objectives, the parts of the algorithm, and the differences and similarities with other algorithms being developed recently.

### Algorithm illustration

In nature, there are varying degrees of local communication/organization. Bacteria are no exception. The proposed algorithm aims to transform the existing BMA with this approach. The organization of bacteria into a colony effectively gives the colony the ability to adapt. Outside the bacterial colony, the interaction of each colony can also be observed. Sekowska et al. studied different bacterial clades for collective behavior in terms of merging or repelling^78^. Grobas et al. reveal how can the collective behavior of bacteria provide antibiotic resistance^79^. The interaction of bacterial colonies with each other is well applicable in the search process. By overcoming the limitations of natural processes, artificial elements are added to the optimization to incorporate the organization of the whole population.

**Fig. 3:**
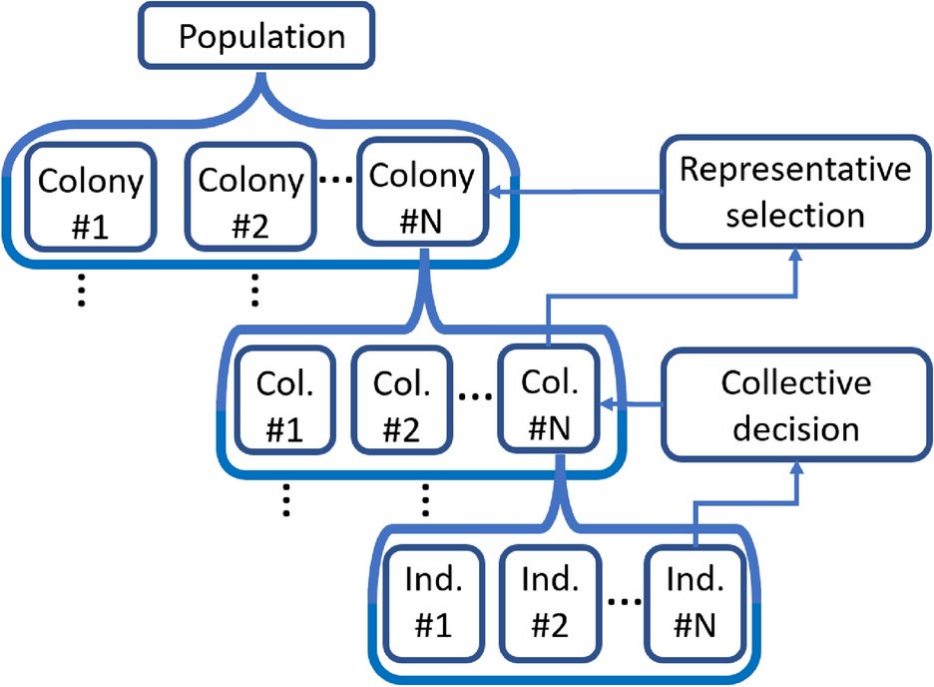
Hierarchical population organization and information compression for more effective communication. We distinguished three levels: local, subdimensional limited, and global.

The population is organized into three levels, as shown in Fig. 3, with each optimization unit targeting one of these levels. The transition between levels represents information compression and summarization. The information gathered by individuals is summarized in the collectives. The most successful collective then becomes the representative of the colony. Colonies make up the whole population.

Table 1 shows the levels of the population and the corresponding operations and objectives. The table also includes the names used in the BMA^77^ algorithm for comparison.

**Table 1:** The table shows an overview of the CBMA algorithm, highlighting its relationship with the BMA algorithm, individual optimization modules, search areas, and the different goals.

Levels	CBMA levels	BMA similarity	Optimizations	Search area	Objectives
1st	Population	Population	Population management	Global (remove/add colonies)	Multi-objective function
2nd	Colony	Individual	Gene transfer, Colony management	Global	Different goals
3rd	Collective	Clones	Bacterial mutation	Middle	Inherited goals
4th	Individual	Local search	Local search	Local	Inherited goals

**Fig. 4:**
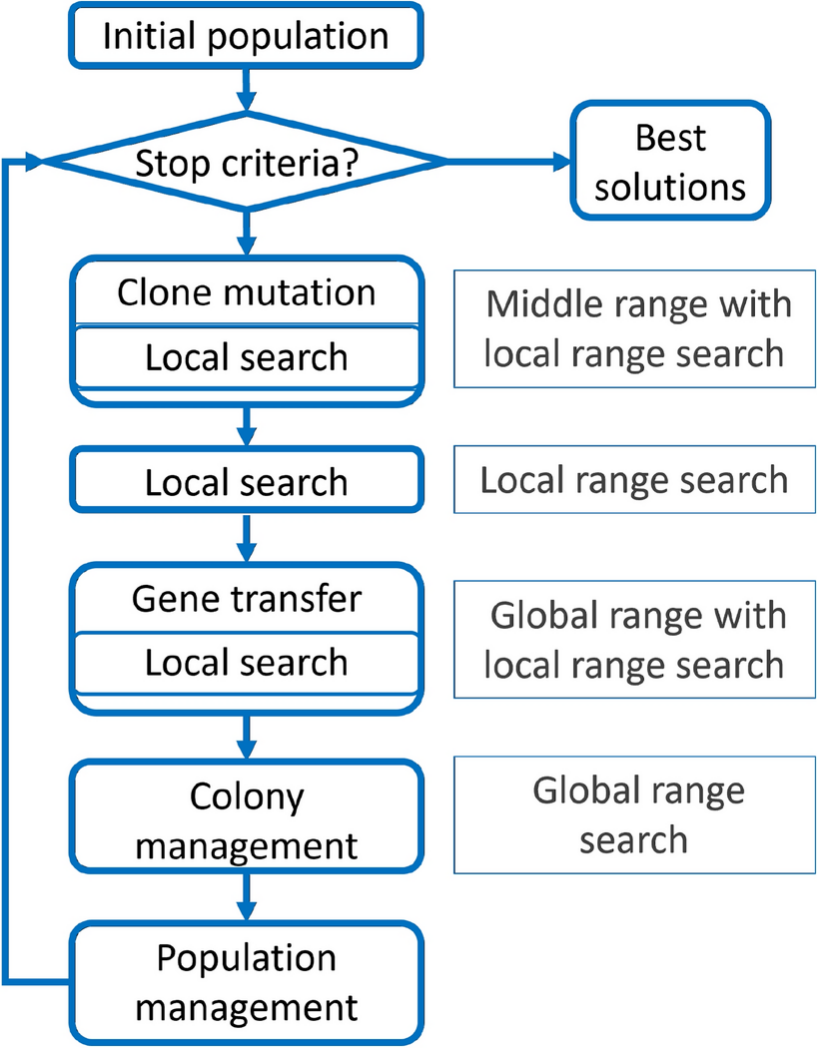
The flowchart of the CBMA algorithm with the main modules.

Figure 4 shows the optimization process with the addition of territorial competencies. Each operation will be discussed in detail in “Components and innovations of the algorithm”. The following subsection shows the structure of individuals and costs.

### Individuals and populations

**Fig. 5:**
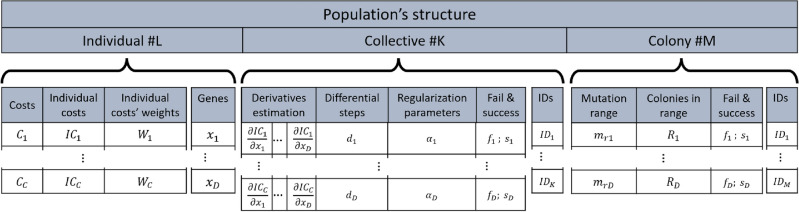
The structure of the population and information content. The local information is summarized in the collectives, then the information is condensed by the distinguished individual. In addition to the common properties, each individual also stores its own special properties.

In most algorithms, individuals are represented as one-dimensional vectors of genes. At the heart of the new algorithm are the individuals and their organizations. Different amounts of data are available at different levels of the population. Individuals have only local/point properties, collectives already have point and environmental properties, and colonies already have regional properties. Individuals have default costs, individual costs with associated normalized weight vectors, and position. In “Real-life example: darts”, the expected value is used to characterize individuals due to probability distribution. The most efficient individuals, the partial derivative estimate, the characterization of the distribution used to estimate, the regularization parameter, the unique identifier, and the collective success rate are presented for collectives. The regularization parameter is necessary for the adaptivity of the local search, discussed in “Local search”. Colonies contain the most successful collectives, mutation range, nearby colonies, colony success characterization, and colony identifier. The population’s structure is illustrated in Fig. 5. The *K* number of collectives does not necessarily have complete information for a total derivative estimation regarding the *C* number of costs and *D* number of genes/dimensions. The *M* number of colonies also does not necessarily have complete information. $W_i$ describes the current weight factor of the “common” cost ($C_i$), $IC_i$ is the individual cost, $d_i$ is the differential step size, $\alpha _i$ is the regularization parameter for the LM algorithm, $f_i$ and $s_i$ are fail and success counts in local search and collective levels, $m_{ri}$ is the mutation range for clone mutation, and $R_i$ is checking if another colony is close.

**Fig. 6:**
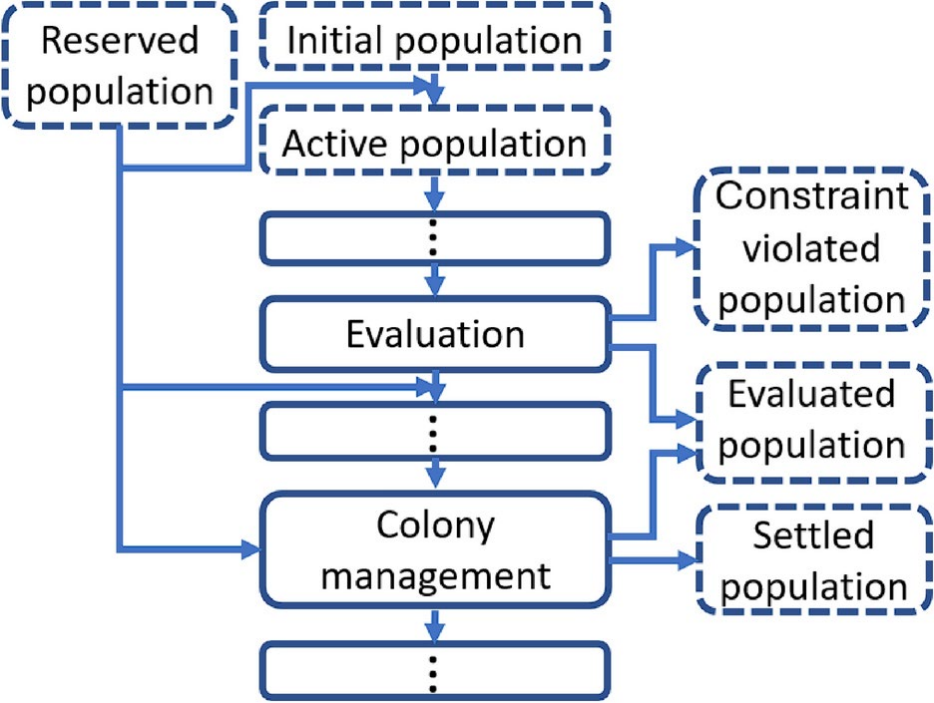
Different populations for different purposes. The algorithm contains six different populations, so a distinction is made between the initial population, the reserved population, the active dominant individuals participating in the search (Active population), the individuals violating some condition at the global level (Constraint violated population), the non-dominant individuals fulfilling the conditions (Evaluated population), and the dominant, non-developing individuals (Settled population).

Six types of populations are part of the search algorithm as illustrated in Fig. 6. The initial population contains the initial colonies, which later form the active colonies that participate in the search. In addition to the initialized population, a reserved population is also available to replace individuals that violate the conditions. The reserved population allows additional colony initialization and provides replacement in a constraint violation. Its size equals the possible number of parallel evaluations and is replaced if it runs out. Collectives that meet the conditions under consideration are saved and can later be used to initialize new individuals. Collectives that violate the constraints are recorded separately. In colony management, finished colonies are kept.

### Cost’s structures

**Fig. 7:**
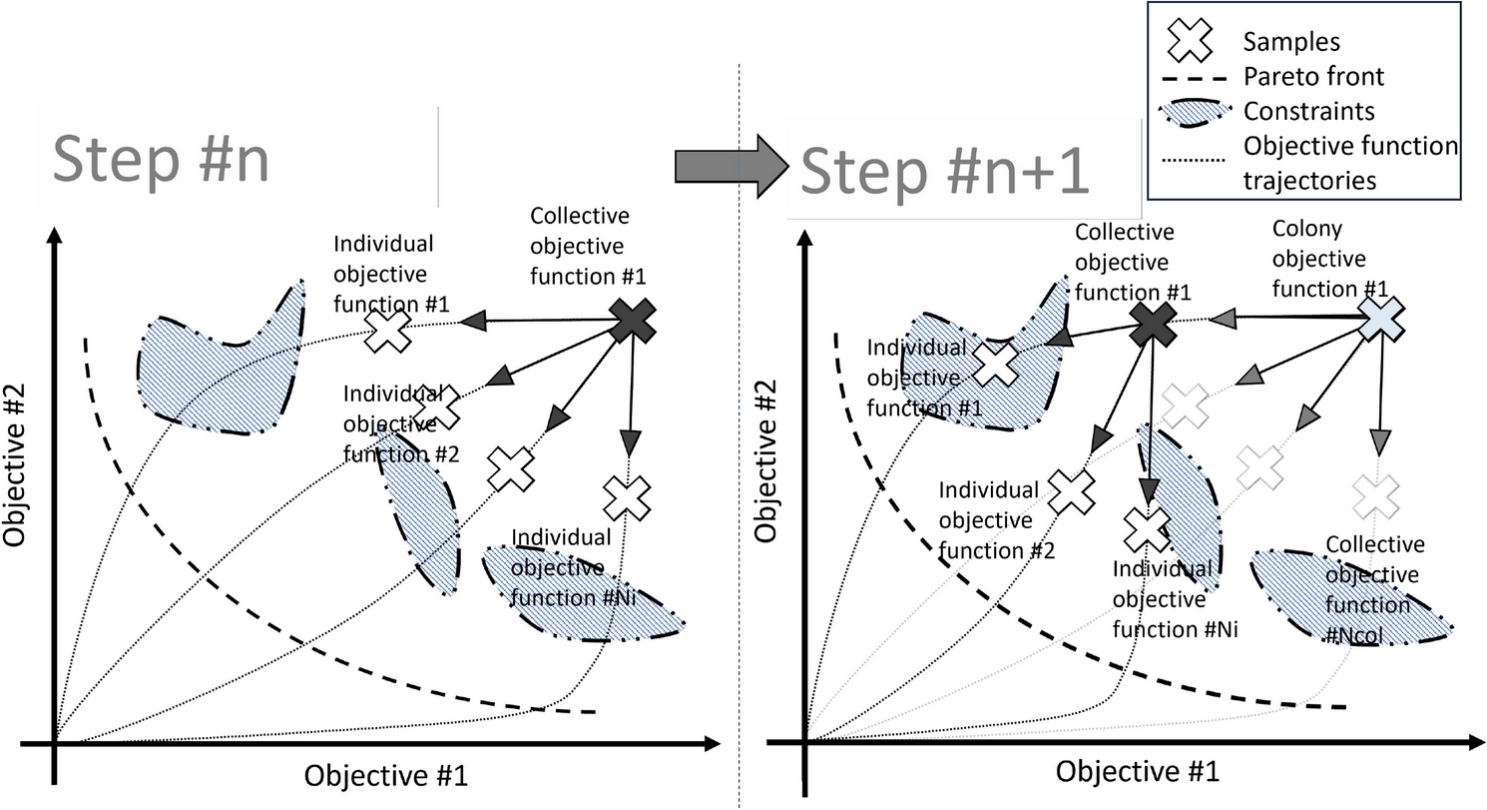
Illustration of the variable multi-weighted sum approach. The approach makes the search more robust and helps to explore the solution space without a random factor.

In the case of multiple cost components, the cost weighting may not be known, or not only one solution is sought. Instead of the clustering algorithms listed earlier, the hierarchical structure of the population is used to cluster the individuals so that it is not necessary to sort them at each iteration. The simple weighted sum approach has been extended with individual variable weights, so each colony searches according to its weights until it receives a new target. The new method is called the Variable Multi-Weighted Sum Approach (VMWSA). The operation of VMWSA is illustrated in Fig. 7 for the two-dimensional case. The simple method also provides a solution for the concave discontinuous Pareto fronts presented earlier. It is important to note that constraints can be added to the list of objectives, so the VMWSA not only provides solutions for multi-objectivity but also for constraint management.

### Components and innovations of the algorithm

In this subsection, parts of the algorithm are presented. The algorithm consists of the units shown in Fig. 4. Compared to the initial BMA^77^ algorithm, the sub-units have been changed, and two new sub-units have been added. Each operation is presented in the order of clone mutation, gene transfer, local search, colony management, and population management.

#### Clone mutation

Compared to the original Bacterial mutation in BMA^80^, more adaptability has been added to the clone mutation. The mutation’s strength is updated based on the success of the mutation step in each colony. After a successful mutation cycle, the mutation’s strength decreases; after an unsuccessful one, the mutation’s strength increases. It helps to move faster if colonies could not improve in their region and slow down if the area has more potential. The selection of gene groups is examined at the population level, considering each gene group’s performance. The new gene groups are generated externally in the population management sub-unit based on the gene group’s performance. A GA^81^ is used to search sub-dimensions to maximize the improvement per simulation. The local search is changed to a sub-dimensional and performed along the selected dimensions during the clone mutation. This method made the adaptive sub-model creation possible. Changing bacterial mutation and creating individual target functions has strengthened the coevolutionary^82^ approach in BMA.

Instead of a random probability, the local search is performed only on a specified percentage of the best clones/collectives to consider the Pareto principle^83^. A small percentage of unselected collectives swap places with selected collectives to reduce elitism. The collectives are sorted before the local search, and the original order is saved. After the local search, the collectives are rearranged to keep the relationship between the original colonies and their clones/collectives. The reduced number of local searches increased the efficiency of the mutation phase. The new clone mutation phase is shown in Alg. 1.

**Algorithm 1:**
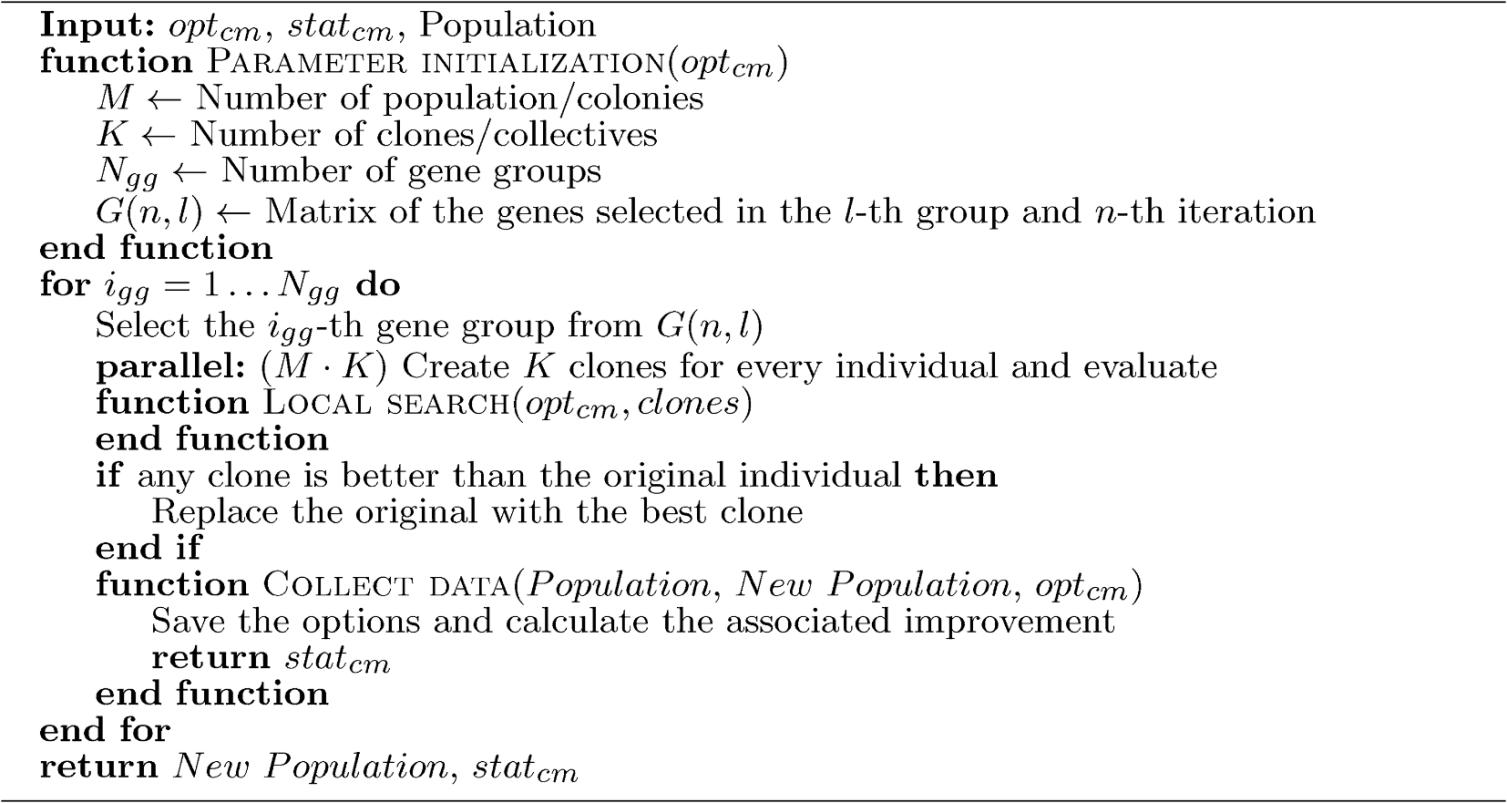
Modified clone mutation with local search

#### Gene transfer

The originally sequential gene transfer is parallelized, and a local search is added to it. A sub-dimensional local search is performed along the selected dimensions during the gene transfer. The new gene transfer is shown in Alg. 2. To keep diversity^63^, a small number of gene transfers were used, which did not allow statistically acceptable sub-model selection, so gene transfer was still performed using randomly chosen genes. The sub-models used in clone mutation and the different information exchange procedures will be investigated later.

**Algorithm 2:**
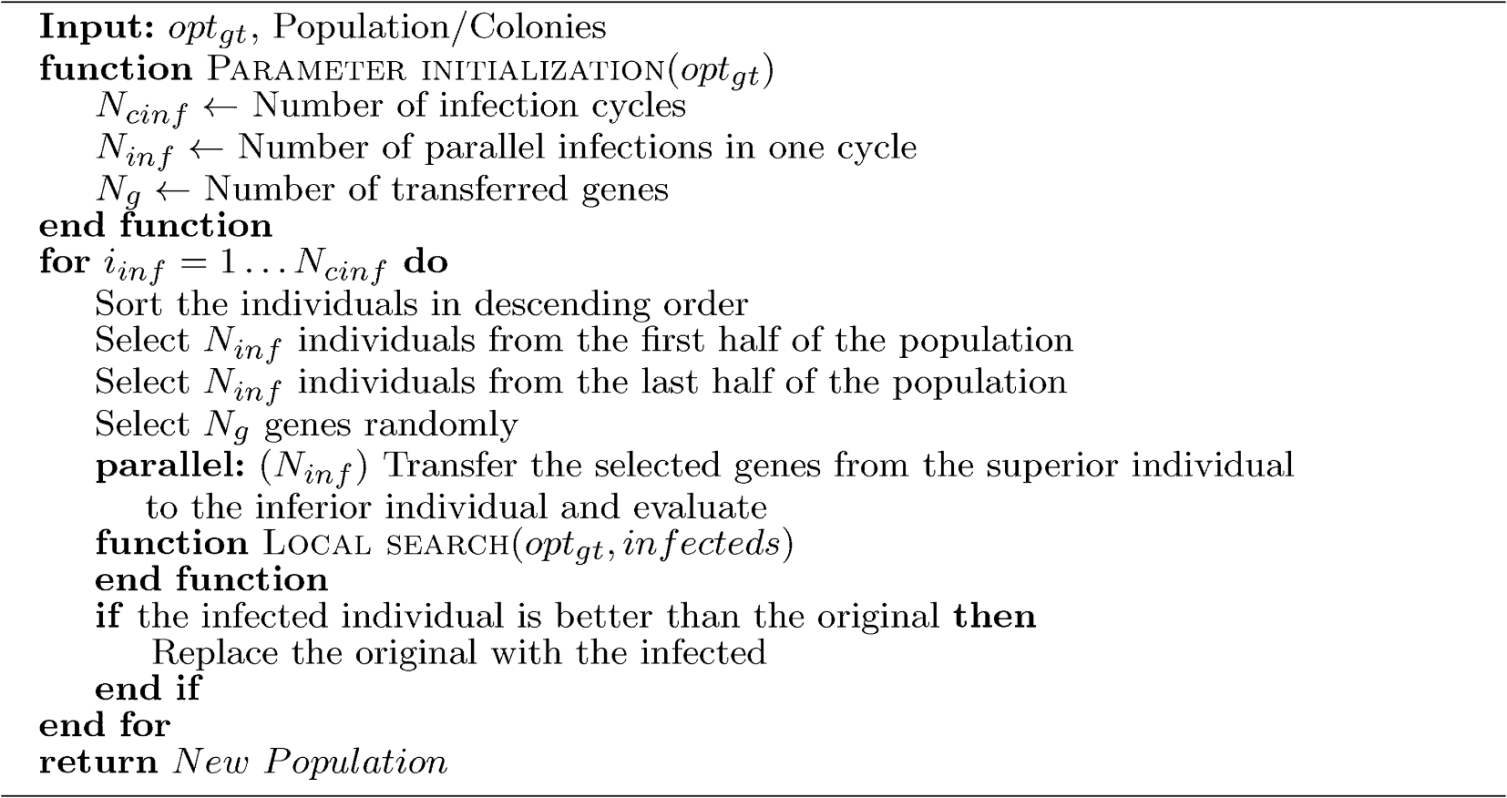
Gene transfer with local search

#### Local search

**Fig. 8:**
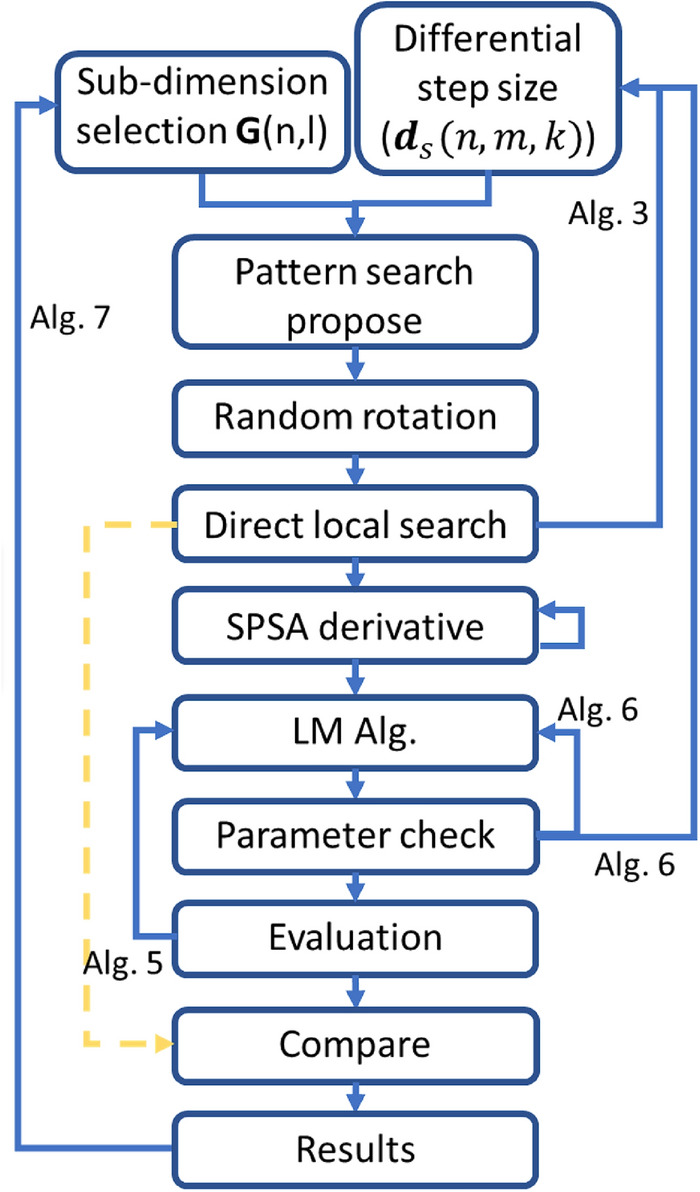
Combined local search method. The focus of the search is Patent Search, SPSA, the LM algorithm and its variants, all of which are suitable for local search and complement each other well. The computational requirements of the most expensive algorithm, LM, were limited and the other algorithms were selected so that they did not require significant extra computation.

Local search is a cornerstone of memetic algorithms. Choosing the right one is a difficult task. In addition to its selection, an equally important question is: Is it worth selecting/changing? The most straightforward selection can be achieved by connecting algorithms in parallel, but they can also be connected in sequence or nested. The use of adaptive algorithms gives flexibility between some local search procedures so that it is not necessary to select algorithms. The local search procedure combines the Pattern search^27^, the direct local search, the SPSA^29^, the Gauss-Newton algorithm, and the gradient descent algorithms. The complete simplified process is shown in Fig. 8. The Pattern search has been complemented with rotation to make it more robust. The entire process is performed along sub-dimensions, with sub-dimension selection, memory, and momentum added. The process is initialized by pattern search using a one-sided distribution, and then random rotation is used to evolve the process into a ring distribution, if necessary. After the direct search, a gradient estimation is performed based on the ring distribution as in the SPSA^29^. LM algorithm^10,11^ can be executed with gradient estimation. Finally, the results of the direct and LM searches are compared. After unsuccessful local search steps, the distribution used for the derivative estimation evolves. In case of success, momentum can be added to the process so that a sub-dimension can affect the local search along with other sub-dimensions. Momentum is an efficient method for batch-based (sub-dimension-based) optimization.

**Fig. 9:**
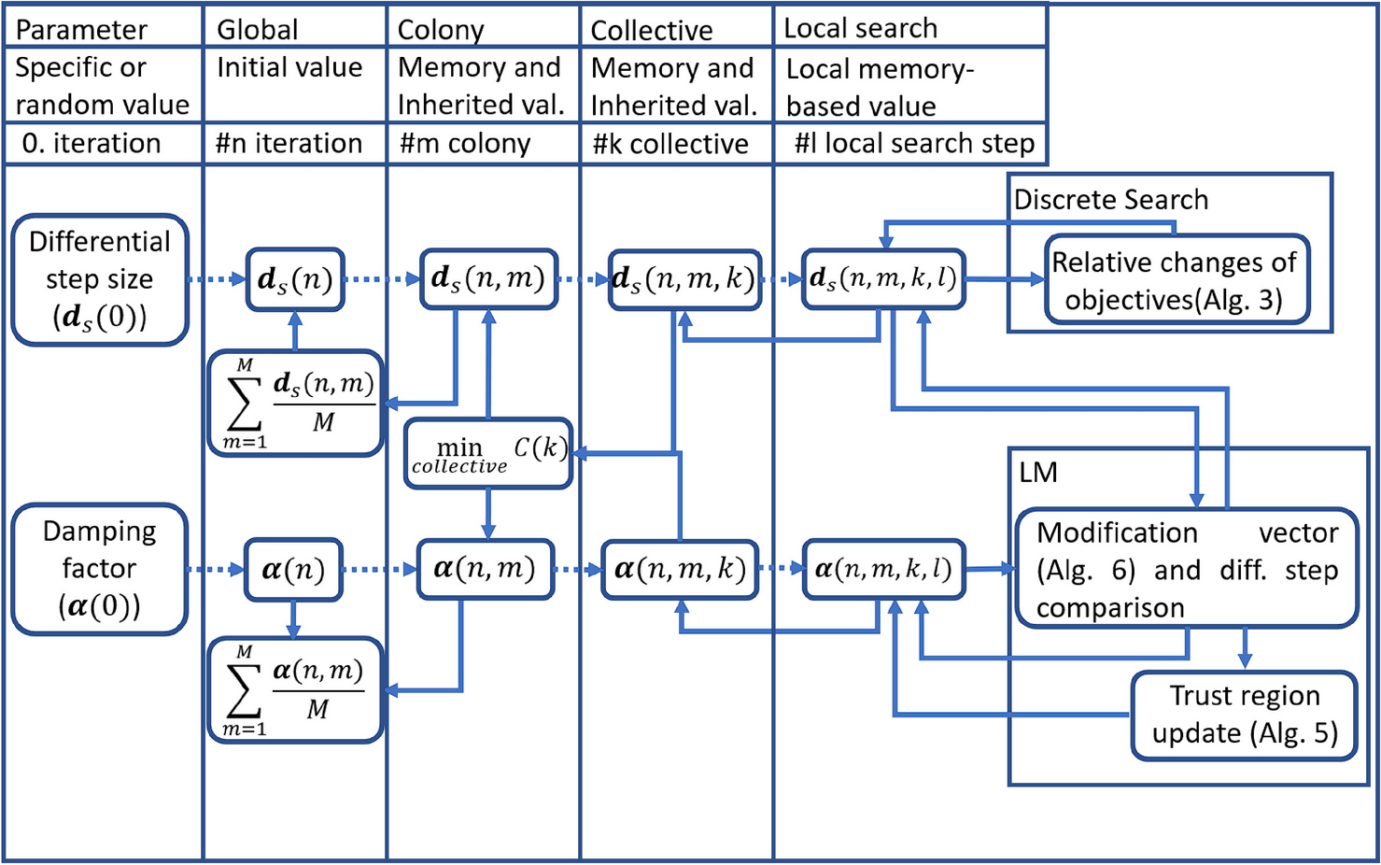
The figure shows the cooperation between the step size and damping factor. At the lower local level, individuals have the opportunity to adapt based on simple feedback, and then an aggregated adaptivity reaches the higher levels of the population.

The implemented feedback control provides an adaptive and robust local search. The most important aspect of the process is the bond between its parameters. The strongest connection can be established for deterministic algorithms. Figure 9 describes the relationship between differential step size and damping factor.

**Fig. 10:**
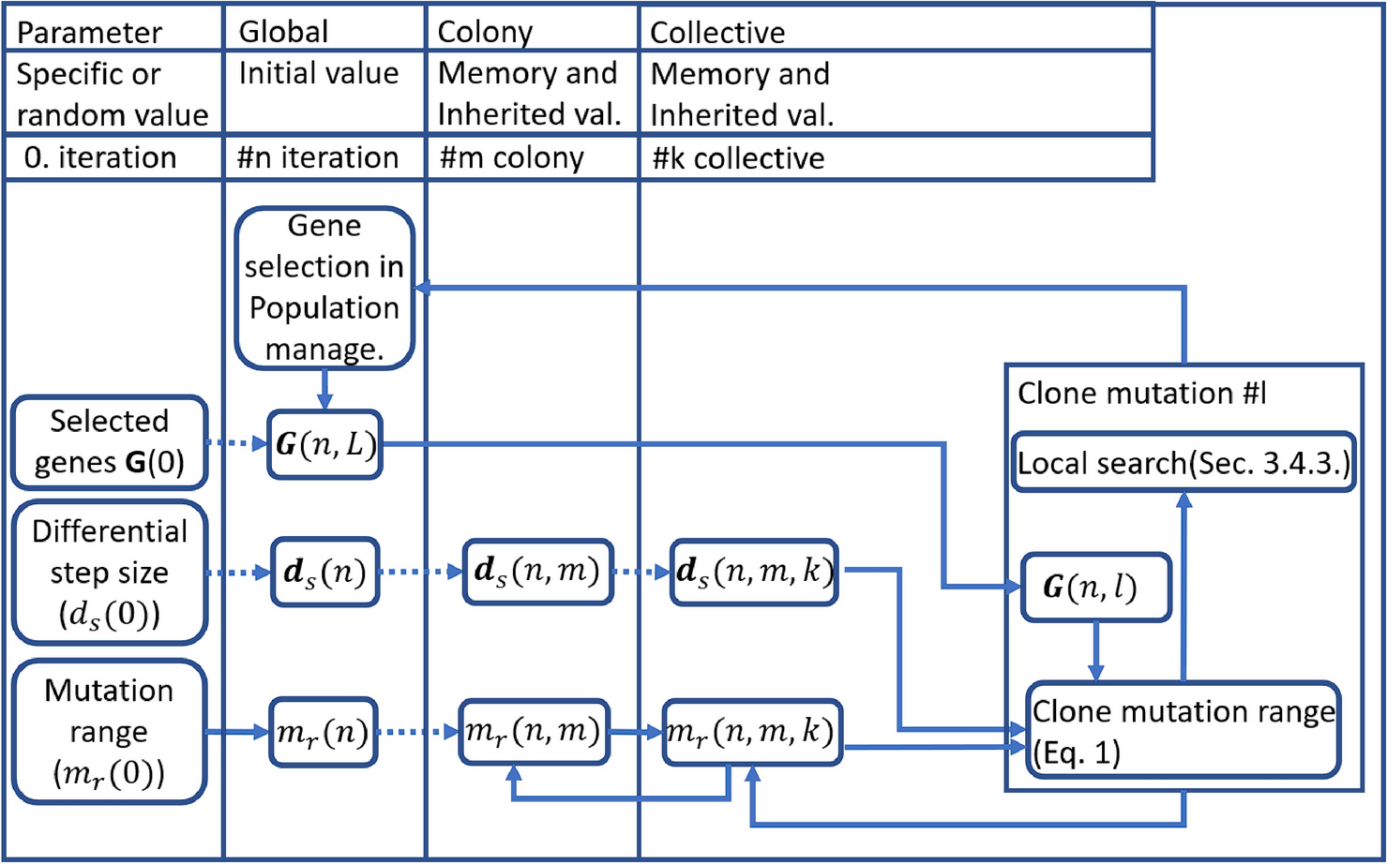
The figure shows the combined local search and clone mutation. Similarly, the goal was to be able to connect the modules as much as possible without causing a significant increase in computing requirements. Furthermore, it should be possible for local adaptivity and the dissemination of acquired knowledge.

In Figs. 9 and 10, the dotted lines represent an initialization relationship. In each case, parameters are fed back individually at each operation.

At the end of a sub-process, the joint information is compressed using the best or expected value and additional data, shown in “Individuals and populations”. The feedback dramatically reduces the importance of initialization.

Figure 10 shows the relationship between mutation and local search. Gene selection selects genes based on the combined performance of clone mutation and local search, described in “Colony and population management”. The strength of the mutation ($m_r$) in each dimension is inversely proportional to the differential step size ($d_s$) in the *n*-th iteration shown in Eq. (1). In the dimensions where the value of the function changes more strongly, the mutation is stronger. In the dimensions where the change is slower, the local search dominates.

1 $$\begin{aligned} \textbf{m}_r(n) =\textbf{m}_{r.min}+(\textbf{m}_{r.max}-\textbf{m}_{r.min})\frac{min(\textbf{d}_s(n))}{\textbf{d}_s(n)} \end{aligned}$$

The differential step size was primarily fed back based on the change in the function using Alg. 3 and Eq. (2).

2 $$\begin{aligned} dE(n) = \frac{||\textbf{E}(\textbf{p}(n))|| - ||\textbf{E}(\textbf{p}(n)+\textbf{s}(n))||}{||\textbf{E}(\textbf{p}(n))||} \end{aligned}$$

**Algorithm 3:**
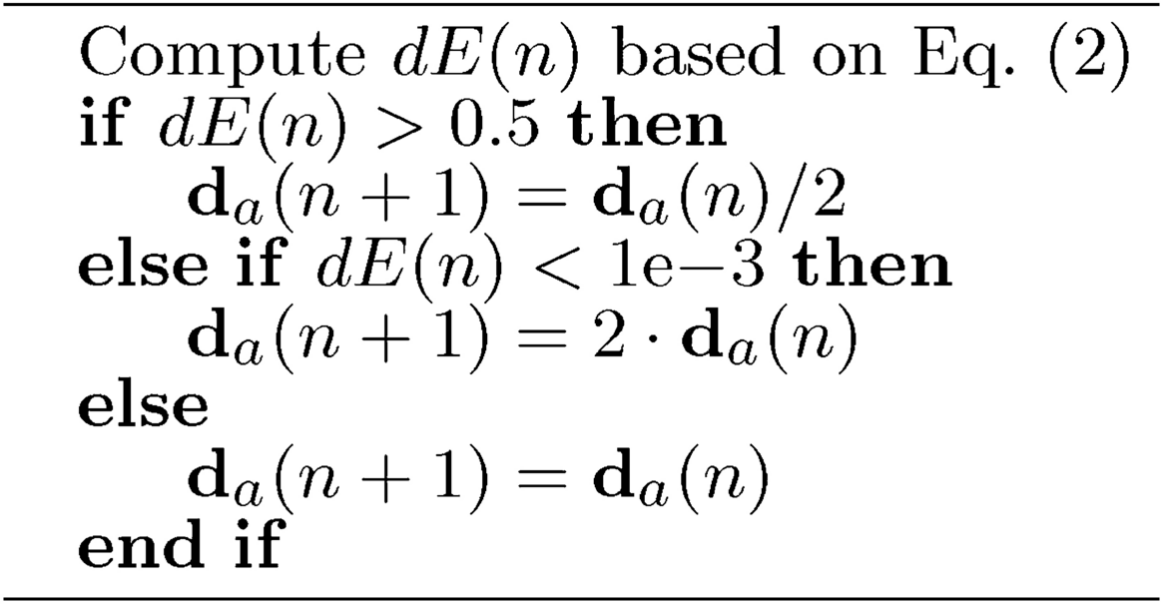
$\textbf{d}_a$ update based on the cost function

 In this paper, the LM algorithm has been applied in a novel way to make efficient use of sub-dimensions. The parameter vector is divided into two parts according to the preselected sub-dimension, the active parameters ($p_a$) and the inactive parameters. The active parameters are updated in each iteration step (*n*). The updating process is described in Alg. 4. The modification vector $s_a(n)$ can be calculated according to Eq. (3).

3 $$\begin{aligned} \textbf{s}_a(n) = -(\textbf{J}_a^T(n)\cdot \textbf{J}_a(n)+\mathbf {\alpha }_a(n) \cdot \textbf{I})^{-1} \cdot \textbf{J}_a^T(n) \cdot \textbf{E}(n), \end{aligned}$$

where $\textbf{J}_a$ is the active part of the Jacobian matrix described in Eq. (4),$\alpha _a$ is the active regularization vector, $\textbf{I}$ is the unit matrix, and $\textbf{E}$ is the error vector with two components described in Section 3.3.

4 $$\begin{aligned} \textbf{J}_a(n) = \frac{\partial \textbf{E}(\textbf{p}(n))}{\partial \textbf{p}_a^T(n)} \end{aligned}$$

The change significantly increases the efficiency of the LM algorithm for higher dimensions since the operation is performed only in the active sub-dimensions. Compared to the original LM algorithm, $\alpha _a$ is not just a parameter but a vector.

**Algorithm 4:**
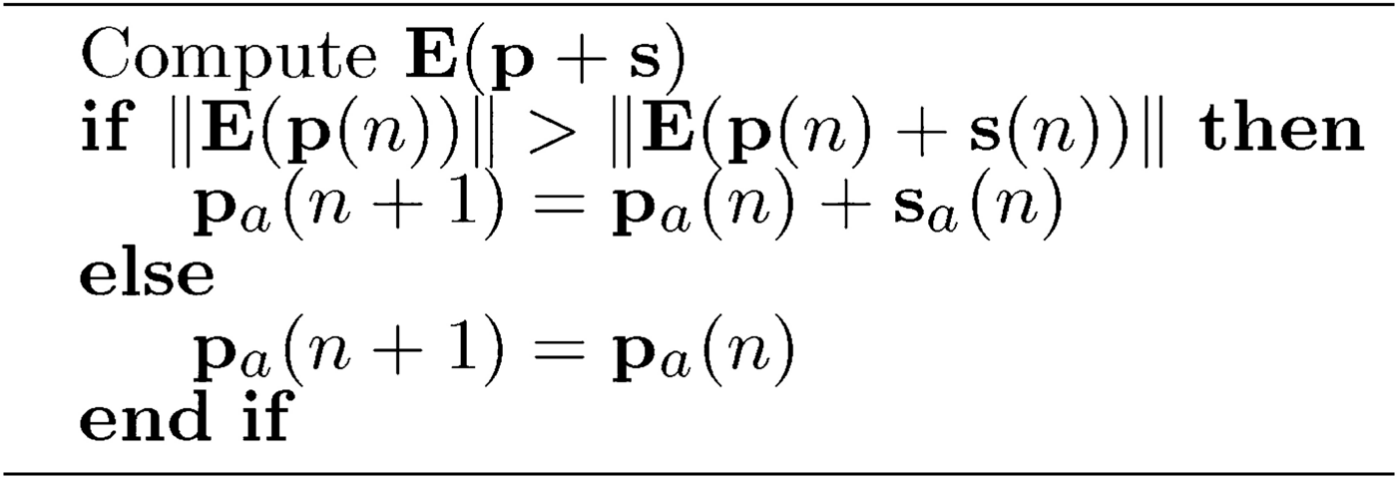
$$\textbf{p}_a$$ update

The regularization vector has been updated based on the trust region in the following simple Alg. 5 based on Eq. (5).

5 $$\begin{aligned} r(n) = \frac{||\textbf{E}(\textbf{p}(n))|| - ||\textbf{E}(\textbf{p}(n)+\textbf{s}(n))||}{||\textbf{E}(\textbf{p}(n))|| - \frac{1}{2}|| \textbf{E}(\textbf{p}(n) -\mathbf {\textbf{J}}_a(n) \cdot \textbf{s}_a(n)||^2} \end{aligned}$$

**Algorithm 5:**
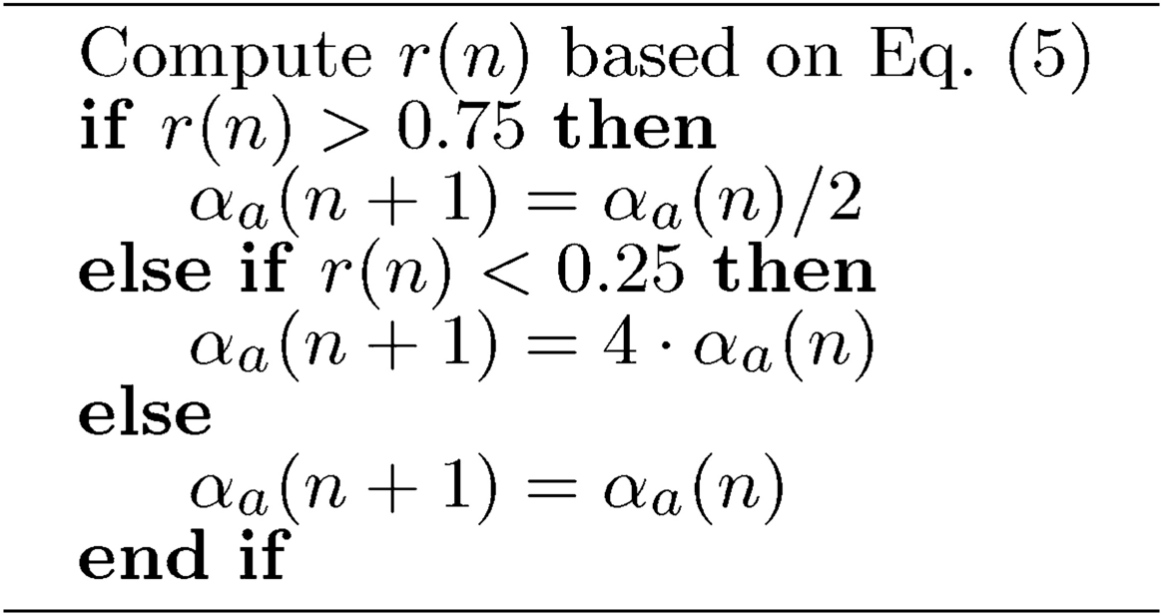
$$\mathbf {\alpha }$$ update

Algorithm 6 was used to align the differential step size and the regularization parameter.

**Algorithm 6:**
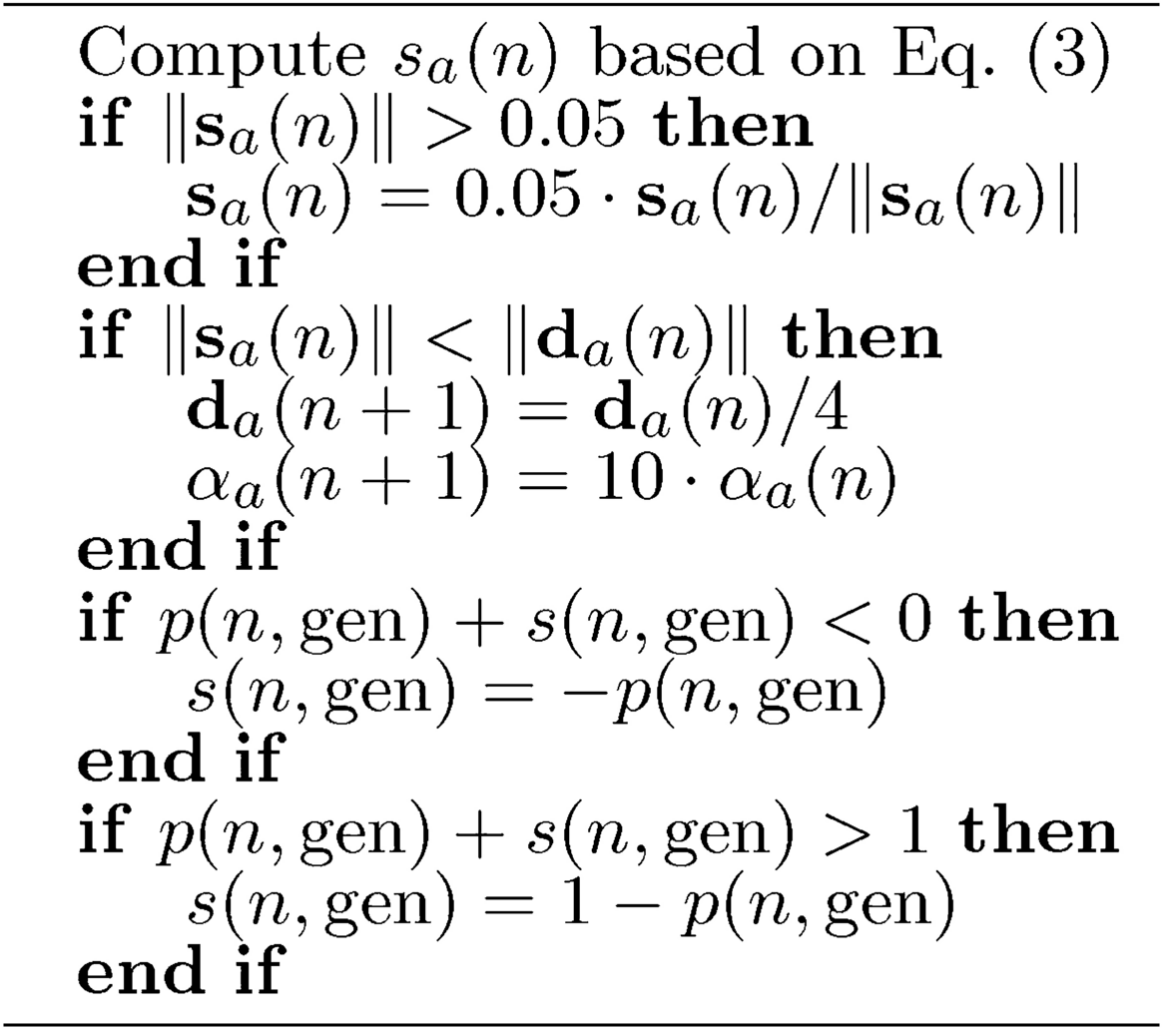
Connection $\textbf{s}_a(n)$, $\mathbf {\alpha }_a(n)$, and $\textbf{d}_a$ update

#### Colony and population management

Colony management is responsible for settling individual colonies and starting new ones. Colonies can be removed in three ways, by merging into other colonies, by depleting territory, or by taking a previous route. When two colonies meet, the one later in ranking is removed. The distance to the meeting can be set as task-dependent or based on accuracy. A colony settles down after multiple unsuccessful searches. If a colony lands on the path of a previous one, then it will be excluded from the search. In all cases, a new colony will be started to replace the old one, randomly for high dimensions and taking into account the previously searched ones for low dimensions. It is important to note that removing and settling down a colony is different. The settled colonies are recorded separately, which gives the Pareto front estimate, together with the final active colonies. With this method, the resolution of the Pareto front is not limited to the size of the active population and can continuously refine as the search continues. The success of the search steps is evaluated in the population management sub-unit. The reduction cost is normalized according to the resource expended (number of simulations). Population management is used to determine the sub-dimensions for clone mutation, local search, and gene transfer. For clone mutation, a GA is used for local search based on previous searches and random for gene transfer. The gene selection process is described by Algorithm 7.

**Algorithm 7:**
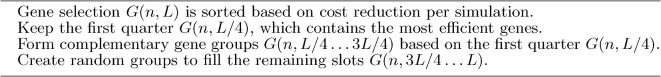
GA for gene selection

### Parameter setting and initialization

A key motivation behind the CBMA design is to reduce the burden of parameter tuning by employing robust self-adaptive mechanisms. Although CBMA can adapt from suboptimal initial parameter settings, providing reasonable values can accelerate convergence significantly. This section outlines the main parameter categories, recommended ranges, and advanced adjustments to optimize performance.

The number of colonies (*M*) defines how many distinct sub-populations are maintained concurrently. A larger *M* is beneficial for highly multimodal functions, complex constraints, or multi-objective scenarios where diversity in search is crucial. Each colony is further divided into *K* collectives, which explore different regions of the solution space. Higher *K* values improve regional exploration based on the adaptive parameter $m_r$. The number of individuals per collective $(N_\textrm{ind})$ governs the depth of local search within each collective. Larger values allow more thorough exploration. Differential step size $(d_s)$ controls the granularity of local searches. Larger values enable rapid traversal of the search space but may skip promising regions, while smaller values ensure fine-grained exploration near optima, albeit at the risk of slower convergence. The number of local searches $(N_\textrm{LS})$ affects the frequency of refinement operations. More frequent local searches enhance exploitation and the refinement of the adaptive local search parameters. Parameters of gene transfer influence how frequently and effectively solutions exchange information. Finally, colonies’ removal or merger threshold ensures that stagnated or redundant colonies are pruned to maintain efficiency. Tighter thresholds favor exploitative searching, while looser thresholds promote exploration by retaining more colonies.

To determine effective parameter settings for general tasks, a random search was conducted using the ranges shown in Table 2 on the CEC-BC-2017 benchmark. Since no single parameter configuration works best for all problems, it is important to use meaningful, well-initialized parameters with strong self-adaptation. The recommended values in Table 3 are rounded values that CBMA’s self-adapted mechanism founded. In general, setting *M* between 8 and 16 is effective, with lower values suitable for simpler tasks and higher values better for multi-objective or complex problems. For *K*, a range of 4 to 10 is recommended, with moderate values balancing problem complexity and runtime. Setting $N_\textrm{ind}$ to around 10 strikes a good balance between computational efficiency and thorough exploitation. The initial regularization value or damping factor ($\alpha$) is recommended to range between 0.01 and 1. The initial differential step size ($d_s$) should range from $1 \times 10^{-3}\%$ to $1 \times 10^{-1}\%$, depending on the parameter’s sensitivity. A mutation range ($m_r$) of approximately 50% often balances exploration and refinement. Similarly, an inheritance probability of around 50% provides a good compromise; higher values reduce reliance on initialization, while lower values increase trust in initial settings. These initial values serve as effective starting points for the self-adaptation mechanism, ensuring flexibility and adaptability across a range of tasks.

**Table 2:** Random parameter ranges used for the CBMA algorithm, including configurations for colonies, collectives, individuals, and adaptive mechanisms. These ranges provide flexibility to explore diverse setups, ensuring robust performance across various tasks.

Parameter	Min.	Max.
Number of colonies	8	16
Number of collectives/colony	2	6
Number of individuals/collective	3	4
Regularization value	0.1	1.00E+06
Differential step size [%]	1.00E−08	1.00E−02
Number of local searches	2	6
Number of clone mutations	2	10
Number of mutated genes [%]	5	50
Mutation range [%]	10	80
Inheritance probability [%]	50	50
Local search random proportion in clone mutation [%]	10	90
Number of local searches in clone mutation	1	4
Number of gene transfers	1	3
Number of transferred genes [%]	15	50
Number of local searches in gene transfer	1	4
Colony remove distance [%]	0.1	0.1
Number of failed local searches before settled	3 $\times$ D	3 $\times$ D

**Table 3:** Summary of adjustable parameters and their proposed values for various optimization algorithms. The parameters listed include those specific to each algorithm’s mechanism, along with references for additional details.

Algorithm	Main parameter	Proposed value
AO	Exploitation adjustment parameter Alpha	0.1
	Exploitation adjustment parameter Delta	0.1
	Other parameters can be found:	^5^
AOA	Alpha sensitive parameter	5
	Control parameter	0.5
	Other parameters can be found:	^41^
DE	Differential weight	0.8
	Crossover probability	0.85
	Other parameters can be found:	^37^
FHO	Maximum number of Fire Hawks	13
	Other parameters can be found:	^40^
JAYA	Parameter free:	^39^
MPA	Fish aggregating devices parameter	0.2
	Other parameters can be found:	^42^
PSO	Personal learning coefficient	1.5
	Global learning coefficient	1.5
	Inertia weight damping ratio	0.99
	Other parameters can be found:	^34^
NRBO	Deciding factor	0.6
	Other parameters can be found:	^26^
DBO	Producers ratio [%]	20
	Deflection coefficient	0.1
	Light intensity adjustment	0.3
	Sensitivity parameter	0.5
	Other parameters can be found:	^3^
CBMA	Number of colonies	16
	Number of collectives/colony	6
	Number of individuals/collective	10
	Regularization value	1E−02
	Differential step size [%]	1E−03
	Number of local searches	4
	Number of clone mutations	6
	Number of mutated genes	10
	Mutation range [%]	50
	Inheritance probability [%]	50
	Local search random proportion in clone mutation [%]	50
	Number of local searches in clone mutation	2
	Number of gene transfers	2
	Transferred genes [%]	20
	Number of local searches in gene transfer	2
	Colony remove distance [%]	0.1
	Number of failed local searches before settled	3 $\times$ D

Self-adaptive mechanisms play a central role in CBMA. Local search parameters, such as damping factors and step sizes, are continuously adjusted based on success metrics, making the algorithm less sensitive to poor initial guesses. Mutation ranges evolve dynamically, with colonies that fail to improve increasing mutation strength to boost exploration, while successful colonies refine their searches by tightening mutation. This adaptability ensures robustness across diverse problem types.

Even with purely random initialization, CBMA rapidly gravitates toward effective parameter regions. However, informed priors can speed up convergence, especially for tasks with known characteristics. Specialized tasks or high-dimensional problems may require additional tuning. For example, increasing *M* can improve diversity in Pareto fronts for multi-objective tasks, while larger $m_r$ values are advantageous for mixed discrete/continuous problems. Reducing $N_\textrm{LS}$ minimizes overhead for costly evaluations, and relaxing colony removal thresholds allows exploration near constrained boundaries.

To maximize CBMA performance, it is advisable to start with moderate values for *M*, *K*, and $N_\textrm{ind}$ and rely on adaptive mechanisms to fine-tune settings. Incorporating known priors, such as typical step sizes or feasible solutions, further accelerates the process. By leveraging its feedback-driven design, CBMA minimizes manual tuning needs, offering a robust and flexible framework for optimization tasks.

### CBMA algorithm complexity

The CBMA algorithm is designed to balance computational and memory complexity by leveraging sub-dimensional computations. Sub-dimensional calculations significantly reduce the algorithm’s computational demands compared to full-dimensional strategies while maintaining individual complexity sufficient for adaptability.

The computational complexity of CBMA is dominated by its sub-dimensional operations, particularly in local search. The most computationally expensive operation is random orthonormal rotations, used in pattern search, with a complexity described in Eq. (6).

6 $$\begin{aligned} \textrm{RotationCost} = \mathcal {O}(S^4) \quad \text {(improvable to } \mathcal {O}(S^3)\text {)}, \end{aligned}$$

where *S* is the size of the sub-dimension. LM updates, another dominant operation, involve matrix inversion in an $S \times S$ subspace, described in Eq. (7).

7 $$\begin{aligned} \textrm{LMCost} = \mathcal {O}(S^3 + C), \end{aligned}$$

where *C* represents the number of cost components. Additionally, distance checks to avoid overlapping collectives, contribute to the overall runtime when performed extensively across large populations. The distance check described in Eq. (8) can be further improved from $\mathcal {O}(D)$ to $\mathcal {O}(S)$, where $N_{memory}$ is the number of saved individuals.

8 $$\begin{aligned} \textrm{DistanceChecks} = N_{memory} \cdot \mathcal {O}(D). \end{aligned}$$

For a population of $N_{ind}$ individuals, $N_{LS}$ local searches, $N_{gg}$ clone mutation cycles, $N_{cinf}$ infection cycles, and the total number of epochs, $N_{epoch}$, the overall computational complexity is formulated in Eq. (9).

9 $$\begin{aligned} \textrm{TC} \approx N_{epoch} \cdot (N_{gg} + N_{cinf}) \cdot N_{ind} \cdot \left( N_{LS} \cdot \left( \mathcal {O}(S^4) + \mathcal {O}(S^3 + C) \right) + N_{memory} \cdot \mathcal {O}(D)\right) . \end{aligned}$$

In practice, this complexity can be minimized by choosing a small *S*, where $S \ll D$, and reducing the frequency of local searches. For $S = D$, the complexity approaches the worst-case scenario of $\mathcal {O}(D^4)$.

Given CBMA’s parallel nature, computational and time complexities differ. In a fully parallel implementation, where operations for $N_{\text {ind}}$ individuals can run concurrently, the time complexity per epoch reduces according to Eq. (10).

10 $$\begin{aligned} \textrm{PC} \approx N_{epoch} \cdot (N_{gg} + N_{cinf}) \cdot \left( N_{LS} \cdot \left( \mathcal {O}(S^4) + \mathcal {O}(S^3 + C) \right) + N_{memory} \cdot \mathcal {O}(D)\right) . \end{aligned}$$

The algorithm’s adaptive parameter settings further influence runtime. CBMA dynamically adjusts parameters such as mutation strengths and step sizes to optimize performance. Although adaptivity reduces sensitivity to initial parameter choices, it can slow convergence. In empirical evaluations, CBMA required approximately $0.5\,\textrm{ms}$ per evaluation, around 20 times slower than simpler methods like GA or PSO. However, for computationally expensive cost functions with evaluation times of $1\,\textrm{s}$, this overhead translates to just one additional evaluation in every 2000 iterations. Optimizations, including improving sub-dimensional rotation algorithms, can further mitigate this runtime gap.

The memory requirement of CBMA depends on storing positional data across *D* dimensions, *C* cost components, and adaptive parameters, such as mutation strengths and success counters. For a population comprising *M* colonies and *K* collectives, the total memory requirement is described in Eq. (11).

11 $$\begin{aligned} \textrm{Memory} = N_{\text {epoch}} \cdot \big (M \cdot K \cdot N_{gg} + N_{cinf} \cdot N_{inf}\big ) \cdot Col, \end{aligned}$$

where $N_{epoch}$ is the total number of epochs, $N_{gg}$ represents the number of gene groups, $N_{cinf}$ the number of infection cycles, $N_{inf}$ the number of parallel infections, and *Col* the space used per colony. For each collective, the additional memory requirement is given by Eq. (12).

12 $$\begin{aligned} \textrm{Col} = 9D + 3C + D \cdot C. \end{aligned}$$

To manage memory usage effectively, practical implementations compress or discard redundant data and limit the number of stored colonies and collectives, ensuring flexibility in memory allocation. During testing, colony storage was limited to maintain comparable memory usage across experiments.

In summary, CBMA achieves a balance between computational efficiency and adaptability through sub-dimensional computations, flexible memory management, and parameter adaptivity. Its memory complexity scales according to Eq. (13).

13 $$\begin{aligned} \textrm{MemoryComplexity} = \mathcal {O}(N_{\text {ind}} \cdot (D + C)), \end{aligned}$$

while computational complexity ranges from near-constant for low-dimensional subspaces to $\mathcal {O}(D^4)$ in the full-dimensional case. By adjusting sub-dimension size, colony storage, and search frequency, users can optimize the algorithm’s performance for various problem dimensions and resource constraints.

## Algorithm summary and algorithm comparison

The presented algorithm is complex and can be divided into several subparts as presented in “Components and innovations of the algorithm”. The individual subparts were created by transforming and harmonizing different known algorithms. One of the main goals of CBMA is to break up a larger problem at both the search space and population levels. The other main goal is to give individuals greater adaptability based on the harmonization of the algorithm’s subparts and local properties of the objective function.

In this section, comparisons and summaries are presented in the same order as in the literature review: adaptivity, evolvability, exploration-exploitation, dimension reduction, constraints handling, and multi-objectivity. One extra subsection is added: the combined algorithms.

### Combined algorithms

The simplest way to combine algorithms is to connect them serial or parallel^84^. The parallel connection’s most significant advantage is a proper comparison, but it requires many individuals. Algorithms are compared at the previous search stage, and there is no guarantee of an optimal algorithm choice for the next step. There is no information sharing between the parallel algorithms. In the case of serial connection, there is no valid comparison between the sub-units of the algorithm, but fewer individuals are sufficient. If comparison is not the goal, information exchange can be used to allow finer control and higher efficiency. Table 4 summarizes the algorithms in terms of connection.

**Table 4:** Parallel and Serial Comparison. There are many options connecting individual modules, in the table we compare the basic serial or parallel connections. Some modules may prefer one over the other. In the case of adaptive modules, a serial connection is preferable.

	Parallel (keep all)	Parallel (select the best)	Serial cooperate
Comparability	Excellent (not used)	Average (no temporality)	Poor (no reference)
Population size	Large (or no comparability)	Large (or no comparability)	Small
Information exchange	Low (or no comparability)	Low (or no comparability)	High
Flexibility	Slight (or no comparability)	Slight (or no comparability)	Strong
Cycle time	Based on the slowest	Based on the momentary slowest	Additive
Power demand	High	Variable	Low
Efficiency	Low	Average	High
Optimization speed	Fast	Fast	Slow

All serial and parallel connections provide many possibilities. Nowadays, adaptive algorithms are typically used. Less critical to choose the best algorithm in adaptive algorithms, and the information is more critical. Connecting algorithms with their corresponding variables lead to better cooperation and higher efficiency. Due to the higher achievable efficiency, the serial connection has been used in CBMA.

### Literature comparison

Multiple adaptation strategies were implemented in CBMA, incorporating both top-down and bottom-up approaches. Information from lower-level adaptation responses was aggregated and then used in a top-down manner. Compared to other state-of-the-art solutions, such as BGADBO^4^, which employ individual-level adaptation, CBMA utilizes uniform descriptions for individuals and stores some adaptation parameters separately for each individual. Instead of external parameters, such as iteration, the variables are fed back are internal parameters, such as cost changes, so that the method can be more robust. Since the algorithm is not bound to external parameters, it can be continued arbitrarily. The algorithm can evolve continuously by restarting, settling, and logging colonies.

Unlike the methods listed in “Literature review”, decisions are made at the individual level rather than the population level. Each individual is placed in exploration or exploitation status based on its overall population and individual performance.

The BEA^31^ based search allows the manual decomposition of the problem into sub-problems and, in combination with the GA, results in automatic dimension selection. BEA has also proven its suitability for NK landscape problems^57^.

In the case of real problems, such as sensitive simulations, it may not always be possible to perform or give an uncertain result in case of evaluation conditions violation. In the CMH-CBMA algorithm, condition violates are recorded separately and replaced with a reserve population. When a reserve population runs out, a new reserve population is initialized.

In order to increase diversity, the omission of individual objectives at the individual level is achieved by individual costs, thus further extending the new idea and complementing it with population clustering. Giving individual goals allows exploring the Pareto front, improves convergence, and increases diversity. Similar to cooperative evolution, but different from it, the targets may not be so different. Changing the targets further improves convergence and helps to explore the Pareto front. As in other multi-objective algorithms, the algorithm includes a resolution of sub-populations (colonies) and their individuals (collectives). The main difference is that here sub-problems are better communicated and interchangeable, resulting in better exploration.

### Comparison on common test functions

This section evaluates the CBMA algorithm against seven modern and two established optimization algorithms: MPA, AO, AOA, FHO, JAYA, DE, PSO, DBO, and NRBO. The optimal or assumed optimal parameters for each algorithm for the CEC-BC-2017^85^ benchmark can be found in Table 3.

The evaluation used the CEC-BC-2017 test functions^85^ with a stopping criterion of 1000 parallel evaluations or 64,000 single evaluations. Single evaluations are a standard metric, but parallel evaluations are increasingly relevant for multi-threaded processes and were used as the primary comparison metric.

**Table 5:** Summary of the test setups for the comparative evaluation of optimization algorithms on the CEC-BC-2017 test function group. The table includes details about the number of tests, iterations, population size, dimensionality, parameter intervals, offset intervals, and rotation intervals used for the experiments.

No. of tests	20
No. of iterations (parallel evaluations)	1000
Population size (parallel evaluation)	64
No. of evaluations	$64\,000$
Dimension (*D*)	[50, 100, 500, 1000]
Parameter interval	$[-100, 100]^D$
Offset interval	$[-100, 100]^D$
Rotation interval	$[-\pi , \pi ]^D$

Table 5 summarizes the experimental setup. Each test was parameterized with unique rotation and shift values, ensuring distinct conditions across trials. Parameters and population configurations were uniformly initialized, providing consistency while preserving test uniqueness. A total of 20 tests were conducted per function group, covering all 38 CEC-BC-2017 functions, resulting in 760 test cases per algorithm.

Iteration interpretation varied. For AOA, DE, PSO, and JAYA, 1000 iterations matched 1000 generations. AO, MPA, and DBO used 500 generations, while FHO, NRBO, and CBMA employed adaptive strategies to adjust generations dynamically. CBMA optimized evaluations by selecting the top 16 individuals from an initial population of 64 as colony centers. This approach enhanced computational efficiency, often completing evaluations before reaching the 64,000 or 1000 iteration limits. CBMA reduced iterations by 25% in lower-dimensional cases and evaluations by 5% in higher-dimensional cases.

Tables 6, 7, and 8 provides a comprehensive comparison of the CBMA algorithm’s performance across different function types, dimensionalities, and algorithms. Each table presents the total cases analyzed, along with the percentage of cases where CBMA performed better, worse, or showed no significant difference compared to other methods. The results are further supported by Cohen’s d-values and Wilcoxon p-values, indicating the effect and statistical significance of the comparisons.

**Table 6:** The table compares CBMA performance across different dimensionalities, showing the total cases, percentage of better, worse, and no significant difference cases. Higher dimensionalities (100D, 500D) show strong CBMA performance in 73% of the time, while lower dimensionalities (1000D, 50D) show more balanced outcomes. The negative Cohen’s d values indicate CBMA’s superior performance, and the p-values confirm statistical significance, especially in higher dimensions.

Dimensionality	Total	Better (%)	Worse (%)	No significant difference (%)	Avg Cohen’s d	Avg Wilcoxon p-value
50	342	69	8	23	− 4.7e+01	9.2e−02
100	342	73	7	19	− 6.1e+01	5.7e−02
500	297	74	19	7	− 7.8e+00	2.3e−02
1000	297	62	30	9	− 4.1e+00	3.0e−02

**Table 7:** The table summarizes CBMA’s performance across different function types, showing the total cases, percentage of better, worse, and no significant difference outcomes. Negative Cohen’s d values indicate CBMA’s advantage, with lower p-values suggesting statistical significance. CBMA performs particularly well in simple multimodal, hybrid, and multimodal functions (over 71% more times) while facing some competition in unimodal and composition functions.

Function type	Total	Better (%)	Worse (%)	No significant difference (%)	Avg Cohen’s d	Avg Wilcoxon p-value
Composition	288	59	25	17	− 1.2e+02	4.7e−02
Hybrid	324	76	13	10	− 4.9e+00	3.3e−02
Multimodal	342	74	6	20	− 7.5e+00	8.1e−02
Simple Multimodal	252	71	15	13	− 6.4e+00	4.9e−02
Unimodal	72	58	31	11	− 2.4e+00	3.3e−02

**Table 8:** The table compares CBMA’s performance against various algorithms, summarizing total cases, and percentages of outcomes where CBMA performed better, worse, or showed no significant difference. CBMA achieved stronger results against algorithms like PSO (46% better) and DE (89% better). Negative Cohen’s d values further validate CBMA’s superior performance, with Wilcoxon p-values supporting statistical significance in the majority of comparisons.

Algorithm	Total	Better (%)	Worse (%)	No significant difference (%)	Avg Cohen’s d	Avg Wilcoxon p-value
AO	142	73	14	13	− 4.6e+00	4.2e−02
AOA	142	83	7	10	− 1.1e+01	3.1e−02
DBO	142	56	20	23	− 2.8e+01	8.5e−02
DE	142	89	8	3	− 1.1e+01	8.4e−03
FHO	142	76	14	10	− 7.0e+01	3.3e−02
JAYA	142	58	22	20	− 7.5e+01	7.0e−02
MPA	142	64	19	17	− 2.2e+00	6.7e−02
NRBO	142	81	13	6	− 8.3e+01	1.9e−02
PSO	142	46	22	32	− 1.2e−01	1.1e−01

We evaluate the performance of the CBMA algorithm across various function types, dimensionalities, and algorithms. Table 7 shows that CBMA consistently performs well in Simple Multimodal, Hybrid, and Multimodal Functions, with over 71% of cases showing better performance, whereas it faces more competition in Unimodal and Composition Functions. Table 6 highlights that CBMA demonstrates strong performance in higher dimensions, particularly at 100D and 500D, with around 73% of cases where CBMA performs better, while lower dimensionalities such as 50D show more instances of no significant difference. Table 8 compares CBMA’s performance against specific algorithms, where PSO, DBO and JAYA are more competitive, while DE, AOA and NRBO are significantly outperformed by CBMA. Overall, the analysis consistently demonstrates CBMA’s superior performance across a variety of problem spaces and algorithms, with statistically significant results indicated by the Wilcoxon p-values. Individual results for each dimension, test functions, and algorithms are available in Supplementary Material.

### Special task comparison

This subsection presents a practical comparison based on a robot arm’s ball-throwing task. In addition to the comparison on the common test function set presented in the previous subsection, the algorithms were compared on a more illustrative task. The ball-throwing is also the introductory example of the real-world application in “Real-life example: darts”. This comparison is a conditional single-objective optimization where the constraints are: reaching the target area in limited time, robot arm constraints, and robot arm acceleration constraints.

**Table 9:** Performance comparison of various optimization algorithms (AO, AOA, DE, MPA, PSO, FHO, JAYA, DBO, NRBO, and CBMA) based on iterations, evaluations, and success rates. The table presents the mean number of iterations and evaluations (with 95% confidence intervals), along with their minimum and maximum values. Success rates (%) indicate the proportion of successful runs for each algorithm. CBMA and MPA demonstrate the highest success rate (100%) with relatively low iterations and evaluations, while other algorithms, such as AO and AOA, show lower success and require significantly more evaluations and iterations.

Algorithm	Iterations mean (95% CI)	Min iterations	Max iterations	Evaluations mean (95% CI)	Min evaluations	Max evaluations	Success (%)
CBMA	135 (4)	14	577	6073 (224)	917	30,179	100
AO	1000 (2)	1	1001	56,329 (998)	64	80,080	0.6
AOA	969 (9)	1	1001	54,424 (1073)	64	80,080	5.9
DE	758 (17)	1	1001	42,918 (1292)	64	80,080	57.9
MPA	632 (6)	1	714	35,480 (697)	64	55,120	100
PSO	1000 (0)	1	1001	56,336 (999)	64	80,080	0.1
FHO	981 (7)	1	1001	55,080 (1054)	64	80,080	2.9
JAYA	843 (17)	1	1001	47,359 (1302)	64	80,080	30
DBO	51 (3)	1	1001	2728 (94)	64	32,000	99
NRBO	955 (8)	1	1001	54,307 (1105)	64	80,000	19

The example presented in “Real-life example: darts” is a conditional hierarchical multi-objective optimization. Solving it as a single objective is inefficient. Nevertheless, the algorithm must be competitive on single-objective tasks. The algorithms are compared with their recommended parameters using an illustrative example. For the CBMA, random initial parameters are used since one of the key features of the algorithm is its adaptability. In contrast to the previous example, the optimization does not bound but runs to the hit (maximum 1000 iterations). The comparison is based on the number of parallel and single evaluations, so the population size is initialized freely between 32 and 80 but uniformly for each of the 1000 tests. In the case of CBMA, the colony centers are randomly selected from the initial population. The parameters for CBMA were randomly initialized near the recommended values, as detailed in Table 2. Table 9 shows the number of single and parallel evaluations required to hit the target. Both CBMA and MPA had a 100% successful rate, but CBMA required 79% less iteration and 83% less evaluation. DBO required even fewer iterations and evaluations on average, but did not achieve 100% success.

**Fig. 11:**
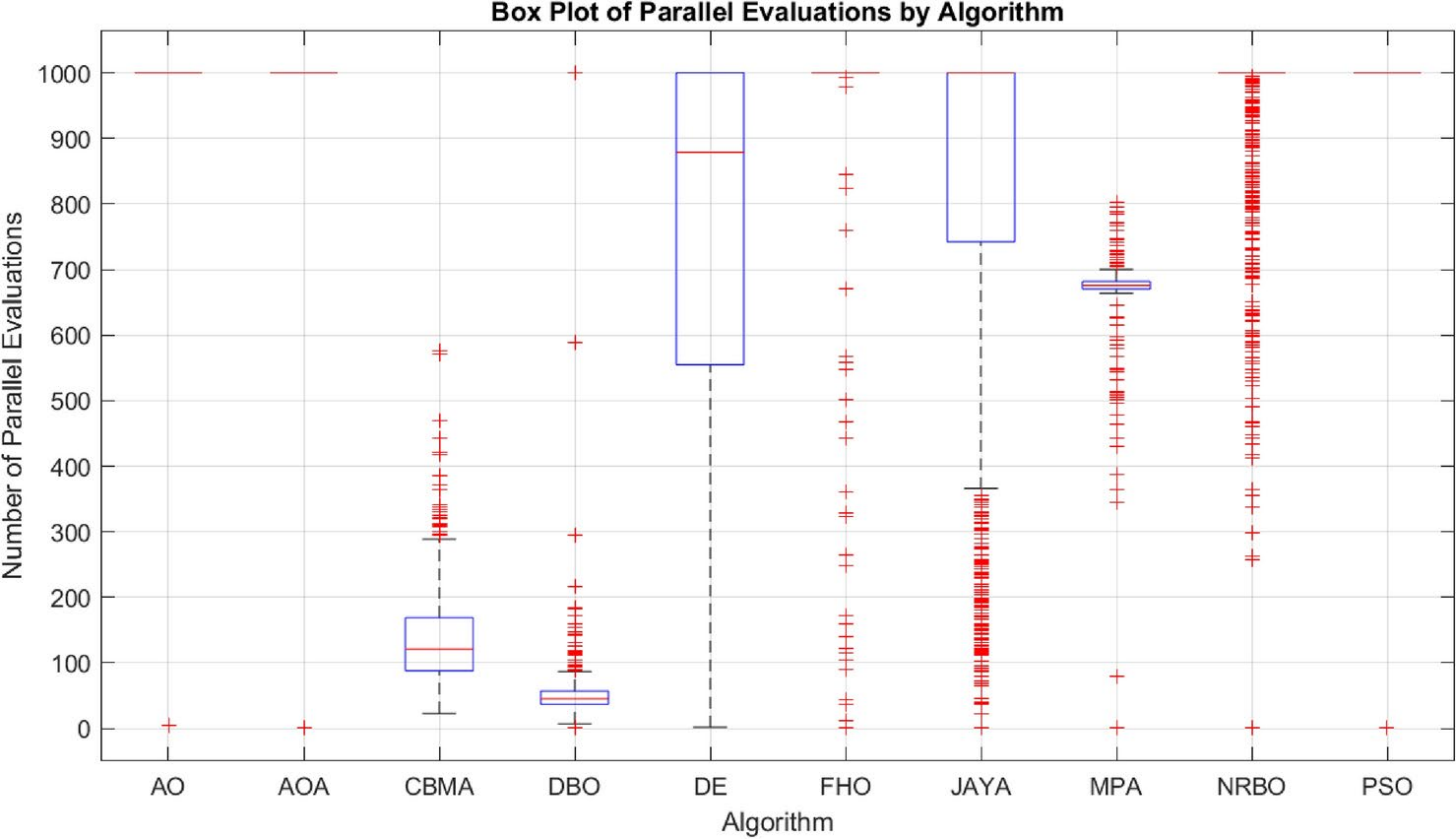
The distribution of the required number of parallel evaluations until hitting the desired region with the ball (maximum: 1000).

**Fig. 12:**
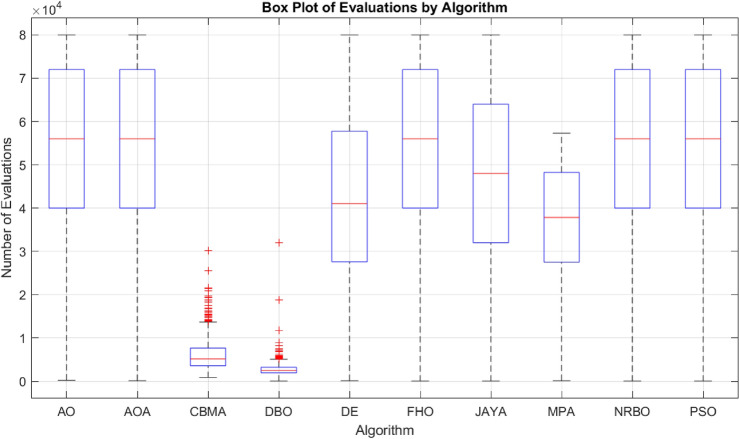
The distribution of the required number of evaluations until hitting the desired region with the ball (maximum: $1000\times Population$).

Figures 11 and 12 show the iteration and parallel evaluations required to complete the task. The distributions illustrate well the stability and performance of the algorithms.

### Comparison summary

The comparison of algorithms highlights the distinct advantages and trade-offs inherent to different optimization strategies. Serial and parallel connections offer flexibility in algorithm design, with parallel approaches excelling in comparison capability and serial connections prioritizing efficiency and adaptability.

On the CEC-BC-2017 benchmark, CBMA consistently outperformed other algorithms. Its performance was particularly strong in Simple Multimodal, Hybrid, and Multimodal functions, achieving success rates above 70%. In contrast, Composition and Unimodal functions presented more balanced outcomes, with CBMA showing competitive results but less dominance. Statistically, CBMA’s advantage was supported by negative Cohen’s d values and low Wilcoxon p-values, confirming its reliability in complex problem spaces.

CBMA’s computational efficiency further solidified its position. It completed lower-dimensional problems with 25% fewer iterations and required 5% fewer evaluations in higher-dimensional cases compared to other algorithms. For practical tasks, such as the robot arm ball-throwing example, CBMA demonstrated exceptional efficiency and stability, averaging 135 iterations and 6073 evaluations while maintaining a 100% success rate.

These results underscore the importance of adaptivity and information exchange in achieving optimal performance. CBMA’s integration of colony-based strategies and dynamic adjustment mechanisms demonstrates its potential as a robust tool for solving both multi-objective and single-objective optimization problems across various domains.

## Real-life example: darts

**Fig. 13:**
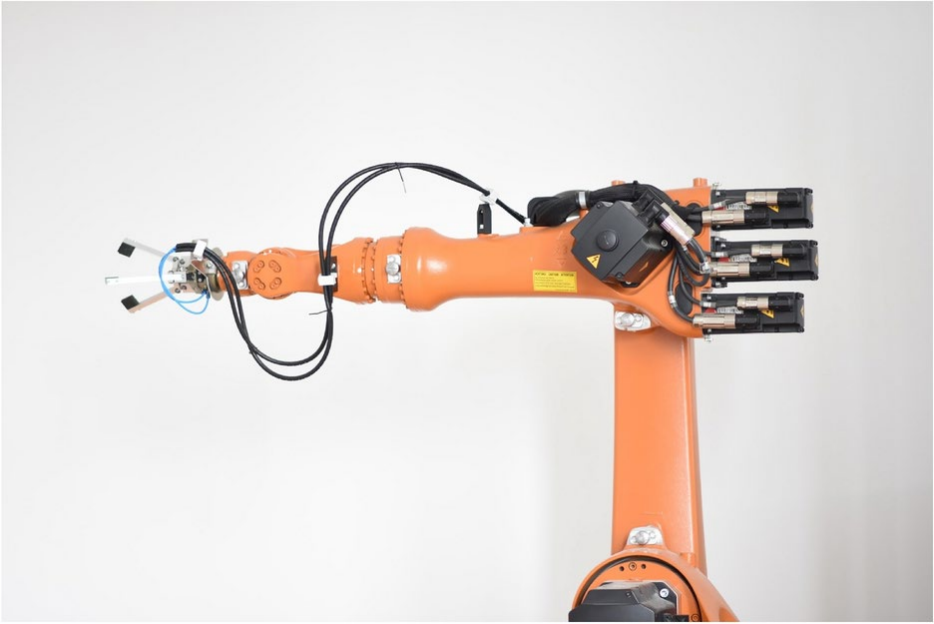
The experimental robot setup features an industrial robotic arm with a precision gripper, optimized using CBMA for trajectory planning and darts-playing accuracy.

This section presents a real-life example of a robot playing darts. The robot’s goal is to hit each region of the dartboard with the highest probability. Based on the previous ball-throwing comparison only CBMA was used for optimization. The experimental robot setup is shown in Fig. 13. The identification of the dart and the estimation of the robot’s accuracy were based on camera records.

The section is structured as follows: The first subsection presents the justification of choice and difficulties of the task. “Task related literature” contains the related literature. In “Proposed solution”, the modeling-based solution is described. In the last “Results’ presentation”, the results are presented in detail.

Darts is a well-known game, yet it is a complex problem that provides an excellent example of conditional partially hierarchical multi-objective optimization. The educational relevance should not be neglected, as a well-known problem helps to facilitate understanding. Last but not least, the real problem can be paralleled with other specific real engineering problems.

**Fig. 14:**
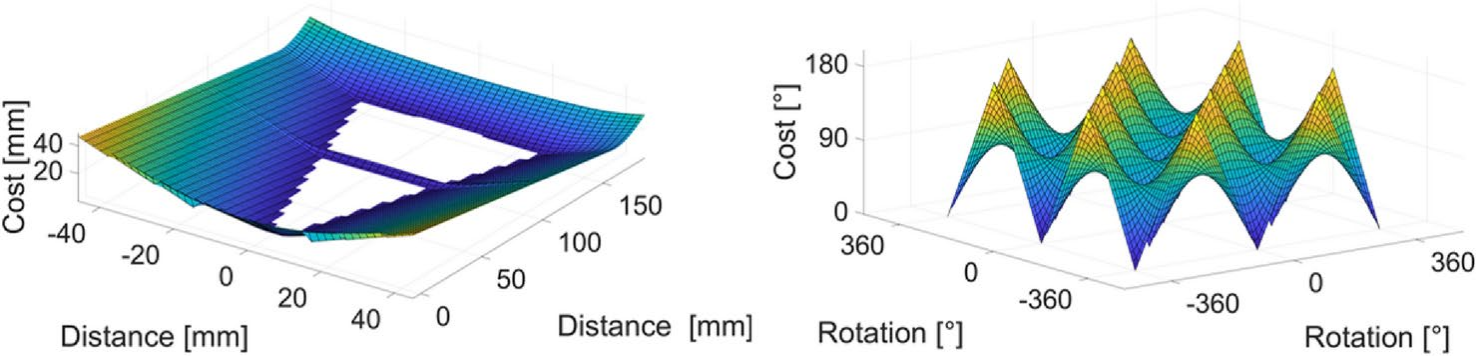
(**a**) The cost function’s first component: the distance from the selected region. As an example, region 20 has been selected, where the exact region has been highlighted in white. (**b**) Cost function’s second component: the arrival angle as a function of the rotations.

The task constraints are as follows: finite acceleration of the robot arm, arrival time $<1 s$, the dart cannot move along arbitrary paths (limitations of the room size), and the arrival angle $<40^{\circ }$. Presentation of the objectives: the dartboard has 62 regions of nearly equal importance. The arrival location has priority over the angle of arrival if the angle is small (soft limit); otherwise, the angle of arrival has priority over the location. Figure 14 shows an example of the cost components of the arrival position and angle.

### Task related literature

The literature review will focus on darts and optimizations for robots. First, statistics related to darts are presented to help the modeling. Then, solutions to similar problems with robots are summarized.

No known initialization parameters exist for robot darts, but several statistical comparisons have been made for humans. For a similar setup, only human data are available for comparison. For humans, the optimal release of the arrows is at a speed of 5.8–6.7 m/s on a circular track with a radius of 0.5–0.7 m. To be expressed in time, 4–25 ms before the peak of the circular movement of the hand. The optimal throwing strategy is throwing with the diameter with an optimal release angle of $17--37^\circ$ before the lever becomes vertical and a suitable optimum speed of 5.1–5.5 m/s. The arrow is released 35–44 ms before the hand movement reaches its peak to be expressed in time. When calculating the optimal accuracy ratio, it was found that throws from the upper arm were 7–20% more accurate than throws from the best forearm. It was observed that throwing with a radius of curvature of 0.8 m and a speed of 5.5 m/s from the upper arm was the most accurate^86^. The work of James and Potts, who investigated the movement of darts using high-speed video technique, has been of great help in terms of darts modeling. A three degrees-of-freedom (DoF) parametric model was created and compared with the measured data^87^.

Darts game is an obvious way to test new methods, so there are some examples in the literature. Lawrence et al. presented a new gradient estimation procedure and tested it on the darts throwing task. Testing was performed in a simulation environment with additional measurement noise. The experiment aimed to tune a PD controller to hit the center of the dartsboard^88^. Kober et al. focused on meta-parameter learning in robotic applications. It is essential for similar tasks not to completely re-learn the solution. Two examples showed that the effective use of meta-parameters can significantly speed up the learning of similar problems. The robot was taught Around the Clock darts game and ball strike in table tennis^89^. Akbulut et al. proposed an Adaptive Conditional Neural Movement Primitives framework for efficient policy improvement in novel environments and effective skill transfer between agents. Their framework is based on the Conditional Neural Process, effectively combining supervised and reinforcement learning methods. Both real and simulated robot trajectory planning tasks were shown adaptation to novel situations based on the previously learned problems^90^. Freek Stulp and Gennaro Raiola implemented dynamic movement primitives learning in Pepper humanoid robot^91^. Pepper played “Ball in a Cup” and only the Velcro darts game for safety reasons. Initially, a human guides Pepper’s arm to teach it how to throw. Vidaković et al. proposed a methodology for task-oriented learning. First, demonstration-based learning using camera classification, then a task-oriented black-box policy search algorithm to optimize the solution. Dynamic Movement Primitives were utilized in trajectory policies. Covariance Matrix Adaptation Evolution Strategy was used as the search algorithm^92^.

### Proposed solution

The optimization was done in the virtual environment. Optimization in a virtual environment is cost-efficient, safe, flexible, and can be faster. One of the most crucial properties of a simulation is the resolution. Many techniques exist to create an adaptive resolution, but the more realistic the simulation is, the more required computation capacity. The best practice is to increase the simulation accuracy along the optimization process. For real-world problems, modeling and simulation are resource-intensive tasks, so modeling and optimization must work together. Co-evolution is one of the foundations of coexistence. There are three possible ways to evolve the model: increasing the spatial and temporal domain, increasing the spatial and temporal resolution, and increasing the detail of the description of the phenomenon. The first two methods were implemented in the identification of the darts model. Small temporal resolution can lead to instability. The ball model presented in “Special task comparison” has been used as a simplified pre-simulation model in this paper. The evolving model has great significance but distorts the value of the objective functions. It is possible to address the bias by repeating the simulation, introducing weighting, or the stepped cost design^93^. A major advantage of the stepped cost design is that it can easily handle individuals at multiple levels of development in a population. Despite the massive simulation, real-world experience is needed in most cases.

A model of the robot and the darts were created in a simulation environment. Datasheets, measurement data, and cameras were used to specify both models. The modeling is described in more detail in the Supplementary Material. The robot model uses the following parameters: the arm’s length, the robot’s Denavit-Hartenberg implementation, each joint’s motion range, maximum speeds, and accelerations. Trapezoidal velocity functions approximated the robot’s dynamics in the absence of further data. Delay, ramp-up, overshoot, and various constraints were observed from the measurement data.

The dart model used the following parameters: projection images, mass, inertia matrix estimation, center of gravity estimation, and air resistance coefficient estimates for the dart parts. Parameters that were difficult to determine, such as the inertia matrix, the center of gravity, and drag coefficients, were identified from the estimated/modeled values. The applied dart model extends James’s and Potts’s model^87^ with continuous integration and three more degrees of freedom. The simulation environment in MATLAB Simulink^94^ was created by merging and placing the submodels. Throwing parameters are based on human-like throwing data research by James and Potts extended to robot dimensions^87^. In the case of a robot, the throw can start from a distance of about 3 m, at 5...7 m/s initial velocity and $-10^\circ ...30^\circ$ initial direction, $-900...200 1^\circ$/s initial angular velocity, and $\pm 90^\circ$ initial angle.

The parameters of the optimization are the angular positions of the robot joints and their angular velocity, a total of 12 parameters.

**Fig. 15:**
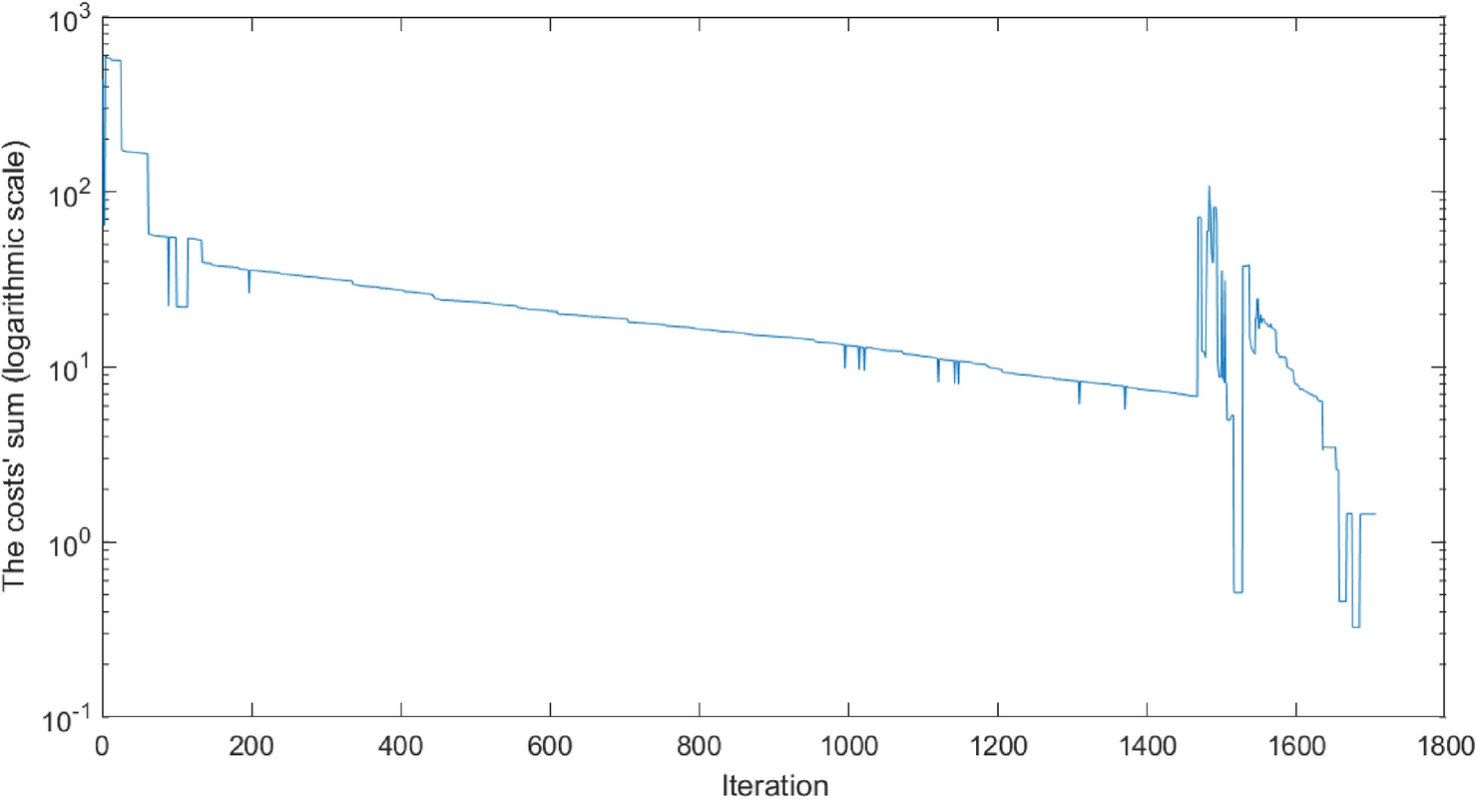
The figure presents the sum of costs on a logarithmic scale for the best-performing individual throughout the optimization process. Spikes on the plot indicate changes in cost composition and target adjustments, emphasizing the dynamic adaptation during optimization. The transition from Stage 3 to Stage 4 is distinctly visible around the 1350th iteration, marked by a more stable shift in costs. In the final hundred iterations, the target is fully mapped, reflecting the completion of the dartboard optimization process..

The optimization process was organized into four stages, as summarized in Table 10. Progress through each stage depended on meeting a predefined cost function criterion. Due to the realistic nature of the task, both the model and the Monte Carlo-based probability distribution were iteratively updated, reflecting evolving costs over time. Figure 15 illustrates this progression, showing visually distinct transitions between the final stages. Although data from the first two stages were not recorded to decrease the memory consumption, the transition from Stage 2 to Stage 3 is evident from a noticeable spike during the first saved iteration.

**Table 10:** Outline of the key operations performed during each phase of the optimization process. The progression involves transitioning from a simple model to a dynamic model, incorporating distributions, pre-simulation adjustments, and enabling gene transfer in the final phase to refine and enhance the optimization outcomes.

Operation	Stage 1	Stage 2	Stage 3	Stage 4
Simple model	X	X		
Dynamic model			X	X
Distribution		X		X
Pre-simulation			X	X
Gene transfer				X

In the initial two stages, a simpler “ball model” was employed. During Stage 1, this model was used for pre-simulation with zero variance; in Stage 2, variance was gradually introduced. After Stage 2, the model was updated to a more dynamic “dart model,” necessitating a recalculation of costs and providing a more detailed representation. .

To accelerate optimization, a uniform distribution was not applied during Stages 1 and 3; instead, only one sample point was used. Transitioning from individual throws to distribution also required cost recalculations. In the early stages, individuals were restricted from communicating, which promoted diverse exploration of different throwing styles, explored in Table 11. Gene transfer operations were likewise disabled in the first three stages to prioritize identifying potential throwing styles before introducing further complexity.

The fourth stage refined the Monte Carlo distribution substantially, increasing both the sampling density, which again led to cost recalculations. This focused approach helped reduce biases from individual sampling and strengthened the overall optimization process.

### Results’ presentation

**Fig. 16:**
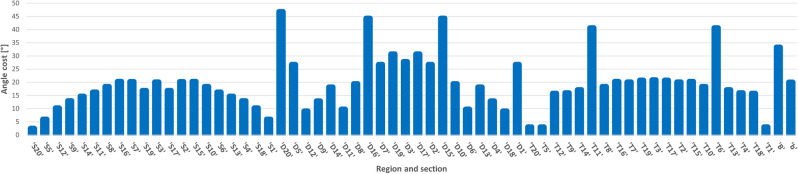
Arrival angles for each section. Abbreviations: “B”: Inner Bull; “b”: Outer Bull; “s”: Inner Single; “S”: Outer Single; “D”: Double; “T”: Treble. One of the interesting things in the figure is that the robot can hit higher regions at a more optimal angle of incidence. With this, we get a kind of confirmation of the distances developed over time in connection with darts, that they seem optimal for the average human height.

The optimization exploits a symmetric layout to explore 35 regions instead of 62. As previously expected, the optimal hit positions were higher for a larger robot arm compared to a human. Figure 16 shows that it can hit the higher-altitude regions of the board at a lower angular position. Table 11 shows that the lack of information transfer resulted in throws from different positions and speeds. The results show that the exploration of different possible throwing styles was successful. In the case without variance, they all represent a global and optimal environment.

**Table 11:** The Table shows the success of the exploration phase. The movement and speed range of each joint is divided into 10 equal parts, showing the successful throws in that range. There are some solutions in almost all of the ranges, which suggests that the optimization found many different throwing styles.

Sections [%]	0–10	10–20	20–30	30–40	40–50	50–60	60–70	70–80	80–90	90–100
Joint 1 pos.	3	20	5	0	16	1	2	0	0	15
Joint 2 pos.	0	13	0	3	3	16	22	2	1	2
Joint 3 pos.	0	1	11	1	24	2	0	12	5	6
Joint 4 pos.	22	3	2	5	12	1	1	11	3	2
Joint 5 pos.	1	22	10	5	2	12	1	5	2	2
Joint 6 pos.	0	4	0	12	5	0	25	1	1	14
Joint 1 vel.	2	1	1	1	1	20	4	0	14	18
Joint 2 vel.	4	17	23	4	9	1	0	0	0	4
Joint 3 vel.	22	1	3	1	1	13	8	1	3	9
Joint 4 vel.	23	3	30	0	1	1	0	0	4	0
Joint 5 vel.	2	22	2	4	0	19	3	4	5	1
Joint 6 vel.	10	6	1	0	0	1	4	5	25	10

**Fig. 17:**
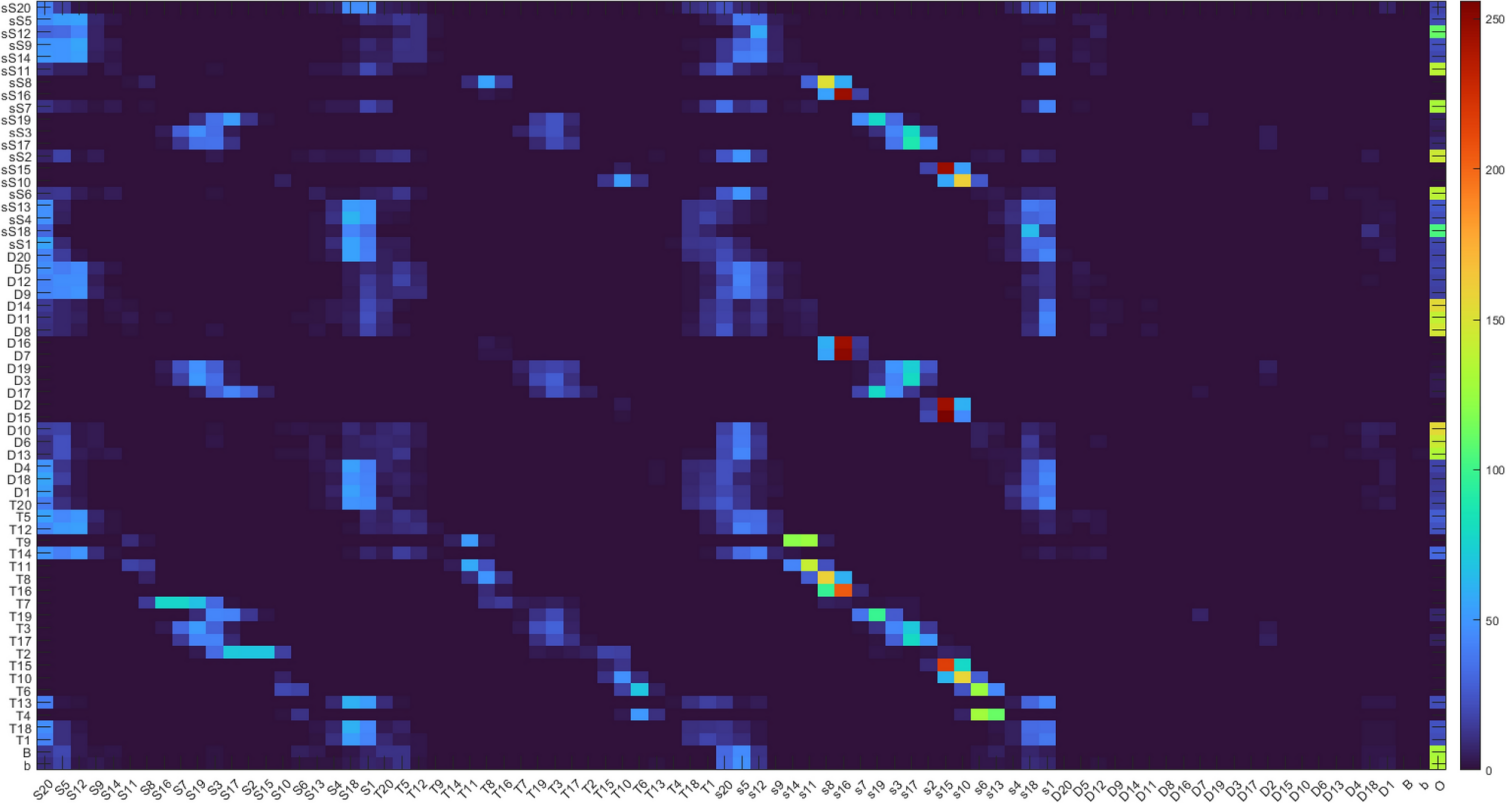
Representation of hit distribution normalized to 8 bits. Each row shows the region wanted to be hit, and the columns show the probability of hitting an area according to the color scale.

**Fig. 18:**
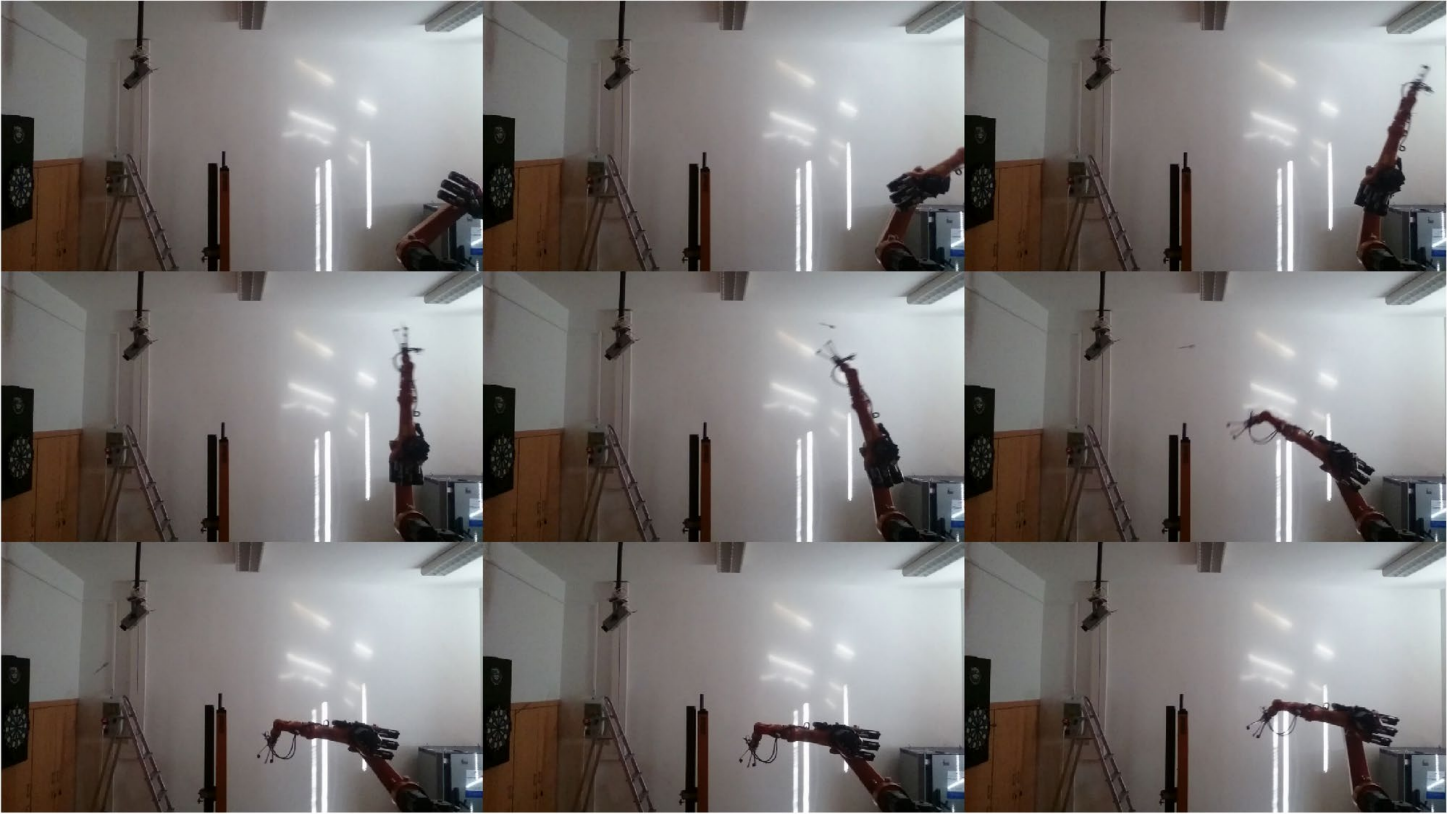
This montage shows a test throw on a real robot and layout.

Figure 17 shows the hit distribution as a function of the targeted region. The banded arrangement is observable, so areas closer to the target region are more likely to be hit than areas further away.

Figure 18 shows a montage of a throw in the real world. The robot’s controller cannot set exact speeds, so precise positioning is not guaranteed, but the robot can throw well. As discussed in “Proposed solution”, further real optimization can be done to improve the accuracy by exploiting the good repeatability of the robot.

## Conclusion

This study presented the Colonial Bacterial Memetic Algorithm (CBMA), an advanced evolutionary optimization method that integrates multiple optimization techniques, and sophisticated adaptive mechanisms, including dynamic gene selection, hierarchical population clustering, and co-evolutionary processes, to effectively address complex optimization challenges. CBMA demonstrates superior adaptability, efficiency, and scalability across a wide range of optimization tasks, as validated by rigorous benchmarking and practical real-world application.

### Benchmark performance

Comprehensive evaluations using the CEC-BC-2017 benchmark suite demonstrated that CBMA consistently outperformed state-of-the-art optimization algorithms, achieving superior outcomes in 71% of high-dimensional cases (100D and 500D). This performance was particularly notable for complex function types, such as hybrid and multimodal functions, where CBMA achieved statistically significant improvements. Specifically, CBMA showed average performance gains of 22% over DE and 18% over PSO. Statistically significant improvements in key metrics, such as Cohen’s d values (ranging from -50 to 0) and Wilcoxon p-values ($p < 0.01$), further underscore CBMA’s effectiveness. The algorithm’s adaptive parameter tuning allows efficient handling of task-specific parameters, optimizing solution quality while minimizing resource consumption, thereby supporting a balanced approach to exploration and exploitation. Furthermore, multi-objective optimization expands upon single-objective functions. Metrics originating from multiple single-objective targets to assess both the quality of the solution and the uniformity of the spread of solutions. Failure in single-objective tasks generally indicates poor performance in multi-objective scenarios.

### Practical applications

The practical utility of CBMA was illustrated through its application to a robot arm’s ball-throwing task, where it achieved a 100% success rate in reaching the target with significantly fewer iterations and evaluations compared to other methods. These results highlight CBMA’s ability to solve constraint-bound, single-objective tasks with stringent performance requirements, showcasing its effectiveness in both sequential and parallel evaluation scenarios. Further demonstrating CBMA’s real-world applicability, the algorithm was used in a darts-throwing task by a robot arm, successfully optimizing throws across multiple regions of the dartboard. The algorithm identified diverse and efficient throwing strategies, demonstrating its adaptability and robustness in managing complex dynamics. These results confirm CBMA’s potential in addressing real-world problems involving uncertainties and dynamic conditions.

### Limitations and future work

While CBMA demonstrates significant strengths, its primary limitation is computational time, which, though noteworthy, is still insignificant compared to the time required for advanced simulations in real-world settings. In conclusion, CBMA represents a substantial advancement in adaptive EAs. Its hierarchical and dynamically adaptive design makes it a powerful and versatile tool for tackling high-dimensional and multi-objective optimization problems in both research and industrial domains. Future work will focus on enhancing CBMA’s parallel processing capabilities, refining local search methodologies, and further improving its adaptive features to broaden its applicability and efficiency in solving complex optimization challenges.

## Supplementary Information

Supplementary Information.

## Data Availability

The data supporting the findings of this study are available on GitLab at https://gitlab.inf.elte.hu/CBMA_Darts_Playing_Robot.

## Abbreviations

AO: Aquila optimizer
AOA: Arithmetic optimization algorithm
BEA: Bacterial evolutionary algorithm
BMA: Bacterial memetic algorithm
CBMA: Colonial bacterial memetic algorithm
CMH-CBMA: Constrained multi-hierarchical—colonial bacterial memetic algorithm
DBO: Dung beetle optimizer
BGADBO: Multi-strategy dung beetle optimizer
DE: Differential evolution
EA: Evolutionary algorithm
FHO: Fire Hawk optimizer
GA: Genetic algorithm
GWO: Grey wolf optimizer
LM: Levenberg-Marquardt
MPA: Marine predators algorithm
NRBO: Newton-Raphson-based optimizer
PSO: Particle swarm optimization
SPSA: Simultaneous perturbation stochastic approximation
VMWSA: Variable multi-weighted sum approach
WSA: Weighted-sum approach
