# Supplementary material for: Colonial bacterial memetic algorithm and its application on a darts playing robot
Source: Sci Rep. 2025 Mar 28;15:10757. doi: 10.1038/s41598-025-94245-1 (PMC11953357; doi:10.1038/s41598-025-94245-1)
Supplement: Supplementary file 1 — Supplementary Information. [file 41598_2025_94245_MOESM1_ESM.pdf]

Supplementary Material

Szilárd Kovács[1*], Csaba Budai[2] and János Botzheim[1]

[1*]Department of Artificial Intelligence, Faculty of Informatics, Eötvös Loránd University, Pázmány P. sétány 1/A, Budapest, 1117, Pest, Hungary.

[2]Department of Mechatronics, Optics and Mechanical Engineering Informatics, Faculty of Mechanical Engineering, Budapest University of Technology and Economics, 4-6 Bertalan Lajos Street, Budapest, 1111, Pest, Hungary.

*Corresponding author(s). E-mail(s): kovacsszilard@inf.elte.hu;
Contributing authors: budaicsaba@mogi.bme.hu; botzheim@inf.elte.hu;

S1 Introduction

The supplementary material accompanying the article Colonial Bacterial Memetic Algorithm and its Application on a Darts Playing Robot consists of two main components that provide additional details and support for the research findings.

Section S2 presents a comprehensive evaluation of the CEC-BC-2017 benchmark functions, offering a detailed comparative analysis of the CBMA algorithm against other state-of-the-art optimization techniques. The performance of CBMA is assessed across multiple test functions and dimensional settings. Statistical analyses, including mean performance metrics, standard deviations, and significance tests, are provided in tabular format to facilitate a rigorous validation of the results.

Section S3 describes the robotic simulation environment used in the study. It includes information on the simulation framework, the adaptations made to apply CBMA to robotic tasks, and the constraints imposed by the robotic platform. Details regarding the simulation parameters, motion modeling, and performance evaluation metrics are provided to ensure a thorough understanding of the applied methodologies.

S2 Comparison Test on Functions

The algorithms are compared on the test function group CEC-BC- 2017 [S1]. The test functions contained in the set are presented in Table S1, Table S2, and Table S3.

Supplementary Table S1 CEC 2017 Test function set I

	No.	Function
**Unimodal Functions**	F1	Bent Cigar Function
	F2	Zakharov Function
**Simple Multimodal Functions**	F3	Rosenbrock's Function
	F4	Rastrigin's Function
	F5	Expanded Schaffer's F6 Function
	F6	Lunacek bi-Rastrigin Function
	F7	Non-continuous Rastrigin's Function
	F8	Levy Function
	F9	Modified Schwefel's Function
**Multimodal Functions**	F10	High Conditioned Elliptic Function
	F11	Discus Function
	F12	Ackley's Function
	F13	Weierstrass Function
	F14	Griewank's Function
	F15	Katsuura Function
	F16	HappyCat Function
	F17	HGBat Function
	F18	Expanded Griewank's plus Rosenbrock's Function
	F19	Schaffer's F7 Function

Supplementary Table S2 CEC 2017 Test function set II

	No.	Function
**Hybrid Function**	F20	Hybrid Function 1: Zakharov Function, Rosenbrock Function, Rastrigin's Function
	F21	Hybrid Function 2: High Conditioned Elliptic Function, Modified Schwefel's Function, Bent Cigar Function
	F22	Hybrid Function 3: Bent Cigar Function, Rosenbrock Function, Lunache Bi-Rastrigin Function
	F23	Hybrid Function 4: High Conditioned Elliptic Function, Ackley's Function, Schaffer's F7 Function, Rastrigin's Function
	F24	Hybrid Function 5: Bent Cigar Function, HGBat Function, Rastrigin's Function, Rosenbrock's Function
	F25	Hybrid Function 6: Expanded Schaffer F6 Function, HGBat Function, Rosenbrock's Function, Modified Schwefel's Function
	F26	Hybrid Function 7: Katsuura Function, Ackley's Function, Expanded Griewank's plus Rosenbrock's Function, Modified Schwefel's Function, Rastrigin's Function
	F27	Hybrid Function 8: High Conditioned Elliptic Function,Ackley's Function, Rastrigin's Function, HGBat Function, Discus Function
	F28	Hybrid Function 9: Bent Cigar Function, Rastrigin's Function, Expanded Griewank's plus Rosenbrock's Function, Weierstrass Function, Expanded Schaffer's F6 Function
	F29	Hybrid Function 10: Happycat Function, Katsuura Function, Ackley's Function, Rastrigin's Function, Modified Schwefel's Function, Schaffer's F7 Function

Supplementary Table S3 CEC 2017 Test function set III

	No.	Function
**Composition Functions**	F30	Composition Function 1: Rastrigin's Function, Griewank's Function, Modifed Schwefel's Function
	F31	Composition Function 2: Rosenbrock's Function, Ackley's Function, Modified Schwefel's Function, Rastrigin's Function
	F32	Composition Function 3: Rosenbrock's Function, Ackley's Function, Modified Schwefel's Function, Rastrigin's Function
	F33	Composition Function 4: Rastrigin's Function, Happycat Function, Ackley Function, Discus Function, Rosenbrock's Function
	F34	Composition Function 5: Expanded Scaffer's F6 Function, Modified Schwefel's Function, Griewank's Function, Rosenbrock's Function, Rastrigin's Function
	F35	Composition Function 6: HGBat Function, Rastrigin's Function, Modified Schwefel's Function, Bent-Cigar Function, High Conditioned Elliptic Function, Expanded Scaffer's F6 Function
	F36	Composition Function 7: Ackley's Function, Griewank's Function, Discus Function, Rosenbrock's Function, HappyCat Function, Expanded Scaffer's F6 Function
	F37	Composition Function 8: Hybrid Function 5, Hybrid Function 6, Hybrid Function 7
	F38	Composition Function 9: Hybrid Function 5, Hybrid Function 8, Hybrid Function 9

S2.1 50D

Supplementary Table S4 Comparison with Unimodal Functions 50 dimension

Function	Metrics	AO	AOA	DE	MPA	PSO	FHO	JAYA	NRBO	DBO	CBMA
F1	mean	7.1E+05	1.2E+11	1.9E+10	1.2E+09	5.2E+07	2.3E+10	6.5E+07	3.4E+10	2.4E+08	**8.5E+04**
F1	std	4.8E+05	1.6E+10	1.9E+09	7.2E+08	1.0E+08	4.1E+09	1.4E+08	4.1E+09	8.1E+07	**2.7E+04**
F1	min	8.2E+04	9.3E+10	1.6E+10	6.2E+08	1.8E+06	1.6E+10	1.4E+06	2.8E+10	1.1E+08	**4.7E+04**
F1	max	1.2E+06	1.4E+11	2.1E+10	2.2E+09	2.3E+08	2.6E+10	3.1E+08	3.8E+10	3.0E+08	**1.2E+05**
F2	mean	4.1E+04	1.6E+05	7.1E+04	1.3E+04	**8.0E+02**	4.4E+04	6.1E+03	6.1E+04	5.1E+03	7.9E+03
F2	std	2.1E+04	2.7E+04	3.7E+03	3.8E+03	**3.0E+02**	9.7E+03	3.2E+03	1.3E+04	2.3E+03	2.8E+03
F2	min	2.1E+04	1.3E+05	6.7E+04	9.8E+03	**3.2E+02**	3.6E+04	3.3E+03	4.9E+04	2.9E+03	4.1E+03
F2	max	6.4E+04	2.0E+05	7.6E+04	1.9E+04	**1.1E+03**	6.1E+04	1.1E+04	7.7E+04	8.1E+03	1.1E+04

Supplementary Table S5 Comparison with Simple Multimodal Functions 50 dimension

Function	Metrics	AO	AOA	DE	MPA	PSO	FHO	JAYA	NRBO	DBO	CBMA
F3	mean	3.8E+08	8.6E+10	2.8E+09	9.1E+07	5.5E+04	4.6E+09	**4.5E+04**	7.7E+09	5.8E+06	**7.0E+05**
F3	std	4.5E+08	2.7E+10	1.3E+09	8.1E+07	1.2E+05	9.9E+08	**4.7E+04**	3.0E+09	5.1E+06	**6.0E+05**
F3	min	9.0E+07	6.2E+10	1.5E+09	2.8E+06	1.0E+03	3.2E+09	2.3E+03	3.9E+09	8.3E+05	**7.3E+02**
F3	max	1.2E+09	1.3E+11	4.7E+09	2.1E+08	2.7E+05	5.9E+09	**1.0E+05**	1.1E+10	1.4E+07	**1.4E+06**
F4	mean	7.4E+03	1.3E+05	2.1E+04	2.9E+03	**7.1E+02**	3.0E+04	**5.7E+02**	3.6E+04	8.7E+02	7.1E+02
F4	std	1.5E+03	2.0E+04	3.6E+03	8.8E+02	**2.0E+02**	7.3E+03	**4.9E+01**	4.1E+03	1.0E+02	1.6E+02
F4	min	4.9E+03	1.1E+05	1.8E+04	2.0E+03	**5.3E+02**	2.3E+04	**5.1E+02**	3.1E+04	7.6E+02	5.4E+02
F4	max	8.7E+03	1.5E+05	2.7E+04	4.0E+03	**9.6E+02**	4.0E+04	**6.2E+02**	3.9E+04	9.9E+02	9.5E+02
F5	mean	2.2E+01	2.3E+01	2.3E+01	2.1E+01	2.1E+01	2.2E+01	2.2E+01	2.2E+01	**2.1E+01**	2.1E+01
F5	std	8.2E-01	2.7E-01	**2.2E-01**	3.5E-01	3.6E-01	7.4E-01	3.0E-01	4.9E-01	1.2E+00	4.2E-01
F5	min	2.1E+01	2.3E+01	2.2E+01	2.1E+01	2.1E+01	2.1E+01	2.2E+01	2.1E+01	**1.9E+01**	2.0E+01
F5	max	2.3E+01	2.4E+01	2.3E+01	2.2E+01	**2.1E+01**	2.3E+01	2.3E+01	2.3E+01	2.2E+01	2.1E+01
F6	mean	6.9E+03	1.2E+05	1.6E+04	2.5E+03	1.1E+03	2.2E+04	**5.8E+02**	3.6E+04	1.0E+03	8.3E+02
F6	std	2.0E+03	1.4E+04	2.3E+03	5.7E+02	4.0E+02	1.7E+03	**2.6E+01**	5.2E+03	3.4E+02	2.3E+02
F6	min	5.0E+03	1.0E+05	1.3E+04	1.5E+03	6.6E+02	2.1E+04	5.6E+02	2.7E+04	**5.1E+02**	5.9E+02
F6	max	1.0E+04	1.4E+05	1.9E+04	2.9E+03	1.6E+03	2.5E+04	**6.3E+02**	4.1E+04	1.4E+03	1.2E+03
F7	mean	7.5E+03	1.4E+05	1.8E+04	2.7E+03	1.4E+03	3.4E+04	**6.6E+02**	4.2E+04	1.1E+03	8.9E+02
F7	std	3.0E+03	1.9E+04	4.9E+03	4.6E+02	8.1E+02	3.7E+03	1.3E+02	8.8E+03	2.5E+02	**9.3E+01**
F7	min	3.7E+03	1.2E+05	1.2E+04	2.0E+03	8.2E+02	2.7E+04	**5.6E+02**	3.2E+04	8.2E+02	7.7E+02
F7	max	1.1E+04	1.7E+05	2.3E+04	3.2E+03	2.8E+03	3.6E+04	**8.8E+02**	5.2E+04	1.4E+03	9.9E+02
F8	mean	9.3E+03	3.6E+04	1.2E+04	6.9E+03	**2.7E+03**	8.1E+03	8.1E+03	1.9E+04	8.6E+03	1.3E+04
F8	std	2.5E+03	5.0E+03	**1.0E+03**	2.3E+03	1.3E+03	2.0E+03	3.7E+03	4.3E+03	1.6E+03	3.6E+03
F8	min	6.9E+03	3.0E+04	1.1E+04	4.1E+03	**9.2E+02**	5.3E+03	4.0E+03	1.3E+04	6.8E+03	8.2E+03
F8	max	1.3E+04	4.3E+04	1.4E+04	9.5E+03	**4.0E+03**	1.0E+04	1.1E+04	2.5E+04	1.0E+04	1.7E+04
F9	mean	8.9E+02	8.4E+03	3.6E+03	1.0E+03	**4.1E+02**	3.6E+03	9.2E+02	3.6E+03	1.4E+03	8.8E+02
F9	std	5.0E+02	7.4E+02	6.2E+02	4.9E+02	3.2E+02	6.5E+02	**1.3E+02**	7.4E+02	1.1E+03	4.7E+02
F9	min	3.5E+02	7.4E+03	3.0E+03	2.5E+02	**2.3E+01**	2.7E+03	6.9E+02	2.9E+03	2.4E+01	4.0E+02
F9	max	1.6E+03	9.0E+03	4.3E+03	1.5E+03	**8.4E+02**	4.4E+03	1.0E+03	4.6E+03	2.9E+03	1.4E+03

Supplementary Table S6 Comparison with Component Functions 50 dimension

Function	Metrics	AO	AOA	DE	MPA	PSO	FHO	JAYA	NRBO	DBO	CBMA
F10	mean	2.0E+03	2.7E+05	4.8E+00	3.7E-06	7.7E+02	6.0E+02	**1.8E+01**	**1.4E-28**	2.1E-01	**1.4E+02**
F10	std	1.9E+03	2.4E+05	1.1E+01	5.1E-06	1.7E+03	1.3E+03	**1.9E+01**	**2.1E-28**	1.6E-01	**2.0E+02**
F10	min	7.4E+01	2.0E+03	5.3E-03	4.3E-07	4.8E-12	1.5E-02	7.6E-01	**3.5E-30**	8.2E-05	**1.5E-01**
F10	max	5.1E+03	6.0E+05	2.4E+01	1.3E-05	3.8E+03	3.0E+03	**4.7E+01**	**5.1E-28**	4.3E-01	**4.7E+02**
F11	mean	2.4E+05	2.2E+05	1.5E+05	5.3E+04	**2.7E+04**	5.0E+04	**1.1E+05**	1.3E+05	9.0E+04	2.8E+04
F11	std	7.9E+04	6.6E+04	5.3E+04	1.5E+04	**2.1E+04**	**7.4E+03**	**2.8E+04**	2.3E+04	3.8E+04	2.2E+04
F11	min	1.9E+05	1.5E+05	9.4E+04	3.2E+04	**4.8E+03**	3.9E+04	**6.9E+04**	1.1E+05	3.6E+04	5.3E+03
F11	max	3.8E+05	3.2E+05	2.3E+05	7.0E+04	**5.2E+04**	5.8E+04	**1.4E+05**	1.5E+05	1.4E+05	6.0E+04
F12	mean	2.1E+01	2.1E+01	2.1E+01	2.0E+01	2.0E+01	2.1E+01	2.1E+01	2.1E+01	**2.1E+01**	**2.0E+01**
F12	std	8.3E-02	7.6E-02	**3.9E-02**	8.2E-02	1.7E-01	3.9E-02	**2.5E-02**	1.5E-01	5.0E-01	4.2E-02
F12	min	2.1E+01	2.1E+01	2.1E+01	2.0E+01	2.0E+01	2.1E+01	2.1E+01	2.1E+01	**2.0E+01**	2.0E+01
F12	max	2.1E+01	2.1E+01	2.1E+01	2.0E+01	**2.1E+01**	2.1E+01	2.1E+01	2.1E+01	2.1E+01	**2.0E+01**
F13	mean	7.5E+01	8.1E+01	7.5E+01	4.8E+01	6.0E+01	7.7E+01	**7.6E+01**	7.3E+01	7.5E+01	**3.5E+01**
F13	std	1.4E+00	1.7E+00	1.5E+00	3.7E+00	5.2E+00	1.8E+00	**1.5E+00**	**1.2E+00**	1.6E+00	2.7E+00
F13	min	7.4E+01	7.8E+01	7.3E+01	4.2E+01	5.5E+01	7.4E+01	7.3E+01	7.1E+01	**7.3E+01**	**3.2E+01**
F13	max	7.7E+01	8.2E+01	7.7E+01	5.2E+01	6.9E+01	7.8E+01	**7.7E+01**	7.4E+01	7.7E+01	**3.9E+01**
F14	mean	2.9E+00	3.9E+01	5.0E+00	1.4E+00	**1.3E-01**	8.4E+00	**3.0E-01**	1.2E+01	1.0E+00	8.3E-01
F14	std	8.5E-01	2.5E+00	7.9E-01	2.1E-01	1.1E-01	1.6E+00	4.2E-01	2.0E+00	**6.5E-02**	**7.7E-02**
F14	min	1.7E+00	3.6E+01	4.5E+00	1.1E+00	**5.7E-02**	6.9E+00	**6.6E-02**	9.8E+00	9.4E-01	7.3E-01
F14	max	3.7E+00	4.2E+01	6.4E+00	1.7E+00	**3.2E-01**	1.1E+01	**1.0E+00**	1.5E+01	1.1E+00	9.4E-01
F15	mean	5.5E+44	3.9E+46	3.8E+44	1.4E+34	**1.8E+43**	1.7E+45	3.0E+44	1.1E+44	2.3E+44	**1.7E+30**
F15	std	3.5E+44	7.6E+46	**4.3E+44**	3.1E+34	2.9E+43	1.1E+45	3.4E+44	1.6E+44	4.2E+44	**1.9E+30**
F15	min	3.1E+43	7.3E+43	1.0E+43	1.2E+30	**1.3E+38**	7.0E+44	3.8E+43	8.5E+40	**1.1E+27**	1.4E+28
F15	max	9.0E+44	1.7E+47	1.1E+45	7.0E+34	**6.6E+43**	3.6E+45	8.1E+44	3.8E+44	9.6E+44	**4.1E+30**
F16	mean	6.9E+01	1.5E+03	1.9E+02	3.8E+01	**5.8E-01**	3.2E+02	6.6E-01	3.7E+02	8.3E+00	**5.5E-01**
F16	std	1.6E+01	3.3E+02	4.8E+01	4.2E+01	1.6E-01	6.8E+01	**1.1E-01**	5.4E+01	4.2E+00	1.5E-01
F16	min	5.0E+01	9.6E+02	1.3E+02	1.3E+01	**4.8E-01**	2.4E+02	5.1E-01	3.1E+02	3.8E+00	**3.6E-01**
F16	max	8.4E+01	1.8E+03	2.5E+02	1.1E+02	**8.7E-01**	4.3E+02	8.1E-01	4.4E+02	1.4E+01	**7.2E-01**
F17	mean	4.4E+03	1.4E+05	1.5E+04	1.3E+03	1.5E+00	2.8E+04	8.5E-01	3.8E+04	1.6E+02	**5.4E-01**
F17	std	1.2E+03	1.6E+04	1.6E+03	5.1E+02	2.2E+00	1.6E+03	5.2E-01	1.1E+04	4.1E+01	**1.9E-01**
F17	min	2.8E+03	1.2E+05	1.2E+04	4.9E+02	**3.3E-01**	2.6E+04	4.2E-01	2.2E+04	1.2E+02	3.6E-01
F17	max	6.2E+03	1.6E+05	1.6E+04	1.7E+03	5.5E+00	3.0E+04	1.7E+00	5.2E+04	2.1E+02	**7.7E-01**
F18	mean	2.6E+01	2.9E+01	2.8E+01	2.5E+01	2.5E+01	2.6E+01	2.7E+01	2.6E+01	**2.3E+01**	2.5E+01
F18	std	1.7E+00	6.4E-01	**3.3E-01**	8.7E-01	8.5E-01	6.7E-01	4.2E-01	1.2E+00	2.2E+00	1.8E+00
F18	min	2.3E+01	2.8E+01	2.7E+01	2.4E+01	2.4E+01	2.5E+01	2.7E+01	2.5E+01	**2.1E+01**	2.2E+01
F18	max	2.8E+01	3.0E+01	2.8E+01	2.6E+01	**2.6E+01**	2.7E+01	2.8E+01	2.8E+01	2.6E+01	2.6E+01
F19	mean	3.4E+00	2.9E+01	2.3E+01	1.4E+00	**5.3E-01**	1.4E+01	2.6E+01	1.1E+01	1.1E+00	5.8E-01
F19	std	1.5E+00	6.7E+00	1.2E+00	8.8E-01	**3.5E-01**	7.6E-01	3.2E+00	2.3E+00	9.8E-01	4.9E-01
F19	min	2.0E+00	2.4E+01	2.1E+01	5.3E-01	**7.7E-02**	1.3E+01	2.2E+01	8.5E+00	4.4E-01	1.4E-01
F19	max	5.4E+00	3.9E+01	2.4E+01	2.8E+00	**8.6E-01**	1.5E+01	3.0E+01	1.3E+01	2.6E+00	1.3E+00

Supplementary Table S7 Comparison with Hybrid Functions 50 dimension

Function	Metrics	AO	AOA	DE	MPA	PSO	FHO	JAYA	NRBO	DBO	CBMA
F20	mean	1.3E+08	3.9E+10	7.4E+08	8.5E+06	**4.3E+03**	1.8E+09	**9.6E+06**	**4.0E+09**	1.4E+06	**7.5E+03**
F20	std	7.4E+07	1.0E+10	2.2E+08	6.2E+06	**5.3E+03**	1.2E+09	**2.1E+07**	**2.4E+09**	7.8E+05	**1.4E+04**
F20	min	6.8E+07	2.8E+10	4.4E+08	3.3E+06	3.6E+02	5.3E+08	2.7E+03	**2.1E+09**	5.3E+05	**3.4E+02**
F20	max	2.4E+08	5.3E+10	1.0E+09	1.9E+07	**1.1E+04**	3.5E+09	**4.8E+07**	**7.6E+09**	2.3E+06	**3.3E+04**
F21	mean	2.5E+09	5.3E+10	6.8E+09	5.9E+08	**4.8E+06**	1.1E+10	**1.1E+06**	1.4E+10	7.1E+07	**1.2E+05**
F21	std	8.8E+08	6.8E+09	1.1E+09	1.7E+08	**6.2E+06**	**2.6E+09**	**3.1E+05**	1.6E+09	2.8E+07	**1.9E+05**
F21	min	1.8E+09	4.7E+10	5.4E+09	3.2E+08	**2.2E+05**	8.7E+09	**8.4E+05**	1.1E+10	4.1E+07	**2.2E+04**
F21	max	4.0E+09	6.4E+10	8.4E+09	7.5E+08	**1.6E+07**	1.5E+10	**1.6E+06**	1.5E+10	1.2E+08	**4.6E+05**
F22	mean	1.9E+09	7.3E+10	5.2E+09	5.5E+08	1.4E+06	9.9E+09	1.2E+06	1.5E+10	**6.2E+07**	**3.4E+05**
F22	std	5.3E+08	1.4E+10	**1.4E+09**	2.3E+08	1.7E+06	1.7E+09	**3.5E+05**	3.6E+09	4.8E+07	4.2E+05
F22	min	1.3E+09	5.5E+10	3.3E+09	2.9E+08	3.1E+04	6.9E+09	8.1E+05	1.0E+10	**2.9E+07**	**2.8E+04**
F22	max	2.5E+09	8.6E+10	7.2E+09	9.2E+08	**3.7E+06**	1.1E+10	1.6E+06	2.0E+10	1.4E+08	**1.0E+06**
F23	mean	8.7E+04	1.3E+05	4.1E+04	1.2E+04	7.7E+03	2.2E+04	**3.7E+04**	3.9E+04	3.6E+04	**7.0E+03**
F23	std	4.5E+04	1.2E+05	1.8E+04	4.3E+03	4.8E+03	1.2E+04	**1.8E+04**	**1.2E+04**	1.6E+04	**4.2E+03**
F23	min	2.0E+04	6.6E+04	2.1E+04	6.9E+03	**1.5E+03**	1.5E+04	8.9E+03	2.3E+04	**1.2E+04**	**4.2E+03**
F23	max	1.4E+05	3.5E+05	6.6E+04	1.6E+04	**1.3E+04**	4.3E+04	**5.4E+04**	5.4E+04	5.4E+04	**1.4E+04**
F24	mean	7.9E+07	3.0E+10	7.9E+08	6.3E+06	**3.4E+03**	9.8E+08	**2.3E+04**	3.5E+09	7.1E+05	3.2E+04
F24	std	5.6E+07	5.8E+09	1.1E+08	4.4E+06	**5.7E+03**	5.0E+08	4.4E+04	2.7E+09	**6.9E+05**	**4.7E+04**
F24	min	2.5E+07	2.3E+10	6.1E+08	5.6E+05	**4.3E+02**	2.7E+08	**7.2E+02**	7.9E+08	1.2E+05	**1.5E+02**
F24	max	1.4E+08	3.7E+10	8.9E+08	1.0E+07	**1.4E+04**	1.7E+09	**1.0E+05**	7.2E+09	1.8E+06	1.1E+05
F25	mean	1.2E+44	1.2E+46	1.7E+43	2.5E+33	**1.8E+38**	9.8E+43	2.9E+43	2.2E+42	1.2E+43	**1.8E+29**
F25	std	8.6E+43	1.5E+46	**1.2E+43**	5.2E+33	3.5E+38	9.9E+43	5.1E+43	4.2E+42	4.8E+42	**2.4E+29**
F25	min	4.4E+43	8.9E+42	2.3E+42	3.6E+29	**1.3E+34**	6.0E+42	2.8E+42	8.5E+38	**6.2E+42**	**3.5E+25**
F25	max	2.5E+44	3.0E+46	2.9E+43	1.2E+34	**8.1E+38**	2.1E+44	1.2E+44	9.6E+42	1.8E+43	**6.0E+29**
F26	mean	9.8E+08	2.7E+10	3.5E+09	2.8E+08	**2.9E+05**	5.0E+09	5.0E+05	8.1E+09	3.8E+07	**2.7E+04**
F26	std	5.8E+08	1.8E+09	5.8E+08	9.9E+07	2.7E+05	1.3E+09	**2.6E+05**	1.6E+09	2.1E+07	**3.4E+04**
F26	min	6.1E+08	2.5E+10	2.8E+09	1.6E+08	**3.5E+04**	3.2E+09	2.4E+05	6.9E+09	1.6E+07	**3.7E+03**
F26	max	2.0E+09	2.9E+10	4.2E+09	4.1E+08	**7.3E+05**	6.3E+09	8.5E+05	1.1E+10	6.6E+07	**8.6E+04**
F27	mean	1.1E+09	3.0E+10	3.1E+09	3.7E+08	2.7E+05	6.6E+09	1.3E+07	8.0E+09	4.7E+07	**2.1E+04**
F27	std	4.0E+08	2.3E+09	6.6E+08	2.2E+08	2.4E+05	1.0E+09	2.7E+07	1.8E+09	5.3E+07	**1.3E+04**
F27	min	7.9E+08	2.7E+10	2.1E+09	1.5E+08	**5.9E+04**	5.9E+09	1.7E+05	5.2E+09	7.8E+06	**7.1E+03**
F27	max	1.8E+09	3.4E+10	3.8E+09	7.0E+08	6.6E+05	8.3E+09	6.1E+07	1.0E+10	1.4E+08	**3.4E+04**
F28	mean	2.4E+44	1.8E+45	3.3E+43	4.1E+31	1.2E+42	1.0E+44	3.1E+43	7.1E+41	**2.4E+43**	**6.2E+29**
F28	std	1.6E+44	2.0E+45	**4.8E+43**	7.1E+31	2.3E+42	1.8E+44	3.3E+43	7.1E+41	5.1E+43	**1.3E+30**
F28	min	9.5E+40	3.0E+43	7.4E+40	8.9E+28	2.0E+36	8.7E+42	1.5E+42	1.5E+40	**1.1E+34**	**1.8E+25**
F28	max	4.6E+44	4.7E+45	1.2E+44	1.7E+32	**5.4E+42**	4.3E+44	7.9E+43	1.9E+42	1.1E+44	**3.0E+30**
F29	mean	3.6E+08	6.7E+10	2.9E+09	3.4E+07	**2.0E+05**	4.5E+09	**3.6E+03**	8.9E+09	2.6E+06	8.1E+05
F29	std	1.5E+08	7.2E+09	1.0E+09	1.6E+07	**4.2E+05**	2.2E+09	**2.3E+03**	5.2E+09	2.1E+06	7.8E+05
F29	min	1.7E+08	6.1E+10	1.5E+09	1.9E+07	**1.1E+03**	2.2E+09	1.4E+03	3.2E+09	5.0E+05	**4.9E+02**
F29	max	4.9E+08	7.9E+10	4.4E+09	5.8E+07	**9.6E+05**	7.7E+09	**7.4E+03**	1.6E+10	5.0E+06	1.7E+06

Supplementary Table S8 Comparison with Composition Functions 50 dimension

Function	Metrics	AO	AOA	DE	MPA	PSO	FHO	JAYA	NRBO	DBO	CBMA
F30	mean	9.6E+02	8.9E+03	3.0E+03	9.6E+02	**3.6E+02**	3.5E+03	**6.7E+02**	**3.6E+03**	2.0E+03	**6.1E+02**
F30	std	3.6E+02	1.3E+03	4.7E+02	4.3E+02	**2.6E+02**	4.9E+02	**7.9E+02**	**8.5E+02**	7.0E+02	**1.3E+02**
F30	min	5.9E+02	7.5E+03	2.3E+03	4.5E+02	**5.8E+00**	2.9E+03	2.2E+02	**2.9E+03**	1.2E+03	**4.8E+02**
F30	max	1.5E+03	1.1E+04	3.6E+03	1.6E+03	**7.1E+02**	4.3E+03	**2.1E+03**	**5.1E+03**	2.6E+03	**7.8E+02**
F31	mean	3.7E+04	1.8E+05	1.8E+04	3.6E+03	**1.3E+03**	3.9E+04	**6.5E+02**	5.3E+04	1.2E+03	**7.4E+02**
F31	std	1.8E+04	5.1E+04	3.2E+03	1.2E+03	**5.7E+02**	**4.9E+03**	**1.3E+02**	7.6E+03	2.5E+02	**1.8E+02**
F31	min	1.1E+04	1.3E+05	1.4E+04	2.6E+03	**4.4E+02**	3.1E+04	**5.3E+02**	4.5E+04	8.9E+02	**4.9E+02**
F31	max	6.1E+04	2.3E+05	2.3E+04	5.6E+03	**2.0E+03**	4.5E+04	**8.6E+02**	6.4E+04	1.6E+03	**9.3E+02**
F32	mean	4.5E+04	4.6E+04	1.7E+04	2.7E+04	1.2E+03	3.2E+04	**5.7E+02**	4.6E+04	**1.1E+03**	**7.7E+02**
F32	std	3.4E+03	3.3E+03	**2.4E+03**	2.2E+04	6.0E+02	4.2E+03	**3.3E+01**	3.4E+03	4.0E+02	2.3E+02
F32	min	4.1E+04	4.2E+04	1.4E+04	1.8E+03	5.7E+02	2.7E+04	5.3E+02	4.1E+04	**5.4E+02**	**4.8E+02**
F32	max	4.9E+04	5.0E+04	1.9E+04	4.5E+04	**1.9E+03**	3.8E+04	**6.2E+02**	4.9E+04	1.6E+03	**1.1E+03**
F33	mean	4.2E+09	2.5E+10	3.2E+09	8.3E+07	**4.3E+03**	3.8E+09	**4.6E+03**	1.9E+10	2.1E+06	**2.3E+05**
F33	std	8.8E+09	2.8E+09	2.0E+09	1.3E+08	3.6E+03	1.5E+09	**3.1E+03**	**8.9E+09**	2.0E+06	**5.2E+05**
F33	min	6.7E+07	2.1E+10	1.2E+09	1.6E+07	**1.4E+03**	1.5E+09	1.0E+03	4.5E+09	**1.6E+05**	**3.0E+02**
F33	max	2.0E+10	2.8E+10	6.4E+09	3.1E+08	**1.0E+04**	5.2E+09	**9.0E+03**	2.7E+10	4.9E+06	**1.2E+06**
F34	mean	5.3E+02	1.2E+08	1.4E+02	4.1E+02	**5.7E+02**	**9.7E+01**	**3.7E+02**	4.8E+02	2.1E+02	3.5E+02
F34	std	1.5E+02	5.5E+07	4.5E+01	7.5E+01	**9.5E+01**	**1.0E+01**	1.6E+02	6.1E+01	**1.6E+02**	**7.9E+01**
F34	min	3.1E+02	4.3E+07	9.4E+01	2.9E+02	**4.5E+02**	**9.0E+01**	**1.2E+02**	4.1E+02	9.1E+01	**2.5E+02**
F34	max	7.0E+02	1.9E+08	1.9E+02	4.8E+02	**6.9E+02**	**1.1E+02**	**5.3E+02**	5.8E+02	4.3E+02	4.6E+02
F35	mean	-1.3E+01	-1.1E+01	-7.6E+00	-1.5E+01	**-1.7E+01**	-1.0E+01	-1.4E+01	-1.3E+01	-1.5E+01	**-1.5E+01**
F35	std	**3.2E-01**	8.7E-01	**2.7E+00**	7.8E-01	6.9E-01	2.5E+00	1.6E+00	5.6E-01	1.4E+00	**9.5E-01**
F35	min	-1.4E+01	-1.2E+01	-1.2E+01	-1.6E+01	**-1.8E+01**	-1.3E+01	-1.7E+01	-1.4E+01	**-1.7E+01**	**-1.7E+01**
F35	max	-1.3E+01	-9.9E+00	-5.1E+00	-1.4E+01	**-1.6E+01**	-6.0E+00	-1.3E+01	-1.2E+01	-1.3E+01	**-1.4E+01**
F36	mean	1.3E-02	1.4E+04	**1.1E-02**	1.2E-02	**1.3E-02**	1.0E+00	1.0E+00	1.0E+00	1.0E+00	**1.2E-02**
F36	std	2.0E-04	6.0E+03	3.5E-04	2.0E-04	2.5E-04	3.9E-04	**2.5E-04**	**9.1E-05**	6.0E-04	**2.5E-04**
F36	min	1.2E-02	7.2E+03	**1.1E-02**	1.2E-02	**1.2E-02**	1.0E+00	1.0E+00	1.0E+00	1.0E+00	**1.2E-02**
F36	max	1.3E-02	2.0E+04	**1.2E-02**	1.3E-02	**1.3E-02**	1.0E+00	1.0E+00	1.0E+00	1.0E+00	**1.3E-02**
F37	mean	2.7E+18	2.3E+37	3.7E+14	**1.2E+11**	5.1E+20	6.5E+17	1.5E+16	1.8E+15	1.1E+13	**8.1E+15**
F37	std	3.0E+18	3.1E+37	4.2E+14	**1.5E+10**	1.1E+21	6.0E+17	3.0E+16	2.4E+15	2.1E+13	**9.4E+15**
F37	min	7.5E+16	1.0E+36	8.8E+12	1.0E+11	**1.0E+12**	1.4E+17	9.7E+13	2.1E+13	**9.1E+10**	**6.6E+14**
F37	max	6.1E+18	7.5E+37	1.0E+15	**1.3E+11**	2.4E+21	1.5E+18	7.0E+16	5.2E+15	4.9E+13	**2.3E+16**
F38	mean	3.1E+44	2.6E+45	2.0E+43	8.9E+32	1.1E+41	7.3E+43	3.4E+43	7.9E+42	**5.9E+42**	**3.7E+31**
F38	std	4.5E+44	3.7E+45	**1.7E+43**	1.5E+33	2.5E+41	6.4E+43	4.4E+43	1.1E+43	1.0E+43	**5.1E+31**
F38	min	5.5E+42	1.2E+43	1.1E+42	2.7E+29	4.4E+32	2.0E+43	1.8E+40	1.4E+39	**1.9E+35**	**2.2E+23**
F38	max	1.0E+45	8.4E+45	3.9E+43	3.4E+33	**5.5E+41**	1.8E+44	1.0E+44	2.2E+43	2.4E+43	**9.4E+31**

S2.2 100D

Supplementary Table S9 Comparison with Unimodal Functions 100 dimension

Function	Metrics	AO	AOA	DE	MPA	PSO	FHO	JAYA	NRBO	DBO	CBMA
F1	mean	3.4E+10	3.0E+11	9.6E+10	3.2E+10	3.5E+09	6.6E+10	9.9E+08	1.4E+11	4.2E+09	**3.0E+08**
F1	std	1.1E+10	3.9E+10	5.2E+09	9.3E+09	2.1E+09	5.9E+09	2.5E+08	3.1E+10	2.4E+09	**9.5E+07**
F1	min	2.3E+10	2.6E+11	8.8E+10	2.0E+10	6.6E+08	6.1E+10	6.2E+08	9.8E+10	1.6E+09	**2.3E+08**
F1	max	4.9E+10	3.6E+11	1.0E+11	4.4E+10	5.6E+09	7.5E+10	1.3E+09	1.8E+11	7.9E+09	**4.5E+08**
F2	mean	1.9E+05	3.4E+05	3.3E+05	1.3E+05	**3.9E+04**	1.0E+05	1.3E+05	1.9E+05	7.1E+04	9.5E+04
F2	std	7.1E+04	4.0E+04	3.7E+04	2.3E+04	1.1E+04	1.8E+04	3.3E+04	2.0E+04	4.0E+04	**6.4E+03**
F2	min	1.1E+05	3.0E+05	3.1E+05	1.0E+05	**2.9E+04**	8.6E+04	9.1E+04	1.6E+05	3.2E+04	8.7E+04
F2	max	3.0E+05	4.0E+05	4.0E+05	1.6E+05	**5.6E+04**	1.3E+05	1.6E+05	2.1E+05	1.4E+05	1.0E+05

Supplementary Table S10 Comparison with Simple Multimodal Functions 100 dimension

Function	Metrics	AO	AOA	DE	MPA	PSO	FHO	JAYA	NRBO	DBO	CBMA
F3	mean	4.6E+09	2.3E+11	3.8E+10	3.7E+09	6.5E+07	1.1E+10	2.9E+07	4.4E+10	6.4E+09	**5.2E+06**
F3	std	2.7E+09	4.8E+10	4.8E+09	1.2E+09	8.9E+07	2.4E+09	9.2E+06	8.5E+09	1.3E+10	**3.9E+06**
F3	min	2.1E+09	1.7E+11	3.1E+10	2.5E+09	5.6E+06	9.2E+09	2.0E+07	3.5E+10	7.1E+07	**3.8E+05**
F3	max	8.7E+09	3.0E+11	4.4E+10	5.7E+09	2.2E+08	1.5E+10	4.1E+07	5.4E+10	2.9E+10	**9.5E+06**
F4	mean	3.7E+04	3.0E+05	1.0E+05	3.0E+04	**9.5E+03**	7.3E+04	**2.4E+03**	1.4E+05	8.1E+03	7.2E+03
F4	std	5.7E+03	3.1E+04	1.4E+04	6.5E+03	2.2E+03	1.2E+04	**4.3E+02**	2.2E+04	3.4E+03	**1.3E+03**
F4	min	2.9E+04	2.6E+05	8.5E+04	2.2E+04	**7.0E+03**	5.5E+04	**1.8E+03**	1.1E+05	3.8E+03	5.9E+03
F4	max	4.3E+04	3.4E+05	1.2E+05	3.6E+04	**1.2E+04**	8.5E+04	**2.8E+03**	1.6E+05	1.3E+04	9.2E+03
F5	mean	4.6E+01	4.8E+01	4.7E+01	**4.4E+01**	4.5E+01	4.5E+01	4.7E+01	4.6E+01	4.5E+01	4.4E+01
F5	std	8.0E-01	4.1E-01	2.0E-01	7.3E-01	9.8E-01	5.4E-01	**1.8E-01**	4.5E-01	1.4E+00	8.4E-01
F5	min	4.5E+01	4.7E+01	4.7E+01	4.3E+01	4.4E+01	4.5E+01	4.7E+01	4.5E+01	**4.3E+01**	4.3E+01
F5	max	4.7E+01	4.8E+01	4.8E+01	4.5E+01	4.6E+01	4.6E+01	4.7E+01	4.6E+01	4.6E+01	**4.5E+01**
F6	mean	2.6E+04	3.0E+05	9.2E+04	3.0E+04	8.8E+03	6.4E+04	**2.4E+03**	1.4E+05	5.9E+03	7.9E+03
F6	std	6.6E+03	4.6E+04	1.2E+04	7.4E+03	1.9E+03	1.1E+04	**4.8E+02**	1.2E+04	1.9E+03	9.6E+02
F6	min	1.8E+04	2.7E+05	7.4E+04	2.1E+04	6.8E+03	5.2E+04	**2.1E+03**	1.3E+05	4.5E+03	6.6E+03
F6	max	3.3E+04	3.8E+05	1.0E+05	3.8E+04	1.1E+04	8.1E+04	**3.2E+03**	1.6E+05	9.2E+03	9.3E+03
F7	mean	3.6E+04	3.2E+05	1.0E+05	2.8E+04	1.0E+04	7.6E+04	**2.4E+03**	1.5E+05	6.4E+03	8.4E+03
F7	std	1.2E+04	2.8E+04	8.9E+03	7.1E+03	2.0E+03	8.5E+03	**2.9E+02**	1.6E+04	9.7E+02	2.0E+03
F7	min	2.4E+04	2.8E+05	9.0E+04	2.1E+04	7.1E+03	6.5E+04	**2.0E+03**	1.3E+05	5.0E+03	6.6E+03
F7	max	5.3E+04	3.6E+05	1.1E+05	4.0E+04	1.2E+04	8.5E+04	**2.7E+03**	1.7E+05	7.6E+03	1.1E+04
F8	mean	2.6E+04	1.0E+05	5.7E+04	3.0E+04	**1.7E+04**	2.3E+04	5.6E+04	6.2E+04	3.1E+04	4.5E+04
F8	std	7.3E+03	6.3E+03	7.7E+03	4.2E+03	5.6E+03	**3.0E+03**	1.8E+04	3.8E+03	6.0E+03	8.3E+03
F8	min	1.8E+04	9.0E+04	4.4E+04	2.8E+04	**1.1E+04**	2.0E+04	4.3E+04	5.6E+04	2.5E+04	3.6E+04
F8	max	3.5E+04	1.1E+05	6.3E+04	3.8E+04	**2.4E+04**	2.7E+04	8.8E+04	6.5E+04	3.8E+04	5.6E+04
F9	mean	7.4E+03	1.9E+04	1.4E+04	5.5E+03	**1.2E+03**	8.4E+03	2.9E+03	1.2E+04	5.3E+03	1.8E+03
F9	std	2.4E+03	1.5E+03	7.9E+02	1.4E+03	6.3E+02	1.2E+03	9.0E+02	1.2E+03	2.3E+03	**4.1E+02**
F9	min	3.1E+03	1.7E+04	1.4E+04	3.6E+03	**3.9E+02**	7.1E+03	2.3E+03	1.0E+04	2.4E+03	1.4E+03
F9	max	8.8E+03	2.1E+04	1.5E+04	7.5E+03	**2.1E+03**	9.7E+03	4.4E+03	1.3E+04	7.9E+03	2.3E+03

Supplementary Table S11 Comparison with Component Functions 100 dimension

Function	Metrics	AO	AOA	DE	MPA	PSO	FHO	JAYA	NRBO	DBO	CBMA
F10	mean	8.5E+02	2.4E+06	2.5E+01	7.4E-06	4.4E+00	1.2E+02	2.3E+01	**2.3E-29**	1.3E+02	1.5E+03
F10	std	1.6E+03	5.4E+06	3.1E+01	5.1E-06	6.6E+00	2.2E+02	2.5E+01	**3.3E-29**	2.4E+02	2.8E+03
F10	min	9.7E+00	4.0E+02	2.1E-05	3.1E-06	**3.4E-31**	5.3E+00	4.0E-04	7.0E-31	3.1E-20	1.9E+01
F10	max	3.7E+03	1.2E+07	7.7E+01	1.5E-05	1.6E+01	5.2E+02	5.7E+01	**8.0E-29**	5.6E+02	6.4E+03
F11	mean	3.8E+05	1.0E+06	3.7E+05	1.6E+05	8.7E+04	1.5E+05	3.3E+05	2.4E+05	1.9E+05	**4.6E+04**
F11	std	1.4E+05	9.8E+05	1.6E+05	6.0E+04	4.7E+04	1.1E+05	1.9E+05	4.7E+04	7.0E+04	**3.4E+04**
F11	min	1.8E+05	3.6E+05	1.9E+05	8.8E+04	**4.1E+03**	7.4E+04	8.3E+03	1.8E+05	1.3E+05	1.7E+04
F11	max	5.0E+05	2.7E+06	5.5E+05	2.3E+05	1.2E+05	3.4E+05	4.6E+05	3.1E+05	2.7E+05	**9.8E+04**
F12	mean	2.1E+01	2.1E+01	2.1E+01	2.1E+01	2.1E+01	2.1E+01	2.1E+01	2.1E+01	2.1E+01	**2.0E+01**
F12	std	4.0E-02	2.4E-02	2.2E-02	7.3E-02	2.5E-01	2.9E-02	**1.1E-02**	8.0E-02	4.9E-01	3.2E-02
F12	min	2.1E+01	2.1E+01	2.1E+01	2.1E+01	2.1E+01	2.1E+01	2.1E+01	2.1E+01	2.0E+01	**2.0E+01**
F12	max	2.1E+01	2.1E+01	2.1E+01	2.1E+01	2.1E+01	2.1E+01	2.1E+01	2.1E+01	2.1E+01	**2.0E+01**
F13	mean	1.6E+02	1.8E+02	1.6E+02	1.2E+02	1.5E+02	1.7E+02	1.6E+02	1.6E+02	1.5E+02	**9.7E+01**
F13	std	3.9E+00	2.4E+00	3.3E+00	4.5E+00	1.0E+01	2.1E+00	**1.7E+00**	6.6E+00	2.2E+01	4.7E+00
F13	min	1.6E+02	1.7E+02	1.6E+02	1.1E+02	1.4E+02	1.6E+02	1.6E+02	1.5E+02	1.2E+02	**9.1E+01**
F13	max	1.7E+02	1.8E+02	1.7E+02	1.2E+02	1.6E+02	1.7E+02	1.7E+02	1.6E+02	1.7E+02	**1.0E+02**
F14	mean	9.2E+00	8.0E+01	2.9E+01	7.6E+00	2.0E+00	1.8E+01	**1.3E+00**	3.8E+01	2.5E+00	1.4E+00
F14	std	8.9E-01	3.8E+00	1.7E+00	1.2E+00	3.8E-01	2.2E+00	**8.2E-02**	2.2E+00	6.6E-01	8.3E-02
F14	min	8.3E+00	7.5E+01	2.8E+01	6.2E+00	1.5E+00	1.4E+01	**1.2E+00**	3.5E+01	1.6E+00	1.3E+00
F14	max	1.1E+01	8.4E+01	3.2E+01	9.0E+00	2.5E+00	2.0E+01	**1.4E+00**	4.1E+01	3.5E+00	1.5E+00
F15	mean	4.6E+108	3.9E+111	1.0E+108	2.5E+99	1.4E+107	1.2E+109	1.5E+107	1.4E+106	6.5E+107	**4.7E+84**
F15	std	7.7E+108	7.6E+111	9.5E+107	5.5E+99	2.9E+107	1.6E+109	2.0E+107	1.3E+106	4.9E+107	**7.0E+84**
F15	min	2.5E+106	7.2E+109	1.6E+106	5.7E+85	1.2E+101	8.6E+106	2.1E+104	2.1E+104	7.2E+106	**8.5E+78**
F15	max	1.8E+109	1.7E+112	2.2E+108	1.2E+100	6.6E+107	4.0E+109	4.8E+107	2.7E+106	1.1E+108	**1.6E+85**
F16	mean	2.0E+02	1.7E+03	5.4E+02	1.3E+02	2.6E+01	3.7E+02	1.1E+01	8.1E+02	4.4E+01	**1.1E+01**
F16	std	5.7E+01	1.7E+02	3.3E+01	4.0E+01	1.7E+01	5.9E+01	2.0E+00	1.2E+02	1.9E+01	**1.7E+00**
F16	min	1.3E+02	1.5E+03	5.0E+02	9.1E+01	1.1E+01	3.0E+02	9.6E+00	6.1E+02	2.6E+01	**8.4E+00**
F16	max	2.8E+02	1.8E+03	5.8E+02	2.0E+02	5.5E+01	4.4E+02	1.5E+01	9.3E+02	7.3E+01	**1.3E+01**
F17	mean	2.9E+04	3.2E+05	1.0E+05	2.9E+04	3.3E+03	7.8E+04	1.1E+03	1.4E+05	4.3E+03	**5.8E+02**
F17	std	1.1E+04	2.3E+04	1.1E+04	7.5E+03	1.4E+03	7.3E+03	2.4E+02	1.9E+04	1.4E+03	**2.1E+02**
F17	min	2.3E+04	2.8E+05	9.2E+04	1.9E+04	2.3E+03	6.9E+04	7.8E+02	1.2E+05	2.8E+03	**3.8E+02**
F17	max	4.8E+04	3.5E+05	1.2E+05	3.8E+04	5.4E+03	8.8E+04	1.4E+03	1.7E+05	6.1E+03	**9.2E+02**
F18	mean	5.3E+01	5.8E+01	5.8E+01	5.4E+01	**5.3E+01**	5.4E+01	5.8E+01	5.6E+01	5.3E+01	5.4E+01
F18	std	1.7E+00	1.2E+00	3.3E-01	5.9E-01	1.0E+00	4.3E-01	**1.3E-01**	1.4E+00	7.0E-01	1.1E+00
F18	min	**5.1E+01**	5.7E+01	5.8E+01	5.3E+01	5.2E+01	5.4E+01	5.8E+01	5.4E+01	5.3E+01	5.2E+01
F18	max	5.5E+01	6.0E+01	5.9E+01	5.4E+01	5.5E+01	5.5E+01	5.8E+01	5.7E+01	**5.4E+01**	5.5E+01
F19	mean	6.8E+00	4.4E+01	4.5E+01	3.9E+00	**1.1E+00**	1.6E+01	3.9E+01	2.4E+01	3.0E+00	7.7E+00
F19	std	7.9E-01	4.6E+00	3.3E+00	1.2E+00	**7.8E-01**	2.2E+00	4.5E+00	3.2E+00	1.2E+00	3.5E+00
F19	min	5.8E+00	3.9E+01	4.0E+01	2.5E+00	**4.0E-01**	1.4E+01	3.2E+01	2.0E+01	1.3E+00	3.4E+00
F19	max	8.0E+00	5.1E+01	4.8E+01	5.6E+00	**2.1E+00**	1.8E+01	4.4E+01	2.8E+01	4.5E+00	1.2E+01

Supplementary Table S12 Comparison with Hybrid Functions 100 dimension

Function	Metrics	AO	AOA	DE	MPA	PSO	FHO	JAYA	NRBO	DBO	CBMA
F20	mean	1.4E+09	8.7E+10	2.0E+10	1.5E+09	2.5E+07	6.4E+09	1.8E+07	2.2E+10	9.6E+07	**1.5E+06**
F20	std	5.0E+08	1.3E+10	4.6E+09	6.6E+08	3.1E+07	1.1E+09	1.1E+07	5.5E+09	4.9E+07	**3.5E+05**
F20	min	8.9E+08	7.4E+10	1.3E+10	1.0E+09	1.9E+06	5.5E+09	7.0E+06	1.6E+10	5.1E+07	**1.0E+06**
F20	max	2.1E+09	1.1E+11	2.5E+10	2.6E+09	7.8E+07	8.1E+09	3.7E+07	3.0E+10	1.6E+08	**2.0E+06**
F21	mean	1.1E+10	1.2E+11	4.1E+10	1.1E+10	1.0E+09	2.8E+10	4.2E+08	5.5E+10	1.7E+09	**1.2E+08**
F21	std	3.4E+09	7.8E+09	4.9E+09	1.5E+09	1.2E+09	2.6E+09	7.7E+07	5.9E+09	1.0E+09	**4.0E+07**
F21	min	9.0E+09	1.1E+11	3.4E+10	9.3E+09	2.9E+08	2.4E+10	3.2E+08	4.9E+10	1.1E+09	**7.7E+07**
F21	max	1.7E+10	1.3E+11	4.6E+10	1.3E+10	3.1E+09	3.0E+10	5.2E+08	6.3E+10	3.4E+09	**1.8E+08**
F22	mean	1.2E+10	1.6E+11	4.5E+10	1.0E+10	8.9E+08	2.3E+10	4.3E+08	5.9E+10	1.2E+10	**1.3E+08**
F22	std	4.6E+09	2.1E+10	1.4E+10	3.2E+09	3.1E+08	3.9E+09	1.3E+08	1.1E+10	2.0E+10	**3.1E+07**
F22	min	7.0E+09	1.4E+11	2.6E+10	6.2E+09	5.2E+08	1.7E+10	2.8E+08	4.9E+10	1.2E+09	**1.1E+08**
F22	max	1.8E+10	1.8E+11	6.5E+10	1.4E+10	1.3E+09	2.7E+10	6.1E+08	7.8E+10	4.8E+10	**1.8E+08**
F23	mean	2.1E+05	4.2E+06	1.5E+05	7.4E+04	3.5E+04	5.0E+04	1.2E+05	1.1E+05	6.8E+04	**1.8E+04**
F23	std	3.3E+04	6.5E+06	3.0E+04	1.4E+04	1.0E+04	1.6E+04	1.3E+04	**3.7E+03**	2.2E+04	6.0E+03
F23	min	1.6E+05	1.4E+05	9.9E+04	5.2E+04	2.3E+04	3.2E+04	1.0E+05	1.1E+05	4.4E+04	**1.1E+04**
F23	max	2.5E+05	1.6E+07	1.8E+05	8.9E+04	4.9E+04	7.2E+04	1.4E+05	1.2E+05	9.1E+04	**2.8E+04**
F24	mean	6.8E+08	7.1E+10	1.3E+10	9.1E+08	1.8E+07	3.9E+09	1.1E+07	1.8E+10	4.0E+07	**6.4E+05**
F24	std	3.1E+08	1.6E+10	2.3E+09	2.6E+08	1.3E+07	8.4E+08	4.2E+06	4.9E+09	3.0E+07	**2.6E+05**
F24	min	3.6E+08	5.5E+10	1.0E+10	6.5E+08	2.1E+06	3.3E+09	6.1E+06	1.4E+10	1.7E+07	**2.7E+05**
F24	max	1.1E+09	9.8E+10	1.6E+10	1.3E+09	3.0E+07	5.4E+09	1.8E+07	2.6E+10	9.2E+07	**9.0E+05**
F25	mean	1.6E+107	4.6E+109	4.2E+106	1.2E+96	2.5E+104	3.8E+107	2.7E+106	6.0E+105	6.7E+106	**2.3E+82**
F25	std	2.8E+107	7.7E+109	1.8E+106	2.7E+96	5.5E+104	6.7E+107	3.7E+106	1.2E+106	1.2E+107	**4.7E+82**
F25	min	6.7E+105	9.3E+106	1.8E+106	3.2E+87	3.0E+96	3.7E+105	1.2E+105	3.2E+102	7.1E+105	**6.5E+79**
F25	max	6.7E+107	1.8E+110	6.2E+106	6.0E+96	1.2E+105	1.6E+108	9.3E+106	2.8E+106	2.8E+107	**1.1E+83**
F26	mean	7.0E+09	6.2E+10	2.1E+10	4.9E+09	7.1E+08	1.4E+10	1.8E+08	2.8E+10	1.3E+09	**8.9E+07**
F26	std	1.5E+09	3.7E+09	2.0E+09	9.2E+08	3.1E+08	1.2E+09	5.1E+07	3.3E+09	5.6E+08	**3.2E+07**
F26	min	4.8E+09	5.7E+10	1.9E+10	3.9E+09	3.8E+08	1.2E+10	1.4E+08	2.6E+10	8.1E+08	**6.2E+07**
F26	max	8.5E+09	6.8E+10	2.3E+10	6.0E+09	1.0E+09	1.5E+10	2.7E+08	3.4E+10	2.1E+09	**1.4E+08**
F27	mean	6.4E+09	6.5E+10	2.0E+10	6.0E+09	8.5E+08	1.5E+10	1.8E+08	3.1E+10	8.0E+08	**1.1E+08**
F27	std	1.1E+09	5.8E+09	2.0E+09	1.8E+09	3.8E+08	1.3E+09	9.1E+07	4.5E+09	2.8E+08	**4.5E+07**
F27	min	4.6E+09	6.0E+10	1.8E+10	4.3E+09	5.5E+08	1.4E+10	1.1E+08	2.3E+10	4.7E+08	**6.2E+07**
F27	max	7.3E+09	7.4E+10	2.3E+10	8.9E+09	1.5E+09	1.7E+10	3.3E+08	3.3E+10	1.1E+09	**1.8E+08**
F28	mean	1.1E+107	7.5E+110	1.4E+107	4.0E+94	1.1E+106	6.1E+107	3.5E+106	1.0E+105	3.5E+106	**3.4E+82**
F28	std	1.1E+107	1.6E+111	2.5E+107	6.5E+94	1.8E+106	1.1E+108	2.3E+106	7.6E+104	6.7E+106	**7.3E+82**
F28	min	2.1E+105	7.9E+107	3.8E+105	1.3E+90	3.1E+93	2.1E+104	1.2E+106	2.2E+104	1.5E+97	**3.7E+79**
F28	max	2.5E+107	3.6E+111	5.8E+107	1.5E+95	4.2E+106	2.6E+108	6.3E+106	1.9E+105	1.5E+107	**1.6E+83**
F29	mean	2.5E+09	1.5E+11	4.7E+10	4.5E+09	1.2E+08	1.2E+10	3.5E+07	5.5E+10	2.5E+08	**8.2E+06**
F29	std	6.1E+08	1.9E+10	7.8E+09	2.2E+09	1.2E+08	3.3E+09	**6.2E+06**	1.4E+10	2.2E+08	6.5E+06
F29	min	1.6E+09	1.2E+11	3.6E+10	2.1E+09	1.3E+07	8.5E+09	3.0E+07	3.7E+10	9.8E+07	**2.4E+06**
F29	max	3.1E+09	1.7E+11	5.5E+10	7.5E+09	3.3E+08	1.6E+10	4.4E+07	7.0E+10	6.1E+08	**1.9E+07**

Supplementary Table S13 Composition with Composition Functions 100 dimension

Function	Metrics	AO	AOA	DE	MPA	PSO	FHO	JAYA	NRBO	DBO	CBMA
F30	mean	4.9E+03	2.0E+04	1.4E+04	4.9E+03	**1.1E+03**	8.4E+03	3.0E+03	1.2E+04	6.2E+03	2.1E+03
F30	std	2.4E+03	1.0E+03	1.5E+03	5.8E+02	**4.9E+02**	1.5E+03	1.0E+03	1.6E+03	1.7E+03	6.0E+02
F30	min	3.7E+03	1.9E+04	1.3E+04	4.2E+03	**6.8E+02**	6.1E+03	2.1E+03	1.1E+04	5.2E+03	1.3E+03
F30	max	9.2E+03	2.1E+04	1.7E+04	5.8E+03	**1.9E+03**	1.0E+04	4.6E+03	1.5E+04	9.3E+03	3.0E+03
F31	mean	1.9E+05	4.9E+05	1.1E+05	4.3E+04	9.6E+03	8.5E+04	**5.5E+03**	2.3E+05	7.9E+03	7.0E+03
F31	std	9.6E+04	3.7E+04	4.3E+03	1.9E+04	3.7E+03	9.2E+03	5.3E+03	4.3E+04	**5.2E+02**	1.4E+03
F31	min	7.4E+04	4.5E+05	1.0E+05	2.5E+04	5.1E+03	7.6E+04	**2.2E+03**	2.0E+05	7.3E+03	4.9E+03
F31	max	2.9E+05	5.5E+05	1.1E+05	7.6E+04	1.5E+04	9.6E+04	1.5E+04	3.0E+05	**8.4E+03**	8.7E+03
F32	mean	8.1E+04	8.3E+04	1.0E+05	8.2E+04	1.1E+04	7.6E+04	**2.3E+03**	8.2E+04	5.9E+03	7.1E+03
F32	std	1.1E+04	1.1E+04	1.6E+04	1.1E+04	2.7E+03	6.1E+03	**4.1E+02**	1.1E+04	1.4E+03	8.7E+02
F32	min	6.6E+04	6.8E+04	7.8E+04	6.6E+04	7.3E+03	6.6E+04	**1.9E+03**	6.7E+04	4.4E+03	6.2E+03
F32	max	9.6E+04	9.8E+04	1.2E+05	9.6E+04	1.4E+04	8.2E+04	**3.0E+03**	9.7E+04	8.0E+03	8.4E+03
F33	mean	3.0E+10	4.7E+10	3.8E+10	4.2E+10	1.2E+08	1.2E+10	3.1E+07	4.5E+10	1.5E+09	**5.0E+06**
F33	std	2.5E+10	1.0E+10	7.0E+09	1.0E+10	2.0E+08	2.0E+09	1.2E+07	8.3E+09	2.0E+09	**2.6E+06**
F33	min	2.2E+09	3.4E+10	2.8E+10	2.9E+10	7.8E+06	9.4E+09	2.1E+07	3.3E+10	1.3E+08	**2.3E+06**
F33	max	5.6E+10	6.3E+10	4.5E+10	5.7E+10	4.7E+08	1.4E+10	4.9E+07	5.6E+10	5.0E+09	**8.7E+06**
F34	mean	9.2E+02	5.3E+08	5.3E+02	1.1E+03	4.0E+03	**2.1E+02**	8.9E+02	1.0E+03	4.3E+02	9.8E+02
F34	std	2.1E+02	1.9E+08	9.7E+01	6.8E+01	1.4E+03	**2.2E+01**	6.9E+01	1.9E+02	3.1E+02	4.6E+01
F34	min	6.5E+02	3.4E+08	4.6E+02	1.0E+03	3.0E+03	**1.9E+02**	8.0E+02	7.1E+02	2.0E+02	9.3E+02
F34	max	1.1E+03	8.0E+08	6.4E+02	1.2E+03	6.3E+03	**2.5E+02**	9.6E+02	1.2E+03	8.7E+02	1.1E+03
F35	mean	-1.1E+01	-7.8E+00	-4.0E+00	-1.2E+01	**-1.4E+01**	-9.8E+00	-1.0E+01	-1.1E+01	-1.2E+01	-1.3E+01
F35	std	**1.6E-01**	2.7E+00	1.9E+00	2.9E-01	6.9E-01	7.9E-01	2.7E-01	3.0E-01	8.1E-01	4.7E-01
F35	min	-1.1E+01	-1.0E+01	-6.1E+00	-1.2E+01	**-1.5E+01**	-1.1E+01	-1.1E+01	-1.1E+01	-1.3E+01	-1.3E+01
F35	max	-1.0E+01	-4.2E+00	-1.1E+00	-1.1E+01	**-1.3E+01**	-8.9E+00	-9.8E+00	-1.0E+01	-1.1E+01	-1.2E+01
F36	mean	2.5E-02	6.2E+04	**2.4E-02**	2.5E-02	2.7E-02	1.0E+00	1.0E+00	1.0E+00	1.0E+00	2.5E-02
F36	std	2.7E-04	2.5E+04	3.2E-04	**1.5E-04**	8.0E-04	1.9E-04	1.7E-04	2.1E-04	8.3E-04	1.6E-04
F36	min	2.5E-02	3.2E+04	**2.3E-02**	2.4E-02	2.6E-02	1.0E+00	1.0E+00	1.0E+00	1.0E+00	2.4E-02
F36	max	2.5E-02	8.6E+04	**2.4E-02**	2.5E-02	2.8E-02	1.0E+00	1.0E+00	1.0E+00	1.0E+00	2.5E-02
F37	mean	2.2E+85	4.3E+102	3.5E+83	1.2E+69	1.6E+90	1.1E+83	2.1E+80	4.4E+77	**6.4E+63**	3.9E+74
F37	std	5.0E+85	6.6E+102	5.6E+83	2.7E+69	3.6E+90	2.0E+83	3.9E+80	4.7E+77	**1.4E+64**	8.6E+74
F37	min	3.2E+79	1.1E+98	5.0E+79	1.0E+63	4.4E+79	1.8E+78	2.4E+76	1.0E+76	**1.5E+59**	2.9E+68
F37	max	1.1E+86	1.6E+103	1.3E+84	6.0E+69	8.0E+90	4.6E+83	9.0E+80	1.1E+78	**3.2E+64**	1.9E+75
F38	mean	1.3E+107	6.6E+109	1.1E+106	6.7E+95	5.8E+105	5.7E+107	6.1E+106	7.9E+104	8.6E+106	**1.1E+82**
F38	std	2.1E+107	9.7E+109	6.1E+105	8.5E+95	1.2E+106	6.8E+107	7.2E+106	6.1E+104	1.1E+107	**2.0E+82**
F38	min	5.1E+104	3.8E+107	2.3E+105	6.0E+87	8.9E+98	2.6E+106	9.4E+105	5.5E+103	1.0E+94	**1.6E+76**
F38	max	4.8E+107	2.2E+110	1.7E+106	1.9E+96	2.6E+106	1.4E+108	1.9E+107	1.5E+105	2.1E+107	**4.6E+82**

S2.3 500D

Supplementary Table S14 Composition with Unimodal Functions 500 dimension

Function	Metrics	AO	AOA	DE	MPA	PSO	FHO	JAYA	NRBO	DBO	CBMA
F1	mean	1.1E+12	1.6E+12	1.8E+12	9.9E+11	5.4E+11	1.6E+12	5.8E+11	1.3E+12	7.1E+11	**4.7E+11**
F1	std	4.5E+10	5.0E+10	1.9E+11	4.6E+10	5.2E+10	6.8E+10	**3.2E+10**	5.0E+10	5.1E+10	5.0E+10
F1	min	1.0E+12	1.6E+12	1.6E+12	9.2E+11	4.9E+11	1.5E+12	5.5E+11	1.3E+12	6.6E+11	**4.3E+11**
F1	max	1.1E+12	1.7E+12	2.1E+12	1.0E+12	6.2E+11	1.7E+12	6.2E+11	1.4E+12	7.8E+11	**5.5E+11**
F2	mean	1.5E+06	1.9E+06	3.1E+06	1.5E+06	**1.3E+06**	1.6E+06	2.4E+06	1.5E+06	1.6E+06	2.3E+06
F2	std	7.4E+04	2.2E+05	1.6E+05	6.6E+04	8.9E+04	4.6E+04	2.0E+05	**3.8E+04**	1.6E+05	1.1E+05
F2	min	1.4E+06	1.7E+06	3.0E+06	1.4E+06	**1.2E+06**	1.5E+06	2.1E+06	1.5E+06	1.4E+06	2.2E+06
F2	max	1.6E+06	2.2E+06	3.4E+06	1.6E+06	**1.4E+06**	1.7E+06	2.7E+06	1.6E+06	1.8E+06	2.4E+06

Supplementary Table S15 Composition with Simple Multimodal Functions 500 dimension

Function	Metrics	AO	AOA	DE	MPA	PSO	FHO	JAYA	NRBO	DBO	CBMA
F3	mean	4.3E+11	9.6E+11	2.6E+12	4.7E+11	**2.4E+11**	9.3E+11	3.3E+11	6.9E+11	3.7E+11	**6.0E+11**
F3	std	2.2E+10	4.6E+10	3.3E+11	2.9E+10	5.0E+10	3.9E+10	**1.7E+10**	3.7E+10	4.2E+10	1.4E+11
F3	min	4.0E+11	9.1E+11	2.0E+12	4.3E+11	**1.6E+11**	8.8E+11	3.0E+11	6.6E+11	3.2E+11	**3.9E+11**
F3	max	4.5E+11	1.0E+12	2.8E+12	5.1E+11	**2.9E+11**	9.7E+11	3.5E+11	7.5E+11	4.3E+11	**7.2E+11**
F4	mean	1.1E+06	1.7E+06	1.9E+06	1.0E+06	**6.2E+05**	1.6E+06	5.8E+05	1.3E+06	7.3E+05	**4.8E+05**
F4	std	6.8E+04	9.1E+04	9.1E+04	6.5E+04	**3.6E+04**	1.1E+05	6.6E+04	**7.6E+04**	6.2E+04	5.5E+04
F4	min	9.5E+05	1.5E+06	1.8E+06	9.4E+05	**5.7E+05**	1.5E+06	5.1E+05	1.2E+06	6.7E+05	**4.4E+05**
F4	max	1.1E+06	1.7E+06	2.0E+06	1.1E+06	**6.6E+05**	1.7E+06	6.7E+05	1.4E+06	8.2E+05	**5.7E+05**
F5	mean	2.4E+02	2.5E+02	2.4E+02	2.4E+02	2.3E+02	2.5E+02	2.4E+02	2.4E+02	2.3E+02	**2.3E+02**
F5	std	1.4E+00	8.3E-01	7.6E-01	2.5E+00	2.1E+00	**3.6E-01**	6.1E-01	1.2E+00	2.7E+00	2.0E+00
F5	min	2.4E+02	2.4E+02	2.4E+02	2.3E+02	2.3E+02	2.4E+02	2.4E+02	2.4E+02	2.3E+02	**2.3E+02**
F5	max	2.4E+02	2.5E+02	2.5E+02	2.4E+02	2.4E+02	2.5E+02	2.5E+02	2.4E+02	2.4E+02	**2.3E+02**
F6	mean	1.0E+06	1.6E+06	1.9E+06	9.8E+05	5.7E+05	1.6E+06	5.7E+05	1.3E+06	7.3E+05	**4.6E+05**
F6	std	3.0E+04	3.6E+04	1.4E+05	4.7E+04	8.0E+04	4.9E+04	3.7E+04	**2.9E+04**	4.0E+04	2.9E+04
F6	min	1.0E+06	1.6E+06	1.8E+06	9.1E+05	4.8E+05	1.5E+06	5.1E+05	1.3E+06	6.7E+05	**4.1E+05**
F6	max	1.1E+06	1.7E+06	2.1E+06	1.0E+06	6.8E+05	1.6E+06	6.0E+05	1.4E+06	7.7E+05	**4.7E+05**
F7	mean	1.1E+06	1.7E+06	1.9E+06	1.0E+06	6.4E+05	1.6E+06	**6.0E+05**	1.3E+06	8.5E+05	6.3E+05
F7	std	3.8E+04	5.7E+04	1.4E+05	4.8E+04	2.9E+04	5.0E+04	**1.9E+04**	5.2E+04	2.7E+05	9.0E+04
F7	min	1.0E+06	1.6E+06	1.6E+06	9.6E+05	6.0E+05	1.5E+06	5.8E+05	1.3E+06	6.7E+05	**5.0E+05**
F7	max	1.1E+06	1.7E+06	2.0E+06	1.1E+06	6.7E+05	1.7E+06	**6.2E+05**	1.4E+06	1.3E+06	7.3E+05
F8	mean	4.7E+05	6.2E+05	7.3E+05	3.4E+05	2.9E+05	5.4E+05	6.1E+05	5.1E+05	**2.8E+05**	4.6E+05
F8	std	3.0E+04	2.2E+04	5.3E+04	**1.6E+04**	4.2E+04	1.7E+04	3.2E+04	1.7E+04	1.7E+04	2.3E+04
F8	min	4.2E+05	5.8E+05	6.7E+05	3.3E+05	**2.6E+05**	5.2E+05	5.6E+05	4.8E+05	2.6E+05	4.4E+05
F8	max	4.9E+05	6.4E+05	7.9E+05	3.7E+05	3.5E+05	5.6E+05	6.4E+05	5.3E+05	**3.0E+05**	4.9E+05
F9	mean	8.7E+04	1.3E+05	1.0E+05	7.6E+04	4.5E+04	8.9E+04	4.2E+04	1.0E+05	6.0E+04	**2.7E+04**
F9	std	2.7E+03	2.1E+03	**1.3E+03**	2.5E+03	3.0E+03	3.0E+03	3.7E+03	2.5E+03	8.4E+03	1.9E+03
F9	min	8.5E+04	1.2E+05	1.0E+05	7.4E+04	4.0E+04	8.7E+04	3.6E+04	1.0E+05	4.8E+04	**2.5E+04**
F9	max	9.1E+04	1.3E+05	1.1E+05	7.9E+04	4.7E+04	9.4E+04	4.6E+04	1.1E+05	7.0E+04	**3.0E+04**

Supplementary Table S16 Composition with Component Functions 500 dimension

Function	Metrics	AO	AOA	DE	MPA	PSO	FHO	JAYA	NRBO	DBO	CBMA
F10	mean	5.4E+02	7.6E+06	**0.0E+00**	8.8E-08	**0.0E+00**	1.6E+02	**0.0E+00**	**0.0E+00**	**0.0E+00**	4.2E+00
F10	std	6.3E+02	1.5E+07	**0.0E+00**	9.3E-08	**0.0E+00**	3.3E+02	**0.0E+00**	**0.0E+00**	**0.0E+00**	9.0E+00
F10	min	1.6E+00	1.2E+04	**0.0E+00**	2.0E-09	**0.0E+00**	3.9E+00	**0.0E+00**	**0.0E+00**	**0.0E+00**	1.6E-02
F10	max	1.3E+03	3.4E+07	**0.0E+00**	2.2E-07	**0.0E+00**	7.4E+02	**0.0E+00**	**0.0E+00**	**0.0E+00**	2.0E+01
F11	mean	2.7E+06	5.6E+06	1.8E+06	1.4E+06	7.1E+05	2.5E+06	6.4E+05	1.8E+06	2.0E+06	**6.0E+05**
F11	std	4.0E+05	4.6E+06	8.8E+04	7.1E+04	8.0E+04	9.2E+05	**5.4E+04**	1.5E+05	6.1E+05	1.8E+05
F11	min	2.2E+06	2.5E+06	1.7E+06	1.3E+06	5.9E+05	1.7E+06	5.5E+05	1.7E+06	1.4E+06	**4.5E+05**
F11	max	3.1E+06	1.3E+07	1.9E+06	1.5E+06	8.1E+05	3.7E+06	**6.8E+05**	2.1E+06	2.7E+06	9.0E+05
F12	mean	2.2E+01	2.2E+01	2.2E+01	2.1E+01	**2.1E+01**	2.2E+01	2.2E+01	2.1E+01	2.1E+01	2.1E+01
F12	std	1.4E-02	3.0E-02	1.8E-02	2.4E-02	5.8E-02	**1.3E-02**	1.4E-02	4.3E-02	6.4E-02	3.9E-02
F12	min	2.2E+01	2.2E+01	2.2E+01	2.1E+01	**2.1E+01**	2.2E+01	2.2E+01	2.1E+01	2.1E+01	2.1E+01
F12	max	2.2E+01	2.2E+01	2.2E+01	2.1E+01	**2.1E+01**	2.2E+01	2.2E+01	2.2E+01	2.1E+01	2.1E+01
F13	mean	9.2E+02	9.4E+02	9.0E+02	8.0E+02	7.7E+02	9.3E+02	9.1E+02	9.0E+02	7.1E+02	**6.9E+02**
F13	std	9.8E+00	1.1E+01	8.5E+00	7.4E+00	1.4E+01	**2.8E+00**	5.2E+00	6.3E+00	3.1E+01	1.4E+01
F13	min	9.0E+02	9.3E+02	8.9E+02	7.9E+02	7.6E+02	9.3E+02	9.0E+02	8.9E+02	**6.7E+02**	6.8E+02
F13	max	9.3E+02	9.6E+02	9.1E+02	8.1E+02	7.9E+02	9.3E+02	9.2E+02	9.1E+02	7.4E+02	**7.1E+02**
F14	mean	2.7E+02	4.2E+02	4.5E+02	2.6E+02	1.5E+02	4.1E+02	1.4E+02	3.4E+02	2.1E+02	**9.2E+01**
F14	std	2.2E+01	2.1E+01	5.4E+01	1.0E+01	1.4E+01	1.7E+01	1.2E+01	1.1E+01	7.0E+01	**6.9E+00**
F14	min	2.5E+02	4.0E+02	3.8E+02	2.5E+02	1.3E+02	4.0E+02	1.3E+02	3.3E+02	1.6E+02	**8.5E+01**
F14	max	3.1E+02	4.5E+02	5.2E+02	2.8E+02	1.7E+02	4.4E+02	1.6E+02	3.5E+02	3.3E+02	**1.0E+02**
F15	mean	Inf	Inf	Inf	Inf	Inf	Inf	Inf	Inf	Inf	Inf
F15	std	Inf	Inf	Inf	Inf	Inf	Inf	Inf	Inf	Inf	Inf
F15	min	Inf	Inf	Inf	Inf	Inf	Inf	Inf	Inf	Inf	Inf
F15	max	Inf	Inf	Inf	Inf	Inf	Inf	Inf	Inf	Inf	Inf
F16	mean	1.1E+03	1.7E+03	1.8E+03	1.0E+03	6.0E+02	1.6E+03	6.2E+02	1.4E+03	7.3E+02	**3.9E+02**
F16	std	**3.1E+01**	8.2E+01	1.3E+02	5.5E+01	5.4E+01	7.8E+01	4.3E+01	5.9E+01	8.5E+01	3.9E+01
F16	min	1.1E+03	1.6E+03	1.6E+03	9.8E+02	5.2E+02	1.6E+03	5.6E+02	1.3E+03	6.2E+02	**3.4E+02**
F16	max	1.1E+03	1.8E+03	2.0E+03	1.1E+03	6.5E+02	1.8E+03	6.7E+02	1.5E+03	8.4E+02	**4.3E+02**
F17	mean	1.1E+06	1.7E+06	1.9E+06	1.0E+06	5.8E+05	1.6E+06	6.1E+05	1.4E+06	7.2E+05	**4.0E+05**
F17	std	4.9E+04	3.5E+04	1.4E+05	7.0E+04	**1.9E+04**	3.6E+04	3.7E+04	5.5E+04	6.5E+04	4.9E+04
F17	min	1.0E+06	1.6E+06	1.8E+06	9.6E+05	5.6E+05	1.6E+06	5.5E+05	1.3E+06	6.6E+05	**3.3E+05**
F17	max	1.1E+06	1.7E+06	2.1E+06	1.1E+06	6.1E+05	1.7E+06	6.4E+05	1.4E+06	8.1E+05	**4.4E+05**
F18	mean	3.0E+02	3.0E+02	3.0E+02	2.9E+02	2.8E+02	3.0E+02	3.0E+02	3.0E+02	2.8E+02	**2.8E+02**
F18	std	1.7E+00	1.7E+00	**7.0E-01**	1.3E+00	4.5E+00	8.1E-01	8.0E-01	1.6E+00	9.8E-01	1.5E+00
F18	min	3.0E+02	3.0E+02	3.0E+02	2.9E+02	2.8E+02	3.0E+02	3.0E+02	3.0E+02	2.8E+02	**2.8E+02**
F18	max	3.0E+02	3.1E+02	3.0E+02	2.9E+02	2.9E+02	3.0E+02	3.0E+02	3.0E+02	2.8E+02	**2.8E+02**
F19	mean	3.4E+01	6.1E+01	7.1E+01	2.3E+01	1.4E+01	5.6E+01	6.9E+01	4.8E+01	**1.2E+01**	3.1E+01
F19	std	5.8E+00	3.7E+00	3.7E+00	**5.9E-01**	1.5E+00	2.1E+00	3.1E+00	2.4E+00	2.9E+00	1.7E+00
F19	min	2.5E+01	5.7E+01	6.6E+01	2.2E+01	1.2E+01	5.4E+01	6.6E+01	4.5E+01	**8.3E+00**	2.9E+01
F19	max	4.0E+01	6.6E+01	7.5E+01	2.3E+01	**1.5E+01**	5.9E+01	7.2E+01	5.1E+01	1.6E+01	3.3E+01

Supplementary Table S17 Composition with Hybrid Functions 500 dimension

Function	Metrics	AO	AOA	DE	MPA	PSO	FHO	JAYA	NRBO	DBO	CBMA
F20	mean	2.0E+11	4.6E+11	8.4E+11	2.1E+11	**9.9E+10**	4.0E+11	1.3E+11	2.9E+11	2.6E+11	1.5E+11
F20	std	2.4E+10	8.5E+10	9.7E+10	3.4E+10	1.5E+10	1.9E+10	1.4E+10	**1.3E+10**	1.3E+11	2.8E+10
F20	min	1.8E+11	3.9E+11	7.2E+11	1.9E+11	**8.7E+10**	3.7E+11	1.1E+11	2.7E+11	1.6E+11	1.1E+11
F20	max	2.3E+11	5.9E+11	9.4E+11	2.6E+11	**1.2E+11**	4.2E+11	1.4E+11	3.0E+11	4.3E+11	1.8E+11
F21	mean	4.0E+11	6.4E+11	7.3E+11	4.0E+11	2.4E+11	6.4E+11	2.2E+11	5.3E+11	3.0E+11	**1.4E+11**
F21	std	1.9E+10	2.7E+10	5.0E+10	2.3E+10	1.4E+10	2.3E+10	1.8E+10	3.2E+10	2.1E+10	**1.4E+10**
F21	min	3.8E+11	6.2E+11	6.7E+11	3.5E+11	2.3E+11	6.1E+11	1.9E+11	4.9E+11	2.8E+11	**1.1E+11**
F21	max	4.3E+11	6.8E+11	8.1E+11	4.1E+11	2.6E+11	6.7E+11	2.3E+11	5.7E+11	3.3E+11	**1.5E+11**
F22	mean	4.5E+11	8.0E+11	1.4E+12	4.5E+11	2.5E+11	7.8E+11	3.0E+11	6.1E+11	3.3E+11	**2.4E+11**
F22	std	1.5E+10	2.8E+10	1.4E+11	1.6E+10	3.1E+10	2.9E+10	**7.2E+09**	3.4E+10	2.4E+10	3.7E+10
F22	min	4.3E+11	7.7E+11	1.1E+12	4.3E+11	2.1E+11	7.4E+11	2.9E+11	5.6E+11	2.9E+11	**1.8E+11**
F22	max	4.7E+11	8.3E+11	1.5E+12	4.7E+11	2.9E+11	8.1E+11	3.1E+11	6.4E+11	3.5E+11	**2.8E+11**
F23	mean	9.4E+05	7.7E+05	7.6E+05	5.2E+05	3.1E+05	8.8E+05	2.6E+05	7.0E+05	5.5E+05	**2.0E+05**
F23	std	2.7E+05	2.2E+05	4.3E+04	5.1E+04	3.6E+04	3.3E+05	**3.1E+04**	7.5E+04	1.1E+05	3.4E+04
F23	min	5.7E+05	6.0E+05	7.1E+05	4.4E+05	2.8E+05	6.0E+05	2.4E+05	5.9E+05	3.8E+05	**1.6E+05**
F23	max	1.2E+06	1.1E+06	8.1E+05	5.7E+05	3.5E+05	1.3E+06	3.1E+05	7.6E+05	6.6E+05	**2.5E+05**
F24	mean	1.4E+11	3.0E+11	7.3E+11	1.5E+11	**7.5E+10**	2.9E+11	9.3E+10	2.2E+11	1.1E+11	7.9E+10
F24	std	1.0E+10	1.8E+10	1.3E+11	2.0E+10	**7.1E+09**	1.9E+10	1.1E+10	1.0E+10	1.3E+10	7.8E+09
F24	min	1.3E+11	2.8E+11	6.1E+11	1.4E+11	**6.6E+10**	2.7E+11	8.3E+10	2.0E+11	9.2E+10	7.1E+10
F24	max	1.6E+11	3.2E+11	9.4E+11	1.9E+11	**8.6E+10**	3.1E+11	1.1E+11	2.3E+11	1.3E+11	9.1E+10
F25	mean	Inf	Inf	Inf	Inf	Inf	Inf	Inf	Inf	Inf	Inf
F25	std	Inf	Inf	Inf	Inf	Inf	Inf	Inf	Inf	Inf	Inf
F25	min	Inf	Inf	Inf	Inf	Inf	Inf	Inf	Inf	Inf	Inf
F25	max	Inf	Inf	Inf	Inf	Inf	Inf	Inf	Inf	Inf	Inf
F26	mean	2.1E+11	3.3E+11	3.8E+11	2.0E+11	1.2E+11	3.3E+11	1.1E+11	2.7E+11	1.7E+11	**7.2E+10**
F26	std	5.6E+09	1.2E+10	2.4E+10	1.3E+10	1.2E+10	1.1E+10	4.3E+09	1.1E+10	6.2E+10	**3.4E+09**
F26	min	2.0E+11	3.2E+11	3.5E+11	1.9E+11	1.1E+11	3.1E+11	1.1E+11	2.6E+11	1.2E+11	**6.8E+10**
F26	max	2.2E+11	3.5E+11	4.1E+11	2.2E+11	1.3E+11	3.4E+11	1.2E+11	2.9E+11	2.7E+11	**7.6E+10**
F27	mean	2.0E+11	3.2E+11	3.7E+11	2.0E+11	1.1E+11	3.1E+11	1.1E+11	2.6E+11	1.3E+11	**6.8E+10**
F27	std	**5.9E+09**	1.2E+10	2.6E+10	1.4E+10	9.4E+09	1.1E+10	7.9E+09	1.0E+10	1.3E+10	8.1E+09
F27	min	1.9E+11	3.0E+11	3.3E+11	1.8E+11	9.9E+10	3.0E+11	1.0E+11	2.4E+11	1.1E+11	**5.9E+10**
F27	max	2.1E+11	3.3E+11	4.0E+11	2.2E+11	1.2E+11	3.3E+11	1.2E+11	2.7E+11	1.5E+11	**8.0E+10**
F28	mean	Inf	Inf	Inf	Inf	Inf	Inf	Inf	Inf	Inf	Inf
F28	std	Inf	Inf	Inf	Inf	Inf	Inf	Inf	Inf	Inf	Inf
F28	min	Inf	Inf	Inf	Inf	Inf	Inf	Inf	Inf	Inf	Inf
F28	max	Inf	Inf	Inf	Inf	Inf	Inf	Inf	Inf	Inf	Inf
F29	mean	4.7E+11	6.6E+11	2.4E+12	5.0E+11	**2.5E+11**	6.3E+11	3.2E+11	5.7E+11	4.7E+11	6.8E+11
F29	std	**9.7E+09**	1.3E+10	1.6E+11	1.3E+10	1.9E+10	1.2E+10	3.2E+10	1.4E+10	1.0E+10	2.0E+10
F29	min	4.6E+11	6.4E+11	2.2E+12	4.9E+11	**2.2E+11**	6.1E+11	2.7E+11	5.4E+11	4.5E+11	6.6E+11
F29	max	4.8E+11	6.8E+11	2.6E+12	5.1E+11	**2.6E+11**	6.4E+11	3.6E+11	5.8E+11	4.8E+11	7.1E+11

Supplementary Table S18 Composition with Composition Functions 500 dimension

Function	Metrics	AO	AOA	DE	MPA	PSO	FHO	JAYA	NRBO	DBO	CBMA
F30	mean	9.7E+04	1.2E+05	1.1E+05	7.4E+04	4.7E+04	8.6E+04	4.3E+04	1.1E+05	5.9E+04	**2.8E+04**
F30	std	7.0E+03	**1.2E+03**	3.4E+03	1.4E+03	3.4E+03	2.4E+03	3.2E+03	1.6E+03	8.0E+03	1.8E+03
F30	min	8.9E+04	1.2E+05	1.0E+05	7.2E+04	4.3E+04	8.4E+04	4.1E+04	1.1E+05	4.8E+04	**2.5E+04**
F30	max	1.0E+05	1.2E+05	1.1E+05	7.6E+04	5.1E+04	9.0E+04	4.7E+04	1.1E+05	7.1E+04	**3.0E+04**
F31	mean	1.8E+06	2.6E+06	1.8E+06	1.3E+06	6.1E+05	1.8E+06	1.0E+06	1.5E+06	1.1E+06	**4.6E+05**
F31	std	3.1E+05	3.0E+05	6.9E+04	5.0E+04	**3.2E+04**	2.1E+05	7.7E+05	6.9E+04	2.0E+05	3.2E+04
F31	min	1.5E+06	2.2E+06	1.7E+06	1.3E+06	5.6E+05	1.6E+06	6.2E+05	1.4E+06	8.5E+05	**4.1E+05**
F31	max	2.3E+06	2.9E+06	1.9E+06	1.4E+06	6.4E+05	2.1E+06	2.4E+06	1.6E+06	1.3E+06	**4.9E+05**
F32	mean	4.2E+05	4.2E+05	1.9E+06	4.2E+05	6.4E+05	4.2E+05	9.2E+05	4.2E+05	**4.2E+05**	4.6E+05
F32	std	5.8E+03	5.7E+03	1.9E+05	5.9E+03	7.0E+04	**5.6E+03**	2.7E+04	5.7E+03	7.4E+03	3.9E+04
F32	min	4.2E+05	4.2E+05	1.8E+06	4.1E+05	5.5E+05	4.2E+05	8.9E+05	4.2E+05	**4.1E+05**	4.1E+05
F32	max	4.3E+05	4.3E+05	2.2E+06	**4.3E+05**	7.4E+05	4.3E+05	9.5E+05	4.3E+05	4.3E+05	5.1E+05
F33	mean	1.8E+11	1.8E+11	2.4E+12	1.8E+11	2.3E+11	1.8E+11	3.2E+11	1.8E+11	**1.7E+11**	5.6E+11
F33	std	5.0E+09	4.9E+09	2.4E+11	5.5E+09	2.3E+10	4.9E+09	1.2E+10	5.1E+09	**4.5E+09**	1.7E+11
F33	min	1.8E+11	1.8E+11	2.2E+12	1.7E+11	2.0E+11	1.8E+11	3.1E+11	1.7E+11	**1.7E+11**	3.8E+11
F33	max	1.9E+11	1.9E+11	2.7E+12	1.8E+11	2.6E+11	1.9E+11	3.3E+11	1.8E+11	**1.8E+11**	7.8E+11
F34	mean	5.5E+03	6.8E+09	**5.0E+03**	5.4E+03	9.3E+07	5.5E+03	5.4E+03	5.4E+03	5.3E+03	2.1E+04
F34	std	2.2E+02	5.1E+08	1.8E+02	2.0E+02	1.8E+08	2.2E+02	1.3E+02	1.5E+02	**1.3E+02**	1.9E+03
F34	min	5.3E+03	6.3E+09	**4.7E+03**	5.0E+03	2.3E+06	5.2E+03	5.3E+03	5.3E+03	5.1E+03	1.8E+04
F34	max	5.8E+03	7.5E+09	**5.1E+03**	5.5E+03	4.1E+08	5.8E+03	5.6E+03	5.6E+03	5.5E+03	2.3E+04
F35	mean	-5.8E+00	-4.7E+00	-1.3E+00	-5.9E+00	-6.7E+00	-4.1E+00	-5.7E+00	-5.8E+00	-5.8E+00	**-6.9E+00**
F35	std	6.4E-02	4.5E-01	6.3E-01	5.2E-02	1.5E-01	2.0E+00	**4.4E-02**	6.6E-02	2.3E-01	1.4E-01
F35	min	-5.8E+00	-5.3E+00	-2.3E+00	-6.0E+00	-6.9E+00	-5.6E+00	-5.8E+00	-5.9E+00	-6.1E+00	**-7.0E+00**
F35	max	-5.7E+00	-4.1E+00	-4.9E-01	-5.9E+00	-6.6E+00	-8.5E-01	-5.7E+00	-5.7E+00	-5.5E+00	**-6.7E+00**
F36	mean	1.1E+00	1.4E+06	1.1E+00	**1.1E+00**	3.8E+01	1.1E+00	1.1E+00	1.1E+00	1.1E+00	1.1E+00
F36	std	**2.6E-04**	3.2E+05	3.5E-04	2.9E-04	4.4E+01	4.0E-04	3.9E-04	3.7E-04	8.7E-04	7.3E-04
F36	min	1.1E+00	1.0E+06	1.1E+00	1.1E+00	7.1E+00	1.1E+00	1.1E+00	1.1E+00	**1.1E+00**	1.1E+00
F36	max	1.1E+00	1.7E+06	1.1E+00	**1.1E+00**	1.1E+02	1.1E+00	1.1E+00	1.1E+00	1.1E+00	1.1E+00
F37	mean	Inf	Inf	Inf	Inf	Inf	Inf	Inf	Inf	Inf	Inf
F37	std	Inf	Inf	Inf	Inf	Inf	Inf	Inf	Inf	Inf	Inf
F37	min	Inf	Inf	Inf	Inf	Inf	Inf	Inf	Inf	Inf	Inf
F37	max	Inf	Inf	Inf	Inf	Inf	Inf	Inf	Inf	Inf	Inf
F38	mean	Inf	Inf	Inf	Inf	Inf	Inf	Inf	Inf	Inf	Inf
F38	std	Inf	Inf	Inf	Inf	Inf	Inf	Inf	Inf	Inf	Inf
F38	min	Inf	Inf	Inf	Inf	Inf	Inf	Inf	Inf	Inf	Inf
F38	max	Inf	Inf	Inf	Inf	Inf	Inf	Inf	Inf	Inf	Inf

S2.4 1000D

Supplementary Table S19 Composition with Unimodal Functions 1000 dimension

Function	Metrics	AO	AOA	DE	MPA	PSO	FHO	JAYA	NRBO	DBO	CBMA
F1	mean	2.5E+12	3.3E+12	5.6E+12	2.0E+12	**1.9E+12**	3.2E+12	2.1E+12	2.9E+12	2.2E+12	2.8E+12
F1	std	9.8E+10	6.2E+10	4.3E+11	5.0E+10	6.2E+10	**4.3E+10**	5.5E+10	7.6E+10	7.7E+10	8.2E+10
F1	min	2.4E+12	3.2E+12	5.2E+12	1.9E+12	**1.8E+12**	3.2E+12	2.0E+12	2.8E+12	2.1E+12	2.7E+12
F1	max	2.7E+12	3.3E+12	6.0E+12	2.1E+12	**2.0E+12**	3.3E+12	2.1E+12	3.0E+12	2.2E+12	2.9E+12
F2	mean	3.1E+06	3.6E+06	6.9E+06	**2.8E+06**	3.4E+06	3.2E+06	5.0E+06	3.2E+06	3.3E+06	5.1E+06
F2	std	**6.2E+04**	4.3E+05	3.2E+05	1.3E+05	2.0E+05	6.9E+04	2.0E+05	3.0E+05	2.8E+05	2.3E+05
F2	min	3.0E+06	3.2E+06	6.5E+06	**2.7E+06**	3.1E+06	3.2E+06	4.7E+06	2.9E+06	3.0E+06	4.8E+06
F2	max	3.2E+06	4.3E+06	7.2E+06	**3.1E+06**	3.7E+06	3.3E+06	5.2E+06	3.7E+06	3.7E+06	5.4E+06

Supplementary Table S20 Composition with Simple Multimodal Functions 1000 dimension

Function	Metrics	AO	AOA	DE	MPA	PSO	FHO	JAYA	NRBO	DBO	CBMA
F3	mean	1.3E+12	2.0E+12	9.3E+12	**1.0E+12**	1.2E+12	2.0E+12	1.2E+12	1.7E+12	1.3E+12	3.4E+12
F3	std	3.8E+10	1.2E+11	4.0E+11	6.4E+10	6.9E+10	1.2E+11	**2.3E+10**	8.7E+10	1.3E+11	4.6E+11
F3	min	1.2E+12	1.8E+12	8.7E+12	**9.1E+11**	1.0E+12	1.8E+12	1.2E+12	1.5E+12	1.1E+12	3.0E+12
F3	max	1.3E+12	2.1E+12	9.7E+12	**1.1E+12**	1.2E+12	2.1E+12	1.3E+12	1.8E+12	1.4E+12	4.2E+12
F4	mean	2.6E+06	3.4E+06	6.1E+06	2.1E+06	2.1E+06	3.4E+06	2.2E+06	3.0E+06	2.3E+06	**1.9E+06**
F4	std	9.5E+04	9.8E+04	2.0E+05	6.9E+04	1.2E+05	1.0E+05	7.9E+04	8.6E+04	1.3E+05	**5.1E+04**
F4	min	2.5E+06	3.3E+06	5.8E+06	2.0E+06	1.9E+06	3.3E+06	2.1E+06	2.9E+06	2.1E+06	**1.8E+06**
F4	max	2.8E+06	3.6E+06	6.4E+06	2.2E+06	2.2E+06	3.5E+06	2.2E+06	3.1E+06	2.5E+06	**1.9E+06**
F5	mean	4.9E+02	4.9E+02	4.9E+02	4.7E+02	4.8E+02	4.9E+02	4.9E+02	4.9E+02	4.8E+02	**4.7E+02**
F5	std	8.9E-01	6.3E-01	**5.3E-01**	4.6E+00	2.1E+00	7.1E-01	8.0E-01	6.8E-01	1.4E+00	1.8E+00
F5	min	4.9E+02	4.9E+02	4.9E+02	**4.7E+02**	4.7E+02	4.9E+02	4.9E+02	4.9E+02	4.8E+02	4.7E+02
F5	max	4.9E+02	4.9E+02	4.9E+02	4.8E+02	4.8E+02	4.9E+02	4.9E+02	4.9E+02	4.8E+02	**4.7E+02**
F6	mean	2.6E+06	3.3E+06	5.7E+06	2.1E+06	2.0E+06	3.3E+06	2.2E+06	3.0E+06	2.2E+06	**1.7E+06**
F6	std	9.0E+04	9.3E+04	6.6E+05	8.6E+04	1.0E+05	9.5E+04	8.8E+04	9.9E+04	9.2E+04	**7.3E+04**
F6	min	2.5E+06	3.3E+06	4.9E+06	2.1E+06	1.8E+06	3.2E+06	2.1E+06	2.9E+06	2.1E+06	**1.6E+06**
F6	max	2.7E+06	3.5E+06	6.3E+06	2.3E+06	2.1E+06	3.5E+06	2.3E+06	3.1E+06	2.3E+06	**1.8E+06**
F7	mean	2.6E+06	3.3E+06	5.5E+06	2.1E+06	2.1E+06	3.3E+06	2.1E+06	3.0E+06	2.3E+06	**1.8E+06**
F7	std	5.7E+04	**5.0E+04**	2.9E+05	6.2E+04	7.2E+04	6.8E+04	5.3E+04	7.0E+04	9.6E+04	8.6E+04
F7	min	2.5E+06	3.3E+06	5.1E+06	2.0E+06	1.9E+06	3.2E+06	2.1E+06	2.9E+06	2.2E+06	**1.6E+06**
F7	max	2.7E+06	3.4E+06	5.9E+06	2.2E+06	2.1E+06	3.3E+06	2.2E+06	3.1E+06	2.4E+06	**1.9E+06**
F8	mean	1.0E+06	1.3E+06	1.8E+06	**7.0E+05**	8.6E+05	1.1E+06	1.3E+06	1.1E+06	7.6E+05	1.0E+06
F8	std	4.9E+04	2.8E+04	5.7E+04	3.5E+04	4.5E+04	2.0E+04	6.8E+04	2.7E+04	6.6E+04	**2.0E+04**
F8	min	9.6E+05	1.2E+06	1.8E+06	**6.7E+05**	7.8E+05	1.1E+06	1.2E+06	1.0E+06	7.0E+05	9.9E+05
F8	max	1.1E+06	1.3E+06	1.9E+06	**7.5E+05**	9.1E+05	1.1E+06	1.4E+06	1.1E+06	8.7E+05	1.0E+06
F9	mean	2.0E+05	2.6E+05	2.3E+05	1.5E+05	1.3E+05	1.8E+05	1.3E+05	2.3E+05	1.4E+05	**1.1E+05**
F9	std	5.7E+03	5.3E+03	**2.8E+03**	6.3E+03	8.9E+03	6.1E+03	5.0E+03	4.4E+03	7.3E+03	4.0E+03
F9	min	1.9E+05	2.5E+05	2.3E+05	1.4E+05	1.2E+05	1.7E+05	1.2E+05	2.2E+05	1.3E+05	**1.1E+05**
F9	max	2.1E+05	2.6E+05	2.4E+05	1.6E+05	1.5E+05	1.9E+05	1.4E+05	2.3E+05	1.5E+05	**1.2E+05**

Supplementary Table S21 Composition with Component Functions 1000 dimension

Function	Metrics	AO	AOA	DE	MPA	PSO	FHO	JAYA	NRBO	DBO	CBMA
F10	mean	6.4E+01	2.8E+06	**0.0E+00**	4.4E-13	**0.0E+00**	3.2E+01	**0.0E+00**	**0.0E+00**	**0.0E+00**	1.9E+03
F10	std	5.0E+01	3.5E+06	**0.0E+00**	7.8E-13	**0.0E+00**	2.1E+01	**0.0E+00**	**0.0E+00**	**0.0E+00**	2.6E+03
F10	min	9.6E+00	5.0E+05	**0.0E+00**	**0.0E+00**	**0.0E+00**	1.1E+00	**0.0E+00**	**0.0E+00**	**0.0E+00**	9.0E+00
F10	max	1.4E+02	8.8E+06	**0.0E+00**	1.8E-12	**0.0E+00**	5.0E+01	**0.0E+00**	**0.0E+00**	**0.0E+00**	5.7E+03
F11	mean	4.9E+06	5.7E+06	4.7E+06	2.5E+06	2.3E+06	4.5E+06	2.5E+06	3.4E+06	4.1E+06	**2.3E+06**
F11	std	1.6E+06	2.5E+06	1.5E+05	2.7E+05	**1.1E+05**	1.4E+06	1.2E+05	2.5E+05	8.6E+05	6.6E+05
F11	min	3.0E+06	3.4E+06	4.5E+06	2.1E+06	2.2E+06	3.2E+06	2.3E+06	3.1E+06	3.1E+06	**1.7E+06**
F11	max	7.0E+06	8.6E+06	4.8E+06	2.7E+06	**2.4E+06**	6.7E+06	2.6E+06	3.8E+06	5.4E+06	3.4E+06
F12	mean	2.2E+01	2.2E+01	2.2E+01	2.1E+01	**2.1E+01**	2.2E+01	2.2E+01	2.2E+01	2.1E+01	2.1E+01
F12	std	1.4E-02	7.6E-03	1.3E-02	1.9E-02	3.9E-02	**6.1E-03**	9.6E-03	3.3E-02	6.8E-02	3.7E-02
F12	min	2.2E+01	2.2E+01	2.2E+01	2.1E+01	**2.1E+01**	2.2E+01	2.2E+01	2.2E+01	2.1E+01	2.1E+01
F12	max	2.2E+01	2.2E+01	2.2E+01	2.1E+01	**2.1E+01**	2.2E+01	2.2E+01	2.2E+01	2.1E+01	2.1E+01
F13	mean	1.9E+03	1.9E+03	1.9E+03	1.7E+03	1.7E+03	1.9E+03	1.9E+03	1.9E+03	1.6E+03	**1.6E+03**
F13	std	2.0E+01	8.6E+00	8.5E+00	1.4E+01	2.5E+01	**8.0E+00**	1.4E+01	1.5E+01	5.6E+01	3.6E+01
F13	min	1.9E+03	1.9E+03	1.9E+03	1.7E+03	1.7E+03	1.9E+03	1.9E+03	1.8E+03	**1.6E+03**	1.6E+03
F13	max	1.9E+03	1.9E+03	1.9E+03	**1.7E+03**	1.7E+03	1.9E+03	1.9E+03	1.9E+03	1.7E+03	1.7E+03
F14	mean	6.3E+02	8.2E+02	1.5E+03	5.1E+02	4.9E+02	8.2E+02	5.1E+02	7.3E+02	5.6E+02	**4.7E+02**
F14	std	2.1E+01	2.6E+01	3.6E+01	2.0E+01	2.0E+01	2.6E+01	**1.8E+01**	3.2E+01	2.8E+01	3.5E+01
F14	min	6.0E+02	7.8E+02	1.4E+03	4.8E+02	4.6E+02	7.8E+02	4.9E+02	6.8E+02	5.3E+02	**4.3E+02**
F14	max	6.6E+02	8.5E+02	1.5E+03	5.3E+02	**5.1E+02**	8.4E+02	5.4E+02	7.5E+02	5.9E+02	5.3E+02
F15	mean	Inf	Inf	Inf	Inf	Inf	Inf	Inf	Inf	Inf	Inf
F15	std	Inf	Inf	Inf	Inf	Inf	Inf	Inf	Inf	Inf	Inf
F15	min	Inf	Inf	Inf	Inf	Inf	Inf	Inf	Inf	Inf	Inf
F15	max	Inf	Inf	Inf	Inf	Inf	Inf	Inf	Inf	Inf	Inf
F16	mean	1.3E+03	1.7E+03	3.0E+03	1.1E+03	1.0E+03	1.7E+03	1.1E+03	1.5E+03	1.1E+03	**9.2E+02**
F16	std	4.1E+01	4.8E+01	2.4E+02	4.3E+01	4.2E+01	4.4E+01	2.4E+01	**2.4E+01**	3.1E+01	3.1E+01
F16	min	1.2E+03	1.6E+03	2.6E+03	1.0E+03	9.5E+02	1.6E+03	1.0E+03	1.5E+03	1.1E+03	**8.8E+02**
F16	max	1.3E+03	1.7E+03	3.2E+03	1.1E+03	1.1E+03	1.7E+03	1.1E+03	1.5E+03	1.2E+03	**9.5E+02**
F17	mean	2.5E+06	3.3E+06	5.9E+06	2.0E+06	2.0E+06	3.3E+06	2.1E+06	2.9E+06	2.2E+06	**1.8E+06**
F17	std	1.0E+05	9.5E+04	5.4E+05	**5.2E+04**	1.5E+05	1.1E+05	1.1E+05	6.5E+04	7.6E+04	1.0E+05
F17	min	2.4E+06	3.2E+06	5.0E+06	2.0E+06	1.8E+06	3.2E+06	2.0E+06	2.9E+06	2.1E+06	**1.6E+06**
F17	max	2.7E+06	3.5E+06	6.4E+06	2.1E+06	2.2E+06	3.5E+06	2.2E+06	3.0E+06	2.3E+06	**1.9E+06**
F18	mean	6.1E+02	6.1E+02	6.1E+02	5.8E+02	5.8E+02	6.1E+02	6.1E+02	6.1E+02	5.9E+02	**5.8E+02**
F18	std	2.6E+00	1.1E+00	1.1E+00	3.7E+00	2.8E+00	**5.7E-01**	6.1E-01	1.7E+00	4.3E+00	2.4E+00
F18	min	6.0E+02	6.1E+02	6.1E+02	5.8E+02	5.8E+02	6.1E+02	6.1E+02	6.0E+02	5.8E+02	**5.7E+02**
F18	max	6.1E+02	6.2E+02	6.1E+02	5.9E+02	5.8E+02	6.1E+02	6.1E+02	6.1E+02	5.9E+02	**5.8E+02**
F19	mean	4.3E+01	6.5E+01	8.1E+01	**2.2E+01**	2.7E+01	5.9E+01	7.2E+01	5.7E+01	2.3E+01	2.9E+01
F19	std	2.5E+00	1.5E+00	2.6E+00	1.6E+00	2.1E+00	3.7E+00	**9.9E-01**	3.8E+00	5.5E+00	1.9E+00
F19	min	4.0E+01	6.4E+01	7.8E+01	2.1E+01	2.3E+01	5.5E+01	7.1E+01	5.2E+01	**1.9E+01**	2.7E+01
F19	max	4.6E+01	6.7E+01	8.5E+01	**2.5E+01**	2.8E+01	6.4E+01	7.3E+01	6.1E+01	3.2E+01	3.1E+01

Supplementary Table S22 Composition with Hybrid Functions 1000 dimension

Function	Metrics	AO	AOA	DE	MPA	PSO	FHO	JAYA	NRBO	DBO	CBMA
F20	mean	5.3E+11	8.5E+11	2.6E+12	**4.3E+11**	4.8E+11	7.9E+11	5.2E+11	6.8E+11	6.0E+11	1.0E+12
F20	std	**1.4E+10**	7.8E+10	2.7E+11	3.5E+10	1.5E+10	2.7E+10	3.0E+10	3.3E+10	8.3E+10	9.2E+10
F20	min	5.2E+11	7.8E+11	2.2E+12	**3.8E+11**	4.7E+11	7.5E+11	4.8E+11	6.4E+11	5.3E+11	9.2E+11
F20	max	5.5E+11	9.7E+11	2.8E+12	**4.6E+11**	5.0E+11	8.2E+11	5.6E+11	7.2E+11	7.3E+11	1.1E+12
F21	mean	1.0E+12	1.3E+12	2.2E+12	8.2E+11	8.1E+11	1.3E+12	8.5E+11	1.2E+12	9.3E+11	**7.4E+11**
F21	std	3.5E+10	2.8E+10	2.8E+11	4.6E+10	4.1E+10	2.9E+10	2.3E+10	**2.0E+10**	4.4E+10	3.0E+10
F21	min	9.7E+11	1.3E+12	2.0E+12	7.6E+11	7.8E+11	1.3E+12	8.2E+11	1.1E+12	8.6E+11	**7.0E+11**
F21	max	1.1E+12	1.4E+12	2.5E+12	8.9E+11	8.8E+11	1.3E+12	8.8E+11	1.2E+12	9.7E+11	**7.7E+11**
F22	mean	1.2E+12	1.6E+12	4.5E+12	**9.4E+11**	1.0E+12	1.6E+12	1.1E+12	1.4E+12	1.1E+12	1.7E+12
F22	std	3.1E+10	2.2E+10	3.6E+11	3.7E+10	7.4E+10	2.8E+10	**1.6E+10**	1.8E+10	2.5E+10	9.3E+10
F22	min	1.1E+12	1.6E+12	3.9E+12	**8.8E+11**	9.3E+11	1.6E+12	1.0E+12	1.4E+12	1.1E+12	1.6E+12
F22	max	1.2E+12	1.6E+12	4.8E+12	**9.6E+11**	1.1E+12	1.6E+12	1.1E+12	1.4E+12	1.1E+12	1.8E+12
F23	mean	1.5E+06	1.5E+06	1.9E+06	9.5E+05	9.0E+05	1.4E+06	1.0E+06	1.3E+06	1.1E+06	**8.1E+05**
F23	std	3.2E+05	2.6E+05	7.0E+04	8.4E+04	**3.2E+04**	2.3E+05	6.0E+04	1.1E+05	2.2E+05	6.6E+04
F23	min	1.2E+06	1.3E+06	1.8E+06	8.2E+05	8.6E+05	1.3E+06	9.4E+05	1.1E+06	8.8E+05	**7.2E+05**
F23	max	1.9E+06	1.9E+06	2.0E+06	1.0E+06	9.5E+05	1.8E+06	1.1E+06	1.5E+06	1.4E+06	**9.1E+05**
F24	mean	3.8E+11	5.9E+11	2.8E+12	**3.0E+11**	3.6E+11	5.8E+11	3.6E+11	4.9E+11	3.9E+11	6.9E+11
F24	std	5.7E+09	1.0E+10	1.1E+11	**3.3E+09**	8.7E+09	1.0E+10	1.3E+10	1.4E+10	3.4E+10	6.6E+10
F24	min	3.7E+11	5.8E+11	2.6E+12	**3.0E+11**	3.5E+11	5.7E+11	3.4E+11	4.8E+11	3.5E+11	6.4E+11
F24	max	3.9E+11	6.0E+11	2.9E+12	**3.1E+11**	3.7E+11	6.0E+11	3.8E+11	5.1E+11	4.4E+11	7.8E+11
F25	mean	Inf	Inf	Inf	Inf	Inf	Inf	Inf	Inf	Inf	Inf
F25	std	Inf	Inf	Inf	Inf	Inf	Inf	Inf	Inf	Inf	Inf
F25	min	Inf	Inf	Inf	Inf	Inf	Inf	Inf	Inf	Inf	Inf
F25	max	Inf	Inf	Inf	Inf	Inf	Inf	Inf	Inf	Inf	Inf
F26	mean	5.1E+11	6.7E+11	1.2E+12	4.1E+11	4.0E+11	6.6E+11	4.3E+11	6.0E+11	4.6E+11	**3.5E+11**
F26	std	1.3E+10	1.1E+10	1.2E+11	6.5E+09	2.1E+10	**5.2E+09**	7.0E+09	1.0E+10	5.5E+10	1.5E+10
F26	min	4.9E+11	6.5E+11	1.0E+12	4.0E+11	3.6E+11	6.5E+11	4.3E+11	5.8E+11	3.9E+11	**3.4E+11**
F26	max	5.2E+11	6.8E+11	1.3E+12	4.2E+11	4.2E+11	6.6E+11	4.4E+11	6.1E+11	5.2E+11	**3.7E+11**
F27	mean	5.2E+11	6.7E+11	1.1E+12	4.1E+11	4.0E+11	6.6E+11	4.3E+11	5.9E+11	4.3E+11	**3.3E+11**
F27	std	2.4E+10	2.3E+10	8.4E+10	2.5E+10	1.6E+10	2.0E+10	**7.9E+09**	1.8E+10	1.4E+10	2.1E+10
F27	min	4.9E+11	6.4E+11	1.0E+12	3.8E+11	3.8E+11	6.4E+11	4.3E+11	5.7E+11	4.2E+11	**3.1E+11**
F27	max	5.5E+11	7.0E+11	1.2E+12	4.5E+11	4.2E+11	6.9E+11	4.5E+11	6.2E+11	4.5E+11	**3.6E+11**
F28	mean	Inf	Inf	Inf	Inf	Inf	Inf	Inf	Inf	Inf	Inf
F28	std	Inf	Inf	Inf	Inf	Inf	Inf	Inf	Inf	Inf	Inf
F28	min	Inf	Inf	Inf	Inf	Inf	Inf	Inf	Inf	Inf	Inf
F28	max	Inf	Inf	Inf	Inf	Inf	Inf	Inf	Inf	Inf	Inf
F29	mean	1.1E+12	1.3E+12	9.1E+12	**1.0E+12**	1.3E+12	1.3E+12	1.4E+12	1.2E+12	1.1E+12	3.7E+12
F29	std	5.9E+10	7.6E+10	5.5E+11	7.5E+10	1.1E+11	6.8E+10	1.1E+11	7.1E+10	**5.9E+10**	4.9E+11
F29	min	1.0E+12	1.2E+12	8.4E+12	**9.2E+11**	1.1E+12	1.2E+12	1.3E+12	1.1E+12	1.0E+12	3.1E+12
F29	max	1.2E+12	1.4E+12	9.8E+12	**1.1E+12**	1.4E+12	1.4E+12	1.6E+12	1.3E+12	1.2E+12	4.5E+12

Supplementary Table S23 Composition with Composition Functions 1000 dimension

Function	Metrics	AO	AOA	DE	MPA	PSO	FHO	JAYA	NRBO	DBO	CBMA
F30	mean	2.1E+05	2.5E+05	2.3E+05	1.5E+05	1.4E+05	1.8E+05	1.3E+05	2.3E+05	1.3E+05	**1.2E+05**
F30	std	4.2E+03	1.1E+04	3.3E+03	5.7E+03	4.0E+03	**1.9E+03**	4.5E+03	3.5E+03	3.0E+03	2.8E+03
F30	min	2.1E+05	2.3E+05	2.2E+05	1.5E+05	1.3E+05	1.7E+05	1.2E+05	2.3E+05	1.3E+05	**1.1E+05**
F30	max	2.2E+05	2.6E+05	2.3E+05	1.6E+05	1.4E+05	1.8E+05	1.4E+05	2.3E+05	1.4E+05	**1.2E+05**
F31	mean	3.4E+06	5.4E+06	6.0E+06	2.8E+06	2.0E+06	3.7E+06	3.3E+06	3.2E+06	3.2E+06	**1.8E+06**
F31	std	3.4E+05	2.4E+05	4.5E+05	1.1E+05	9.9E+04	8.2E+05	1.6E+06	**8.5E+04**	1.7E+05	1.0E+05
F31	min	3.1E+06	5.1E+06	5.2E+06	2.7E+06	1.9E+06	3.2E+06	2.4E+06	3.1E+06	2.9E+06	**1.7E+06**
F31	max	3.8E+06	5.7E+06	6.3E+06	3.0E+06	2.1E+06	5.2E+06	6.1E+06	3.3E+06	3.4E+06	**1.9E+06**
F32	mean	8.4E+05	8.4E+05	6.0E+06	**8.4E+05**	2.0E+06	8.4E+05	2.0E+06	8.4E+05	8.4E+05	1.8E+06
F32	std	2.2E+04	2.2E+04	2.8E+05	2.2E+04	9.8E+04	**2.2E+04**	9.2E+04	2.2E+04	2.2E+04	7.8E+04
F32	min	8.2E+05	8.2E+05	5.7E+06	8.1E+05	1.9E+06	8.2E+05	1.9E+06	8.2E+05	**8.1E+05**	1.7E+06
F32	max	8.6E+05	8.6E+05	6.4E+06	**8.5E+05**	2.2E+06	8.6E+05	2.1E+06	8.6E+05	8.5E+05	1.8E+06
F33	mean	4.0E+11	4.1E+11	9.4E+12	**3.9E+11**	1.3E+12	4.1E+11	9.6E+11	4.0E+11	3.9E+11	3.6E+12
F33	std	1.5E+10	1.5E+10	4.5E+11	1.6E+10	7.8E+10	1.5E+10	7.9E+10	1.5E+10	**1.5E+10**	2.4E+11
F33	min	3.8E+11	3.8E+11	8.7E+12	**3.7E+11**	1.2E+12	3.8E+11	8.8E+11	3.8E+11	3.7E+11	3.3E+12
F33	max	4.2E+11	4.3E+11	9.8E+12	**4.1E+11**	1.4E+12	4.3E+11	1.1E+12	4.2E+11	4.1E+11	3.9E+12
F34	mean	1.1E+04	1.6E+10	**1.0E+04**	1.1E+04	4.2E+08	1.1E+04	1.1E+04	1.1E+04	1.0E+04	2.9E+05
F34	std	4.5E+02	1.3E+09	2.3E+02	**1.5E+02**	3.5E+08	2.6E+02	1.6E+02	2.8E+02	3.0E+02	5.3E+04
F34	min	1.0E+04	1.4E+10	1.0E+04	1.0E+04	1.3E+08	1.0E+04	1.1E+04	1.0E+04	**1.0E+04**	2.1E+05
F34	max	1.1E+04	1.8E+10	**1.1E+04**	1.1E+04	8.5E+08	1.1E+04	1.1E+04	1.1E+04	1.1E+04	3.4E+05
F35	mean	-4.5E+00	-3.7E+00	-8.5E-01	-4.7E+00	**-5.0E+00**	-3.6E+00	-4.5E+00	-4.5E+00	-4.5E+00	-4.7E+00
F35	std	3.6E-02	4.5E-01	4.9E-01	**1.1E-02**	4.9E-02	1.7E+00	3.1E-02	6.7E-02	1.9E-01	1.7E-01
F35	min	-4.6E+00	-4.3E+00	-1.4E+00	-4.7E+00	**-5.1E+00**	-4.8E+00	-4.5E+00	-4.7E+00	-4.8E+00	-4.8E+00
F35	max	-4.5E+00	-3.3E+00	-2.1E-01	-4.7E+00	**-5.0E+00**	-8.0E-01	-4.5E+00	-4.5E+00	-4.3E+00	-4.4E+00
F36	mean	1.2E+00	4.0E+06	1.2E+00	**1.2E+00**	2.8E+04	1.2E+00	1.2E+00	1.2E+00	1.2E+00	1.3E+00
F36	std	5.3E-04	6.6E+05	5.1E-04	**2.4E-04**	1.9E+04	3.9E-04	4.9E-04	3.6E-04	1.2E-03	9.4E-03
F36	min	1.2E+00	3.1E+06	1.2E+00	**1.2E+00**	3.9E+03	1.2E+00	1.2E+00	1.2E+00	1.2E+00	1.3E+00
F36	max	1.3E+00	4.7E+06	1.2E+00	**1.2E+00**	5.6E+04	1.2E+00	1.2E+00	1.2E+00	1.2E+00	1.3E+00
F37	mean	Inf	Inf	Inf	Inf	Inf	Inf	Inf	Inf	Inf	Inf
F37	std	Inf	Inf	Inf	Inf	Inf	Inf	Inf	Inf	Inf	Inf
F37	min	Inf	Inf	Inf	Inf	Inf	Inf	Inf	Inf	Inf	Inf
F37	max	Inf	Inf	Inf	Inf	Inf	Inf	Inf	Inf	Inf	Inf
F38	mean	Inf	Inf	Inf	Inf	Inf	Inf	Inf	Inf	Inf	Inf
F38	std	Inf	Inf	Inf	Inf	Inf	Inf	Inf	Inf	Inf	Inf
F38	min	Inf	Inf	Inf	Inf	Inf	Inf	Inf	Inf	Inf	Inf
F38	max	Inf	Inf	Inf	Inf	Inf	Inf	Inf	Inf	Inf	Inf

S3 Modeling

The simulation requires modeling of the room. The dartboard and the robot arm, and other obstruction elements were located in the room. The environment was measured with a laser rangefinder. The dartsboard has standard sizes. The robot's attributes were extracted from its datasheet [S2]. The dart camera was modeled based on images. Measurements to identify darts were performed with two cameras. Each camera had a 1280×720 pixel resolution and 240 frames per second recording speed. The videos have been aligned and segmented.

S3.1 Kinematic Robot Model

The robot model is created using parameters for the arm's length, the robot's Denavit–Hartenberg implementation, each joint's motion range, maximum speeds, and accelerations. Trapezoidal velocity functions approximated the robot's dynamics in the absence of further data. The measurement data observed delays, ramp-up, overshoot, and various constraints. The robot's accuracy was estimated from measurements of the position and speed of the joints. Initial position, orientation, and velocities are required for a simple throwing task. The direct robot model provides the Tool Center Point's 12 parameters for the dart model's initial data.

S3.2 Dynamic Dart Model

The applied dart model extends James's and Potts's model [S3] with continuous integration and three more DoF. This model contains the geometric and material parameters of darts. Projection images of darts were used to determine their dimensions and geometry, as shown in Figure S1. Measurements were performed on the dart to reliably apply the dynamic dart model and reduce the measurement uncertainty and approximate the flow parameters. Based on the measurements, the model was identified.

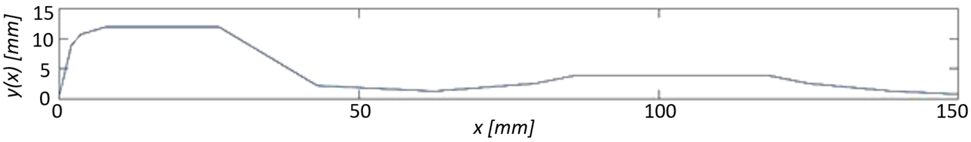

Supplementary Figure S1 A projection of half a dart.

Inner and external coordinate systems are used to describe the dart motion. The first coordinate system is fixed to the robot base, and the second one moves together with the darts. In this way, the absolute position is known, and calculating the forces and the torques is easier. The differential surface area's force is calculated and integrated symbolically on the darts area to calculate the forces and torques. The differential surface area's forces are calculated traditionally.

The two main parts of the air drag force are the 'form drag' and the 'skin friction'. The calculation of the force (d**F**) is presented in Eq. (S1).

$$\mathrm{d}\mathbf{F} = -(p - p_\infty)\mathrm{d}\mathbf{A} + \tau_0 \mathbf{e}|\mathrm{d}\mathbf{A}|, \tag{S1}$$

where $\mathrm{d}\mathbf{A}$ is the surface element, p is the pressure on the given surface element, p_∞ is the pressure of the resting fluid, τ_0 is the shear stress, and $\mathbf{e}$ is the unit vector pointing to the direction of the flowing fluid. Equation (S1) can be transformed into Eq. (S2) by introducing some factors.

$$\mathrm{d}\mathbf{F} = -\frac{1}{2}\rho|\mathbf{v}_\infty|\Big(|\mathbf{v}_\infty|\mathrm{c}_p\mathrm{d}\mathbf{A} - \mathbf{v}_\infty\mathrm{c}_f|\mathrm{d}\mathbf{A}|\Big), \tag{S2}$$

where $\mathbf{v}_\infty$ is the velocity of the flowing fluid, ρ is the density, c_p is the pressure factor, and c_f is the friction factor. The torque (d**M**) can be calculated based on the force as presented in Eq. (S3).

$$\mathrm{d}\mathbf{M} = \mathbf{r}_{dA} \times (\mathrm{d}\mathbf{F})|\mathrm{d}\mathbf{A}|, \tag{S3}$$

where $\mathbf{r}_{dA}$ is a surface vector pointing to the surface from the center of gravity. Constant form factors and friction factors are applied for the dart's parts. The darts are separated into three parts based on their shape: tip, cylindrical body (barrel, shaft), and flight. Before the simulation, the forces and the torques were symbolically integrated and grouped according to six degrees of freedom. The simulation overview is shown in Fig. S2.

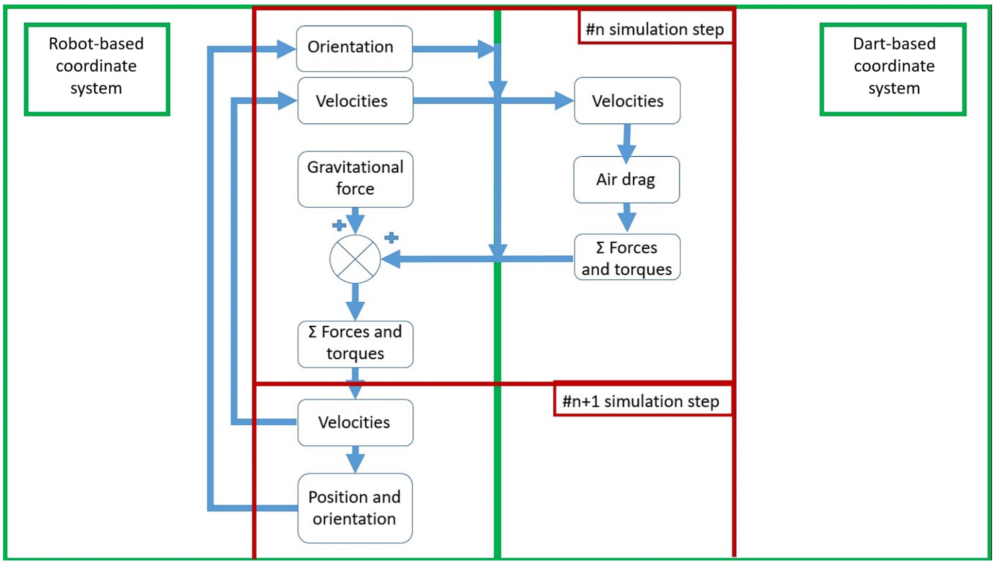

Supplementary Figure S2 Simplified simulation overview. Left: Robot based coordinate system, Right: Center of gravity of darts based coordinate system

Supplementary Table S24 Human-like Throw

distance [m]	3
initial velocity [m/s]	5. . . 7
initial velocity angel [°]	10. . . 30
initial angular velocity [1°/s]	-900. . . 100
initial pitch [°]	5. . . 50

The model was implemented in MATLAB Simulink. The solver was preset to ode113, with 1e-8 initial step size, 1e-10 minimum step size, and 1e-6 maximum step size based on the human-like throw (Table S24). Human-like throwing data are based on research by James et al. extended to robot dimensions [S3]. The simulation parameters were not relaxed for faster evaluation because uncertainty could lead to optimization in the wrong direction. The robot has different dimensions, leading to a wider initial parameter range. Farther from the human-like throw, the simulation may become unstable. Additional early stop criteria were applied to prevent incorrect and time-consuming simulations. The Simulink implementation and parts of the model are shown in Figures S3 and S4.

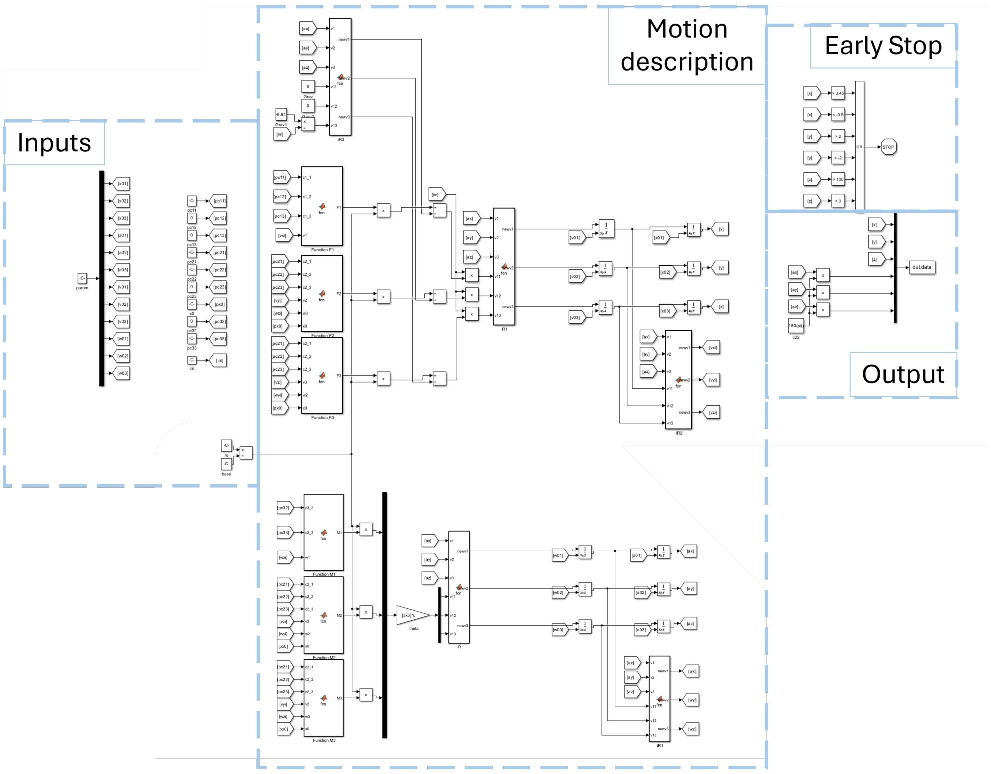

Supplementary Figure S3 Simulation overview in MATLAB Simulink [S4].

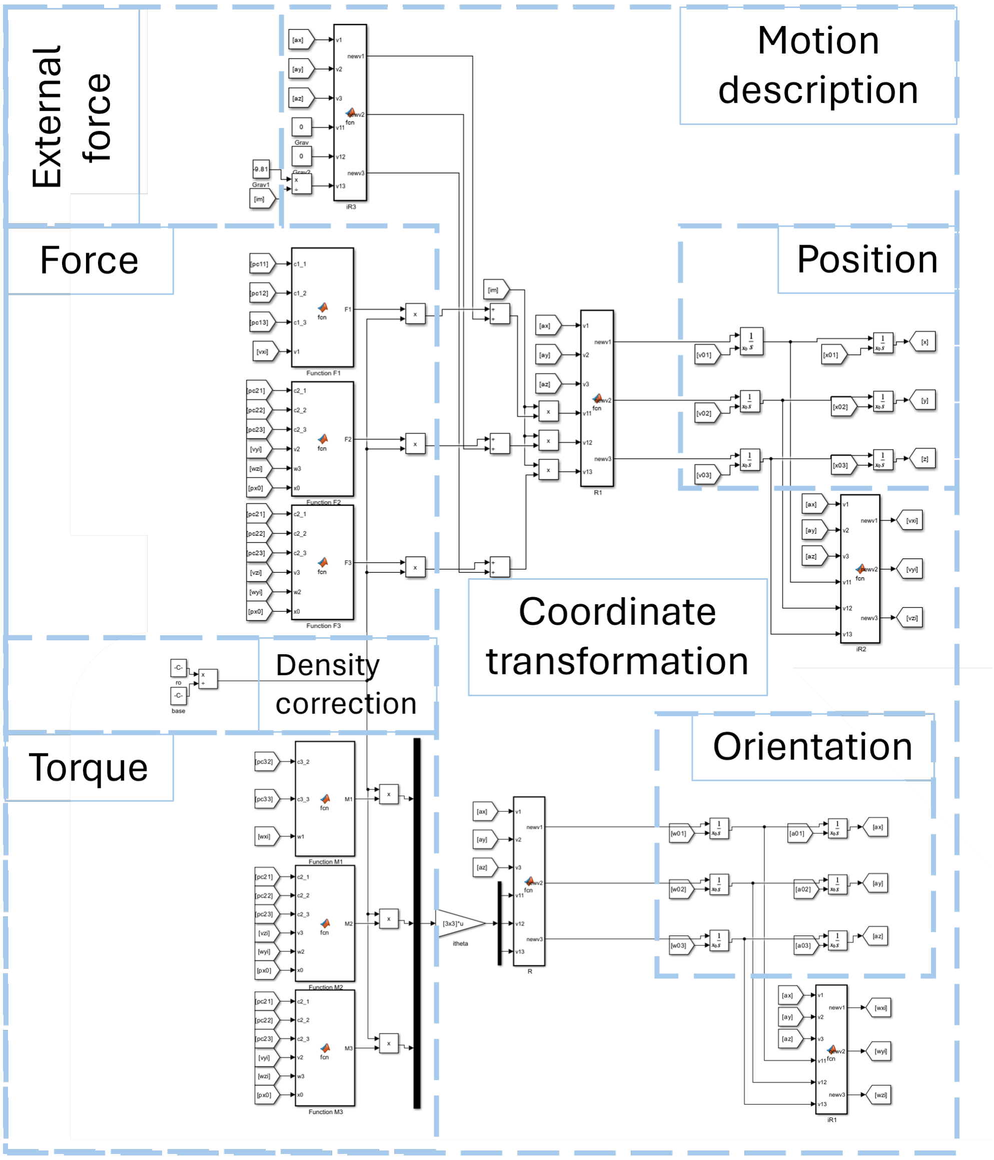

Supplementary Figure S4 Motion description in MATLAB Simulink [S4].

The model was first identified for use. Figure S5 shows the dart model mean absolute error values and the 95% confidence interval of the five measured degrees of freedom. Rotation about the longitudinal axis of the dart was not measured. This is the most accurate dart model from the available literature, considering the errors. The error of the model contains approximations and measurement uncertainty. Although the model allows for a safe optimization of the throw, it is necessary to optimize in

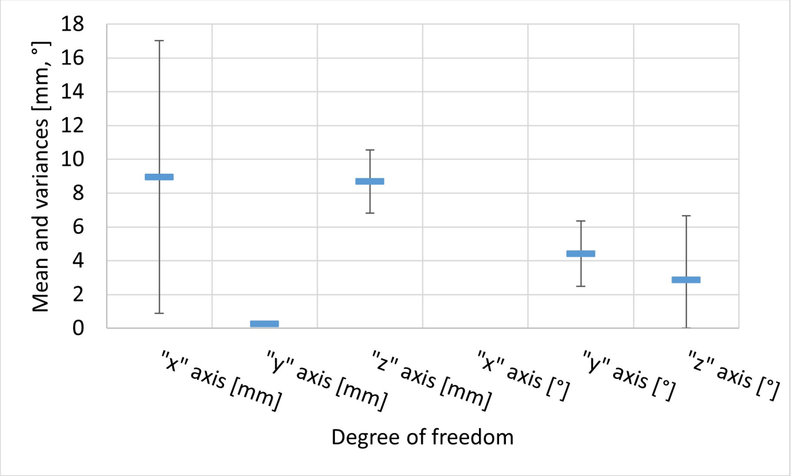

Supplementary Figure S5 Dart model's mean absolute error values and the 95% confidence interval of the five measured degrees of freedom (rotation around the "x" axis was not measured)

the real world. The model's primary goal is to find the style of effective throwing and approach the desired region.

Once the models were ready, they were placed in the simulation environment. The external coordinate system was fixed to the robot's base, Figure S6. Another advantage of modeling is that the part models can be easily interchanged.

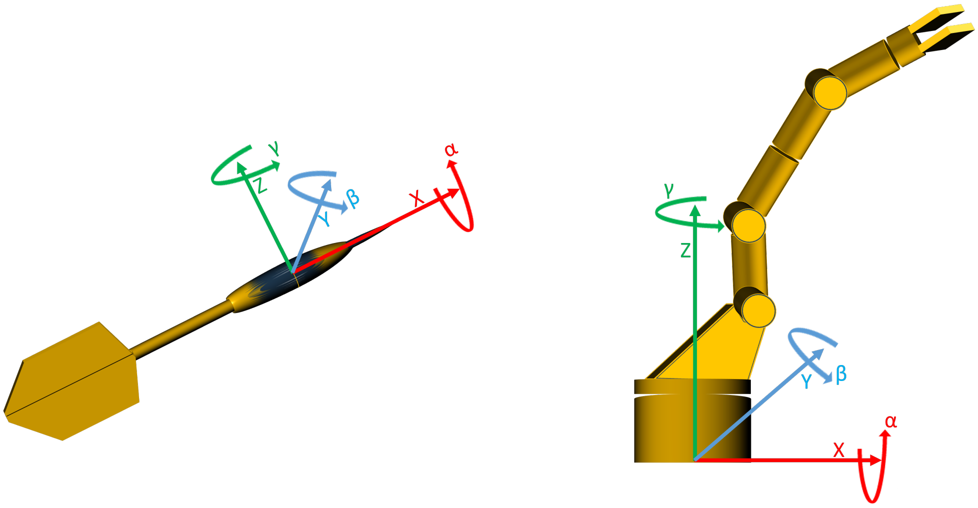

Supplementary Figure S6 A 3D illustration of dart- and robot-based coordinate system [S5]. The illustrative 3D figures were created in Microsoft PowerPoint (Version 2501) using simple shapes [S6]. The mathematical models used in the framework are also available as indicated in the Code Availability statement.

References

[S1] Awad, N., Ali, M., Liang, J., Qu, B. & Suganthan, P. Problem definitions and evaluation criteria for the cec 2017 special session and competition on single objective real-parameter numerical optimization (2016).

[S2] Robot parameters. https://www.kuka.com/-/media/kuka-downloads/imported/8350ff3ca11642998dbdc81dcc2ed44c/db_kr_6_en.pdf?rev=114c88a839ed40918205db5a948b12db&hash=05BFFA13F7CB1D07666A3A136840655B (2025). Accessed: 2025-02-06.

[S3] James, D. & Potts, J. Experimental validation of dynamic stability analysis applied to dart flight. *Sports Engineering* **21**, 347–358 (2018).

[S4] MathWorks. Matlab (2019). URL https://www.mathworks.com/. Initially created using MATLAB R2019b (Version 9.7) and later updated using MATLAB R2023a (Version 9.14), Accessed: 2025-02-14.

[S5] Szilárd Kovács. Illostrative 3d models. https://gitlab.inf.elte.hu/CBMA_Darts_Playing_Robot/darts-playing-robot/-/tree/master/Figures_illustrations?ref_type=heads (2025). Accessed: 2025-02-14.

[S6] Microsoft Corporation. Microsoft powerpoint (2025). URL https://www.microsoft.com/en-us/microsoft-365/powerpoint. Version 2501 (Build 18429.20158), Accessed: 2025-02-14.
